# Galectin-3 aggravates microglial activation and tau transmission in tauopathy

**DOI:** 10.1172/JCI165523

**Published:** 2024-01-16

**Authors:** Jian Jing Siew, Hui-Mei Chen, Feng-Lan Chiu, Chia-Wei Lee, Yao-Ming Chang, Hung-Lin Chen, Thi Ngoc Anh Nguyen, Hung-Ting Liao, Mengyu Liu, Hsiao-Tien Hagar, Yung-Chen Sun, Hsing-Lin Lai, Min-Hao Kuo, David Blum, Luc Buée, Lee-Way Jin, Shih-Yu Chen, Tai-Ming Ko, Jie-Rong Huang, Hung-Chih Kuo, Fu-Tong Liu, Yijuang Chern

**Affiliations:** 1 Institute of Biomedical Sciences; 2 Institute of Cellular and Organismic Biology, Academia Sinica, Taipei, Taiwan.; 3 Department of Biochemistry and Molecular Biology, Michigan State University, East Lansing, Michigan, USA.; 4 Institute of Biochemistry and Molecular Biology, National Yang Ming Chiao Tung University, Taipei, Taiwan.; 5 Univ. Lille, Inserm, CHU Lille, U1172 - LilNCog - Lille Neuroscience & Cognition, Lille, France.; 6 Alzheimer & Tauopathies, LabEx DISTALZ, LiCEND, Lille, France.; 7 Department of Pathology and Laboratory Medicine, University of California Davis, Sacramento, California, USA.; 8 Department of Biological Science and Technology, National Yang Ming Chiao Tung University, Hsinchu, Taiwan.

**Keywords:** Neuroscience, Alzheimer disease, Neurodegeneration, iPS cells

## Abstract

Alzheimer’s disease is characterized by the accumulation of amyloid-β plaques, aggregation of hyperphosphorylated tau (pTau), and microglia activation. Galectin-3 (Gal3) is a β-galactoside–binding protein that has been implicated in amyloid pathology. Its role in tauopathy remains enigmatic. Here, we showed that Gal3 was upregulated in the microglia of humans and mice with tauopathy. pTau triggered the release of Gal3 from human induced pluripotent stem cell–derived microglia in both its free and extracellular vesicular–associated (EV-associated) forms. Both forms of Gal3 increased the accumulation of pathogenic tau in recipient cells. Binding of Gal3 to pTau greatly enhanced tau fibrillation. Besides Gal3, pTau was sorted into EVs for transmission. Moreover, pTau markedly enhanced the number of EVs released by iMGL in a Gal3-dependent manner, suggesting a role of Gal3 in biogenesis of EVs. Single-cell RNA-Seq analysis of the hippocampus of a mouse model of tauopathy (THY-Tau22) revealed a group of pathogenic tau-evoked, Gal3-associated microglia with altered cellular machineries implicated in neurodegeneration, including enhanced immune and inflammatory responses. Genetic removal of Gal3 in THY-Tau22 mice suppressed microglia activation, reduced the level of pTau and synaptic loss in neurons, and rescued memory impairment. Collectively, Gal3 is a potential therapeutic target for tauopathy.

## Abstract 2

Gal3 participates in the regulation of extracellular vesicles, the promotion of pathogenic tau fibrillation, and the activation of microglia.

## Graphical abstract

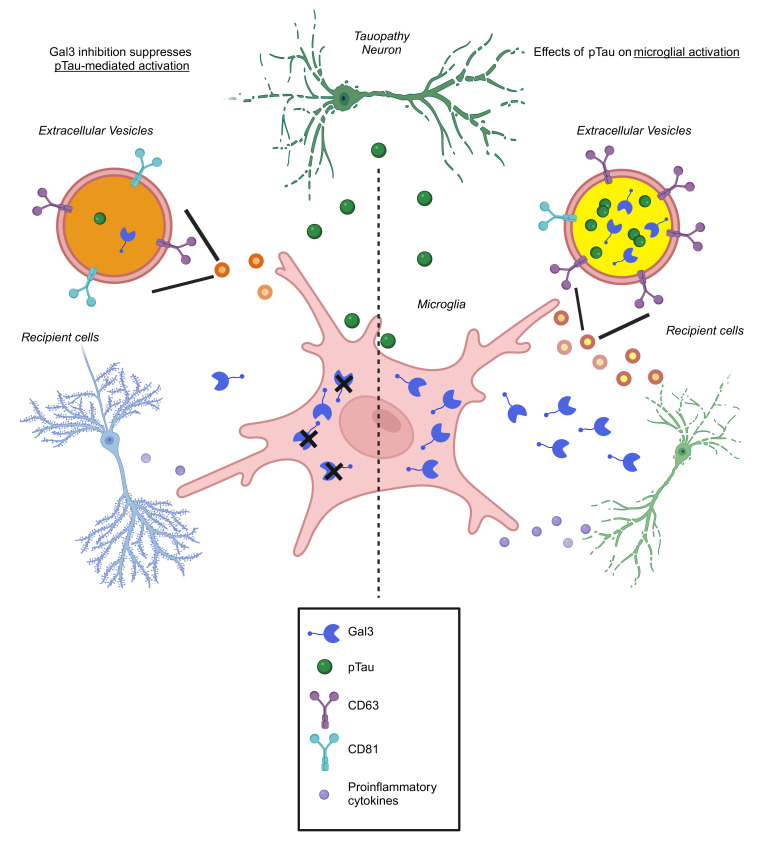

## Introduction

Neuronal inclusions consisting of the microtubule-associated protein tau are found in various neurodegenerative diseases known as tauopathies. Alzheimer’s disease (AD) is the most prevalent tauopathy and is characterized by the accumulation of extracellular β-amyloid (Aβ) plaques and intracellular misfolded tau, which forms neurofibrillary tangles, causing microglial activation and synaptic loss (1, 2). Most AD cases are sporadic, while a small portion of patients with AD carry genes that cause increased Aβ production and subsequently induce progressive neurodegeneration and microglial activation (3). Many drugs that target Aβ plaques have proven ineffective in the treatment of AD in clinical trials. Recently, the FDA approved Aducanumab and Lecanemab, both are monoclonal antibody–based therapies targeting Aβ and show moderate improvements in cognitive decline for patients with early AD (4). However, the efficacy and potential side effects of these treatments remain to be monitored. While Aβ plaque accumulation is frequently observed in aging brains, it doesn’t necessarily lead to the development of AD. For example, a neuroimaging study of 513 participants demonstrated that individuals with both amyloid and tau pathologies exhibited a 27% prevalence of AD. In contrast, those with only amyloid pathology had just 6% prevalence (5), underscoring the significant role of other factors, like tau, in AD development. Moreover, genetic and epigenetic studies of patients with AD reveal that many genes associated with AD risk are enriched in microglia and are related to immune responses (6, 7).

Microglia are immune cells in the brain that play important roles in brain development, homeostasis maintenance, and diseases (8). Accumulating evidence shows that dysregulated microglial activation and altered homeostatic functions contribute to neurodegeneration (9, 10). Earlier studies demonstrated that microglia and neurons interact through multiple routes, such as direct contact, the formation of tunneling nanotubes, and the secretion of exosomes or soluble factors (such as cytokines and chemokines) (11). Despite the tremendous efforts that have been devoted to the investigation of microglial-neuronal interactions, the current understanding of the role of microglia in tauopathy remains incomplete. This is complicated by the fact that microglia exhibit multidimensional phenotypes, including immune activation and suppression, during the course of neurodegenerative diseases (12). Moreover, microglia express multiple types of lectins (e.g., galectins) that serve as critical checkpoints for microglial activation and contribute to neurodegeneration (13, 14).

The role of Galectin-3 (Gal3), encoded by the *Lgals3* gene, in neurodegenerative diseases has recently attracted much attention (15). Gal3 is a proinflammatory autocrine mediator that can bind toll-like receptor 4 (TLR4) and the receptor expressed on myeloid cells-2 (TREM2) (1, 14). In a model of Huntington’s disease, microglial Gal3 has been shown to accumulate on damaged lysosomes, interfering with their clearance activity and facilitating inflammation (16). A large-scale proteomic analysis of the brains of individuals with AD recently identified Gal3 in a microglial metabolism module enriched with genetic risk factors of AD (17). Furthermore, comparison analysis of models representing AD, amyotrophic lateral sclerosis (ALS), and multiple sclerosis showed that microglia in neurodegenerative contexts displayed elevated levels of genes associated with the *Trem2-Apoe* pathway, including *Lgals3* and *Clec7a*, which contribute to the development of detrimental microglial phenotypes (18). Moreover, Gal3 knockout was demonstrated to reduce the plaque burden and improve cognitive behaviors in 2 transgenic mouse models of AD (APP/PS1 and 5xFAD) (1, 19). Nevertheless, the roles of Gal3 in tauopathies (including AD and frontotemporal lobar dementia [FTLD]), which involve amnestic cognitive impairment and neuroinflammation, remain unclear.

## Results

### Upregulation of microglial Gal3 in tauopathy.

**Figure 1:**
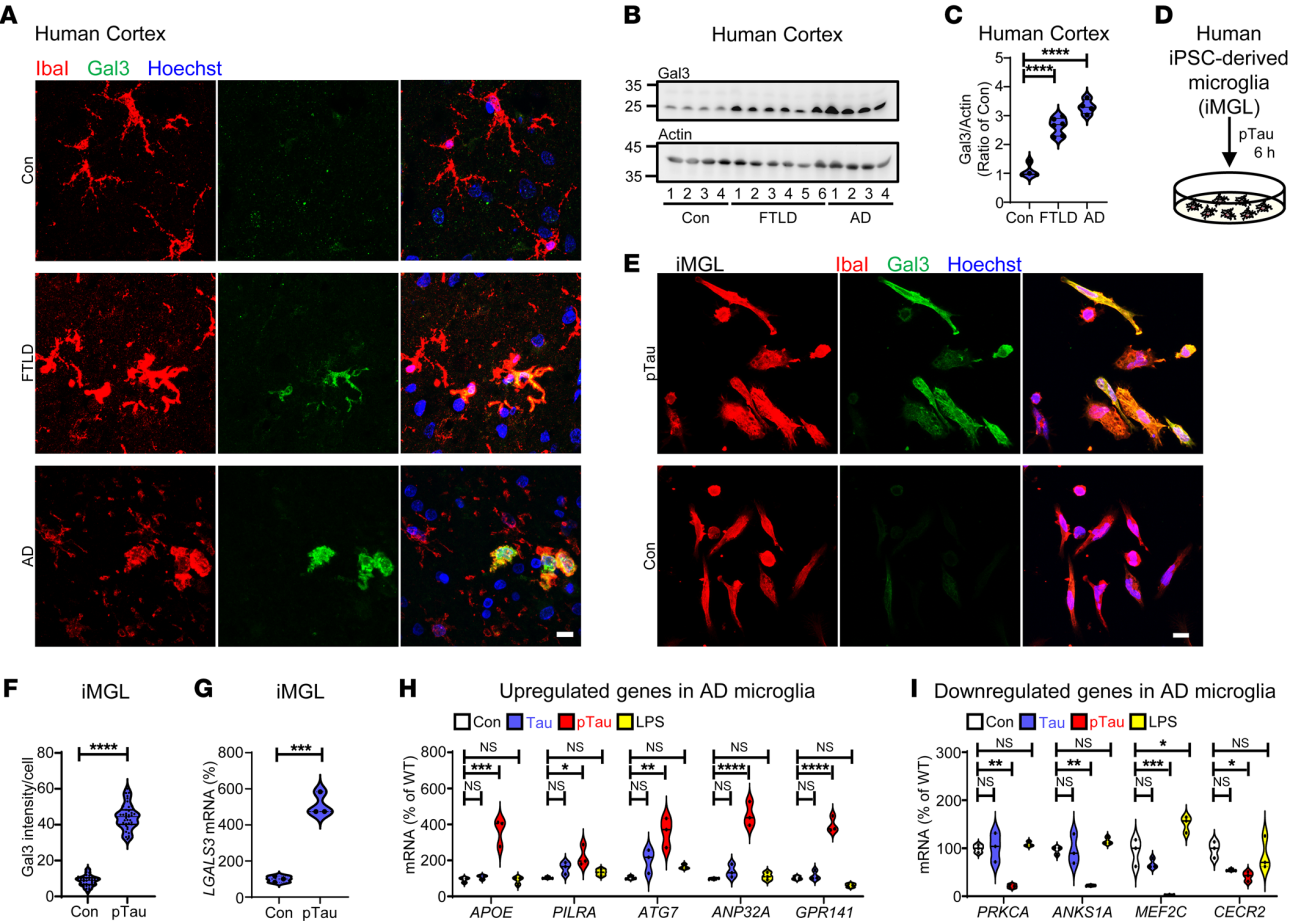
Pathogenic tau upregulates Gal3 in microglia. (**A**) Immunochemical staining of Gal3 in microglia in the cortexes of patients with frontotemporal lobar dementia (FTLD; Braak score 5) or Alzheimer’s disease (AD; Braak score 6) and those of people in the control group (Con; Braak score 1). (**B** and **C**) Immunoblot detection of Gal3 in FTLD, AD and Con samples, *n* = 4 for Con and AD, *n* = 6 for FTLD. (**D** and **E**) Schematic diagram illustrating the study protocol in **E** and immunofluorescence staining of Gal3 and a microglial marker (IbaI) in iPSC-derived microglia (iMGL) prepared from 44 days of differentiation and treated with 50 nM of pTau for 6 hours. (**F**) Quantification of the data in **E**, *n* = 3 iMGL lines, 3 coverslips per iMGL line, 3–4 fields per coverslip. (**G**) q-RT-PCR analysis of *LGALS3* in iMGLs treated with pTau for 6 hours, *n* = 3 iMGL lines. q-RT-PCR analysis of (**H**) *APOE*, *PILRA*, *ATG7*, *ANP32A,* and *GPR141*, and (**I**) *PRKCA*, *ANKS1A*, *MEF2C,* and *CECR2* in iMGLs treated with tau (50 nM), pTau (50 nM), and LPS (100 ng/mL) for 6 hours, *n* = 3 iMGL lines. Scale bars: 10μm. Data in **F** and **G** were analyzed by 2-tailed unpaired *t* test, **C**, **H** and **I** were analyzed by 1-way with Tukey’s test, respectively. Violin plots show medians with 25th and 75th percentiles, *P < 0.05, **P < 0.01, ***P < 0.001, ****P < 0.0001.

To characterize the role of Gal3 in patients with tauopathy, we analyzed cortical and hippocampal samples from individuals with FTLD and AD (Supplemental Table 1; supplemental material available online with this article; https://doi.org/10.1172/JCI165523DS1). As expected, AT8 immune-positive signals were found only in diseased brains. In addition, Gal3 expression was elevated in microglia, as indicated by IbaI-positive cells (Figure 1, A–C, and Supplemental Figure 1, A and B). Some Gal3-positive microglia were observed in areas surrounding AT8-positive soma and/or dystrophic neurites (Supplemental Figure 1A). To assess whether exposure to pathogenic tau from degenerating neurons activates microglia in tauopathy brains, we first differentiated 3 independent human induced pluripotent stem cell (iPSC) lines into microglia (iMGLs, (20)) (Supplemental Figure 2 and Supplemental Table 2). Treatment of these iMGL with a very low level of recombinant hyperphosphorylated tau (pTau, WT, 1N4R (21); 50 nM; Supplemental Figure 3) for 6 hours significantly upregulated Gal3 protein and transcript levels (Figure 1, D–G). No obvious intracellular Gal3 puncta were observed in these Gal3-containing iMGL (Figure 1E). Importantly, treating iMGLs with pTau, but not with tau or LPS, for 6 hours recapitulated the abnormal upregulation of genes (APOE, PILRA, ATG7, ANP32A, and GPR141) (Figure 1H) and downregulation of genes (PRKCA, ANPS1A, MEF2C, and CECR2) (Figure 1I) observed in the microglia of patients with AD (10, 22). This suggests that our in vitro model using pTau-treated iMGL is an appropriate model of the AD context.

### Gal3 early responsive genes.

**Figure 2:**
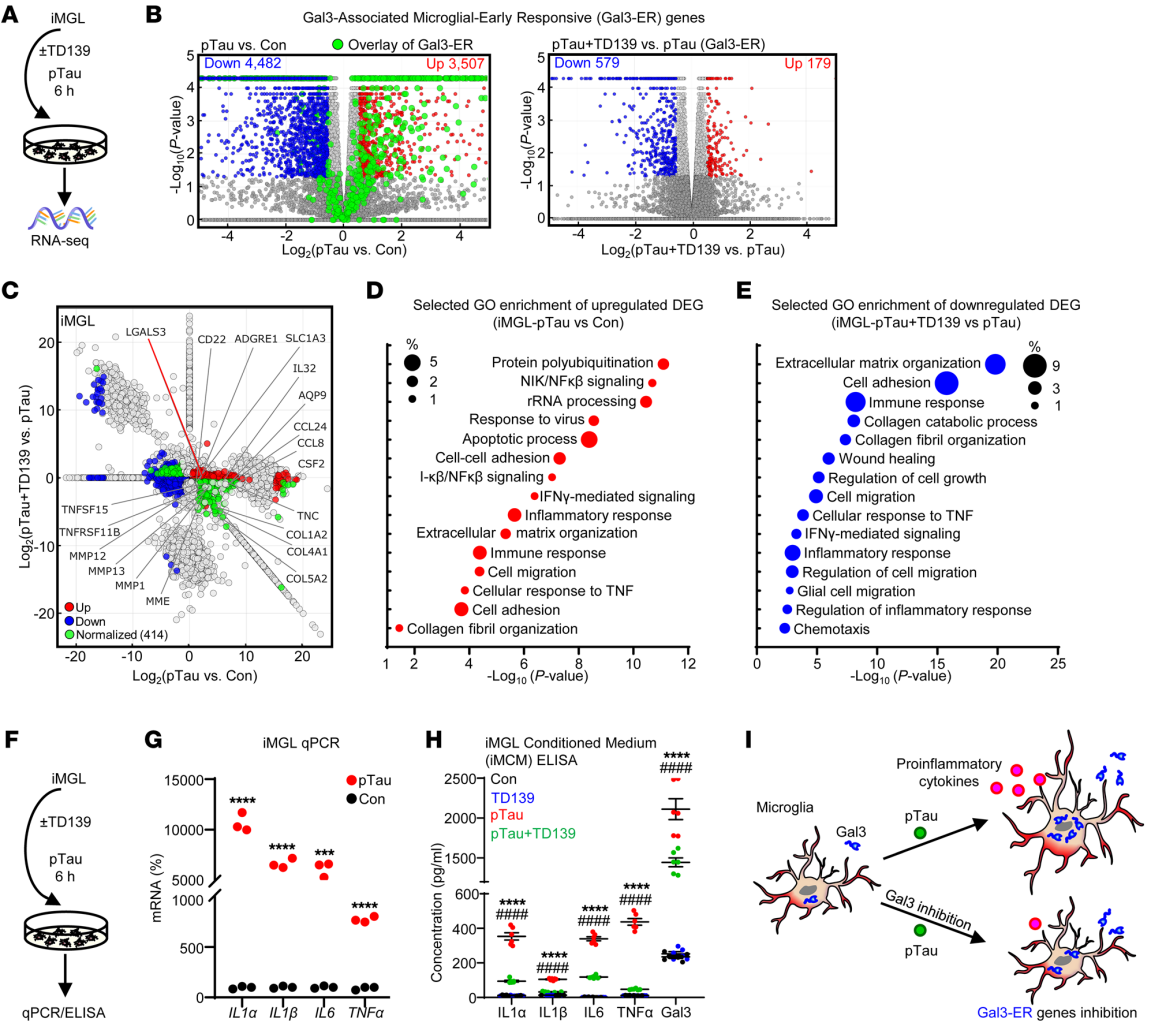
Pathogenic tau triggers microglial activation via Gal3. (**A**) RNA-Seq analysis of iMGL pretreated for 24 hours with TD139 (10 μM, Gal3 inhibition) and 6 hours with pTau (50 nM) and controls. (**B**) Volcano plots show the DEGs identified in the pTau versus Con and pTau plus TD139 versus pTau groups. Red and blue dots indicate upregulated and downregulated DEGs, respectively, and enlarged green dots indicate Gal3-ER genes. Cutoff of significance, |Log_2_ FC| > 0.55 and *P* < 0.05 (**C**) Differential expression analysis of the RNA-Seq data in (**B**), Log_2_ FC in pTau versus Con (x-axis) and pTau+TD139 versus pTau (y-axis). Upregulated DEGs are shown in the right part (pTau-activated genes) or upper part (TD139-activated genes) of the graph, and downregulated DEGs are shown in the left part (pTau-inhibited genes) or lower part (TD139-inhibited genes) of the graph. Red dots and blue dots indicate upregulated and downregulated DEGs, respectively, while green dots indicate DEGs normalized by Gal3 inhibition (TD139). (**D**) GO enrichment analysis of DEGs upregulated under early pTau effects in iMGL identified by RNA-Seq. (**E**) GO enrichment analysis of downregulated DEGs identified in pTau plus TD139 versus pTau iMGL. (**F** and **G**) q-RT-PCR analysis of proinflammatory genes (*IL1*α, *IL1*β, *IL6,* and *TNF*α) in iMGL treated with pTau for 6 hours, *n* = 3 iMGL line, ***P < 0.001, ****P < 0.0001. (**H**) ELISA of conditioned medium collected from iMGL (iMCM) treated with pTau and a Gal3 inhibitor, *n* = 6, 3 iMGL lines from 2 independent differentiations, pTau versus Con ****P < 0.0001, and pTau+TD139 versus pTau, ####*P* < 0.0001. (**I**) Schematic diagram illustrating the effect of pTau and Gal3 inhibition on microglial activation.

To assess the critical roles of Gal3 in the initial microglial response to pathogenic tau, and its subsequent activation, iMGL prepared from 3 independent control iPSC lines were treated with recombinant pTau and subjected to RNA-Seq (Figure 2A). The function of Gal3 was inhibited by treatment with TD139, a cell-permeable Gal3 inhibitor (16). In total, 7,989 differentially expression genes (DEGs; 3,507 upregulated and 4,482 downregulated) were identified between the pTau and Control treatments, and 758 DEGs (179 upregulated and 579 downregulated) were identified between the pTau-plus-TD139 and pTau treatments (Figure 2, B and C and Supplemental Table 3). Transcriptomic profiling further revealed that pTau-induced canonical pathways in iMGLs closely resembled those observed in AD, as seen in other experimental models of the disease (Supplemental Figure 4, A and B). Pathway analysis showed that treatment with pTau elevated multiple pathways (including the activation of protein polyubiquitination, NIK/NFκβ signaling, and inflammatory responses) (Figure 2D, Supplemental Figure 4D), and suppressed a few other pathways (including cell division, DNA replication, and metabolic pathways) (Supplemental Figure 4, C and E). Gal3 was among the immediate early genes upregulated in iMGL in response to pTau treatment. Inhibition of Gal3 by TD139 normalized a subgroup of DEGs (green dots, Figure 2C) triggered by pTau. Specifically, 758 DEGs were identified between the pTau-plus-TD139 and pTau treatments and were designated as Gal3-early responsive genes (Gal3-ER genes) (Figure 2B and Supplemental Figure 5). These Gal3-ER genes were enriched in the pathways of extracellular matrix organization, cell adhesion, and immune response (Figure 2E and Supplemental Figure 4, F and G).

Consistent with the upregulation of Gal3 in iMGL in the initial phase of pTau exposure (Figure 1G), the mRNA levels of Gal3 and several proinflammatory cytokines were increased in the iMGL-derived conditioned medium (iMCM) (Figure 2, F–H), all being markedly reduced after treatment with TD139, suggesting that Gal3 plays a role in mediating the inflammatory response evoked by pTau in iMGL (Figure 2, H and I).

Previous studies have identified a cluster of disease-associated microglia (DAM) in an AD mouse models, as well as a group of microglia known as MGnD (18, 23). The latter exhibit conserved microglial properties across experimental models of AD, ALS, and multiple sclerosis. Microglia exhibiting upregulated levels of Apoe and Gal3 are known to exhibit neurodegenerative phenotypes, and Gal3 is known to stimulate the TREM2-DAP12 signaling during microglial activation (1, 24). Furthermore, a recent study examining the relationship between TREM2 and ApoE4, both risk factors for AD, found exacerbated neurodegeneration in P301S tau mice with TREM2 knockout and ApoE4 expression. The findings indicate that TREM2-independent microgliosis could facilitate tau-mediated neurodegeneration when ApoE4 is present (25). In the current study, our analysis indicates that, among the DEGs induced by pTau in iMGL, 15.2% of the upregulated DEGs and 16.6% of the downregulated DEGs are shared within DAM genes (Supplemental Figure 4H). Similarly, 32.1% of the upregulated DEGs and 52.9% of the downregulated DEGs are shared with MGnD genes (Supplemental Figure 4I and Supplemental Table 4). Through qPCR analysis, we found that pTau upregulated the level of *APOE* and downregulated the level of *TREM2*, while TD139 treatment did not reverse these effects of pTau (Supplemental Figure 4, J and K). As a control, treatment with TD139 alone for 6 hours caused 205 upregulated genes and 132 downregulated genes (Supplemental Figure 4L). Nevertheless, these genes did not exhibit significant enrichment in specific biological processes as observed in the gene ontology (GO) pathway analysis using DAVID Bioinformatics Resources (https://david.ncifcrf.gov/). Principal component analysis of the overall samples is shown in Supplemental Figure 4M.

To assess the expression profiles of Gal3-ER genes, we selected 8 genes based on RNA-Seq analysis of iMGLs treated with pTau for 6 hours (Figure 2C). We then examined their expression patterns in iMGLs using quantitative real-time PCR (RT–qPCR) after treating them with pTau for 6, 24, 48, or 72 hours, in either the absence or presence of TD139. The upregulation of all 8 Gal3-ER genes by pTau was sensitive to TD139 treatment following a 48-hour exposure to pTau (Supplemental Figure 6). Half of these Gal3-ER genes (*AQP9*, *MMP1*, *MMP13*, and *TNFSF15*) remained sensitive to TD139 after 72 hours of treatment with pTau (Supplemental Figure 6A), while the other half (*ADGRE1*, *SLC1A3*, *CCL8*, and *TNFSF11b*) were no longer sensitive to TD139 following a 72-hour treatment with pTau (Supplemental Figure 6B). These results suggest that Gal3 likely plays a critical role in the early phase following treatment with pTau. Additional pathways may be activated to further regulate the pTau-mediated changes in the transcriptomic profile of iMGLs.

### pTau-activated microglia release Gal3 in free and EV-associated forms, both contributing to the development of tauopathy.

**Figure 3:**
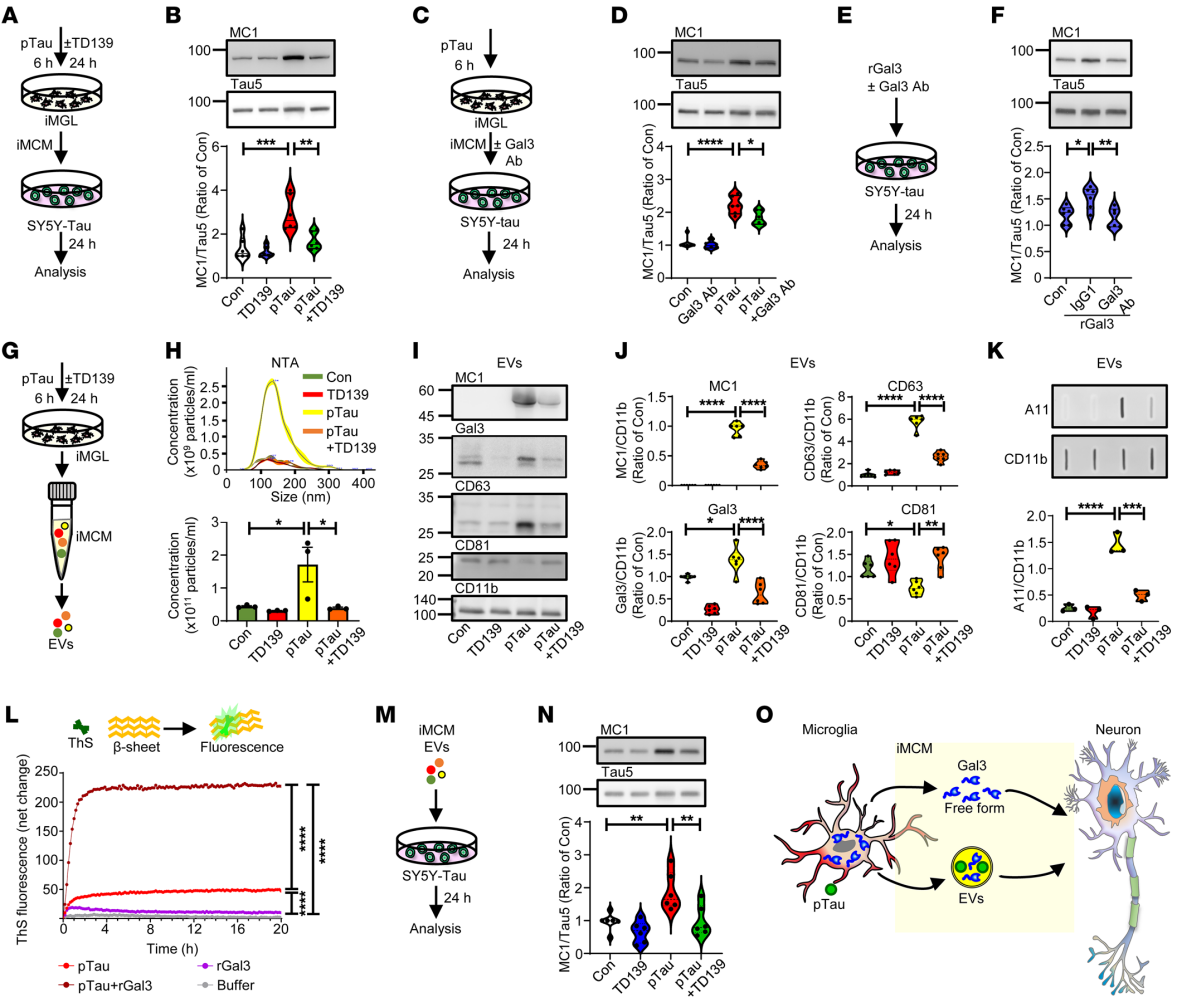
Gal3 in free form and in EVs exacerbates the effect of pathological tau. (**A** and **B**) Immunoblot analysis and quantification of MC1 in SY5Y-tau cells treated with iMCM collected from each group. (**C** and **D**) Immunoblot analysis and quantification of MC1 following pTau iMCM and anti-Gal3 neutralizing antibody coincubation (3 μg/mL). (**E** and **F**) Immunoblot analysis and quantification of MC1 in SY5Y-tau cells treated with recombinant Gal3 (rGal3, 1 μM) and a Gal3 neutralizing antibody, *n* = 6, 3 iMGL lines from 2 independent differentiations for **A**–**F**. (**G** and **H**) EV isolation and NTA quantification of the EV concentration, *n* = 3 iMGL lines. (**I** and **J**) Immunoblot analysis and quantification of MC1, Gal3, CD63, and CD81 in EVs, *n* = 6, 3 iMGL lines from 2 independent differentiations. (**K**) Slot blot analysis of oligomer proteins in EVs based on A11 signals, *n* = 3 iMGL lines. (**L**) Thioflavin-S fluorescence assay to measure in vitro pTau aggregation. Data were analyzed after 20 hours of reaction time. (**M** and **N**) Immunoblot analysis and quantification of MC1 on SY5Y-tau cells treated with EVs (10 μg/mL) from different groups, *n* = 6, 3 iMGL lines from 2 independent differentiations. (**O**) Schematic diagram illustrating how microglial Gal3 may interact with neurons via 2 independent routes. The data in F and L were analyzed by 1-way ANOVA, while those in **B**, **D**, **H**, **J**, **K**, and **N** were analyzed by 2-way ANOVA with Tukey’s test, and violin plots show medians with 25th and 75th percentiles, *P < 0.05, **P < 0.01, ***P < 0.001, ****P < 0.0001.

To evaluate the function of microglial Gal3, iMCM collected from pTau-treated iMGL was added to a neuroblastoma cell line harboring pathogenic tau (SH-SY5Y-eGFP-tau-P301L, SY5Y-tau) (26) (Figure 3A). SY5Y-tau represents a neuronal-like cell line expressing neuronal markers (Supplemental Figure 7, A and B). The treatment of SY5Y-tau cells with iMCM harvested from iMGL treated with pTau, but not pTau plus TD139, resulted in increases in the levels of misfolded tau (MC1-positive) and the activity of GSK-3β (a dominant kinase for tau) in the cells (Figure 3B and Supplemental Figure 7, C and D). Since TD139 is a cell-permeable Gal3 inhibitor, it is likely that TD139 entered iMGL to suppress the activation of iMGL and resulted in iMCM that is less detrimental to SY5Y-tau cells. Notably, the above effect of iMCM was dependent on the presence of iMGL. Without iMGL conditioning, iMGL medium containing pTau alone did not elevate MC1 levels in SY5Y-tau cells (Supplemental Figure 7, E and F).

To investigate the roles of Gal3 detected in iMCM (Figure 2H), SY5Y-tau cells were treated with iMCM collected from pTau-treated iMGL in the absence or presence of a Gal3-neutralizing antibody (Gal3 Ab) that inhibited Gal3 (Figure 3C). Interestingly, the inclusion of Gal3 Ab reduced the misfolded tau level without affecting the activity of GSK-3β in SY5Y-tau cells (Figure 3D and Supplemental Figure 7, G and H), suggesting that Gal3 released from iMGL may enhance the amount of misfolded tau in SY5Y-tau cells. Consistent with this hypothesis, the addition of Atto-565-conjugated recombinant Gal3 (^Atto565^rGal3) to SY5Y-tau cells significantly upregulated the level of misfolded tau (Supplemental Figure 7, I and J). In addition, ^Atto565^rGal3 was found to colocalize with misfolded tau (MC1-positive) in the cells (Supplemental Figure 7, K and L). The inclusion of the Gal3 Ab, but not a control IgG1 antibody, significantly normalized the upregulation of misfolded tau mediated by rGal3 (Figure 3, E and F), suggesting that Gal3 in its free form can enter SY5Y-tau cells and plays a direct role in facilitating the accumulation of misfolded tau.

Microglia have been reported to facilitate the spreading of tau through the release of exosomes containing pathogenic tau (27, 28), we next investigated the role of Gal3 in tau transmission via microglial extracellular vesicles (EVs), including exosomes. The results of nanoparticle tracing analysis (NTA) revealed that pTau markedly enhanced the numbers of EVs released by iMGL, but this effect was not observed in the presence of TD139 (Figure 3, G and H), suggesting that Gal3 may participate in EV biogenesis. Consistent with a recent study showing that Gal3 can be recruited into exosomes (29), treatment with pTau greatly increased the amounts of Gal3 and misfolded tau (MC1-positive) in the EVs of iMGL, which were markedly reduced by TD139 (Figure 3, I and J). Most intriguingly, pTau treatment altered not only the numbers but also the protein contents of EVs. Specifically, the level of CD63 was significantly upregulated, while that of CD81 was markedly downregulated by pTau. CD63 and CD81 are membrane proteins that belong to the tetraspanin family and are commonly associated with EVs, particularly exosomes (30). Analysis of additional EV markers revealed that Alix, but not Tsg101, was also upregulated by the pTau treatment (Supplemental Figure 8, A–C). TD139 treatment normalized the pTau-induced upregulation of CD63 and downregulation of CD81 (Figure 3, I and J), without affecting the upregulation of Alix. Collectively, Gal3 appears to play an important role in regulating EV biogenesis.

To determine whether pathogenic tau and Gal3 are localized inside or outside of the EVs, we conducted a proteinase K resistance assay. Proteinase K treatment effectively cleaved CD11b, which is on the surface of the EVs, while there were no significant reduction on the levels of misfolded tau (MC1-positive) and Gal3 (Supplemental Figure 8, D–F). This suggests that both misfolded tau and Gal3 are mainly located inside the EVs. Most intriguingly, a filter assay analysis revealed that the EVs isolated from iMGLs treated with pTau contained significant amounts of prefibrillar oligomers detected with an A11 antibody (31) (Figure 3K). Treatment with TD139 markedly reduced the levels of these oligomeric proteins, suggesting a Gal3-dependent mechanism. We therefore hypothesized that the coexistence of misfolded tau (MC1-positive) and Gal3 in EVs from pTau-treated iMGL might also facilitate the formation of oligomeric aggregates and contribute to the signals detected by A11.

To assess the possibility that Gal3 directly interacts with pTau and facilitates the accumulation of pathogenic tau, we employed a thioflavin-S fluorescence assay. Full-length rGal3, but not its N-terminal domain (NTD) or C-terminal carbohydrate recognition domain (CRD), greatly increased the aggregation of pTau into β-pleated–sheet structures (Figure 3L and Supplemental Figure 9, A–C). Further, mutations in the aromatic residues (WY/G) of Gal3 (32), which are critical for its liquid-liquid phase separation (LLPS) of Gal3, did not significantly affect its enhancement of pTau aggregation. Conversely, lactose — but not sucrose — which binds with Gal3 in the CRD, reduced the effect of Gal3 on pTau aggregation (Supplemental Figure 9, D–F). Thus, while the CRD is required for this function of Gal3, it is insufficient on its own, as CRD alone did not increase the aggregation of pTau. Further analysis revealed that, during the lag phase of pTau aggregation, the addition of Gal3 significantly increased the aggregation signal. This indicates that Gal3 plays a critical role in facilitating pTau aggregation (Supplemental Figure 9, C and F and Supplemental Table 5). To assess the effect of these microglial EVs on recipient cells, SY5Y-tau cells were exposed to EVs released by iMGLs treated with the specified conditions. EVs isolated from iMGLs treated with pTau markedly augmented the levels of misfolded tau (MC1 positive) in SY5Y-tau cells, while TD139 treatment to iMGL attenuated this pTau-induced effect (Figure 3, M and N).

We next analyzed the levels of Gal3 released by EVs and in its free form. Treatment with pTau elevated Gal3 levels in both the EV-associated and free forms. In all 4 conditions tested, more Gal3 was released from iMGLs in its free form than in EVs (Supplemental Figure 8, G and H). Apart from the coexistence of both Gal3 and pTau in EVs, our study does not specify whether free-form Gal3 also interacts with pTau in other cellular contexts, such as the extracellular spaces between neurons and microglia, or within recipient cells themselves. Collectively, these results indicate that, upon pTau stimulation, microglia release Gal3 in both its free and EV-associated forms, subsequently exacerbating tauopathy (Figure 3O).

### Gal3-associated microglia in THY-Tau22 mice.

**Figure 4:**
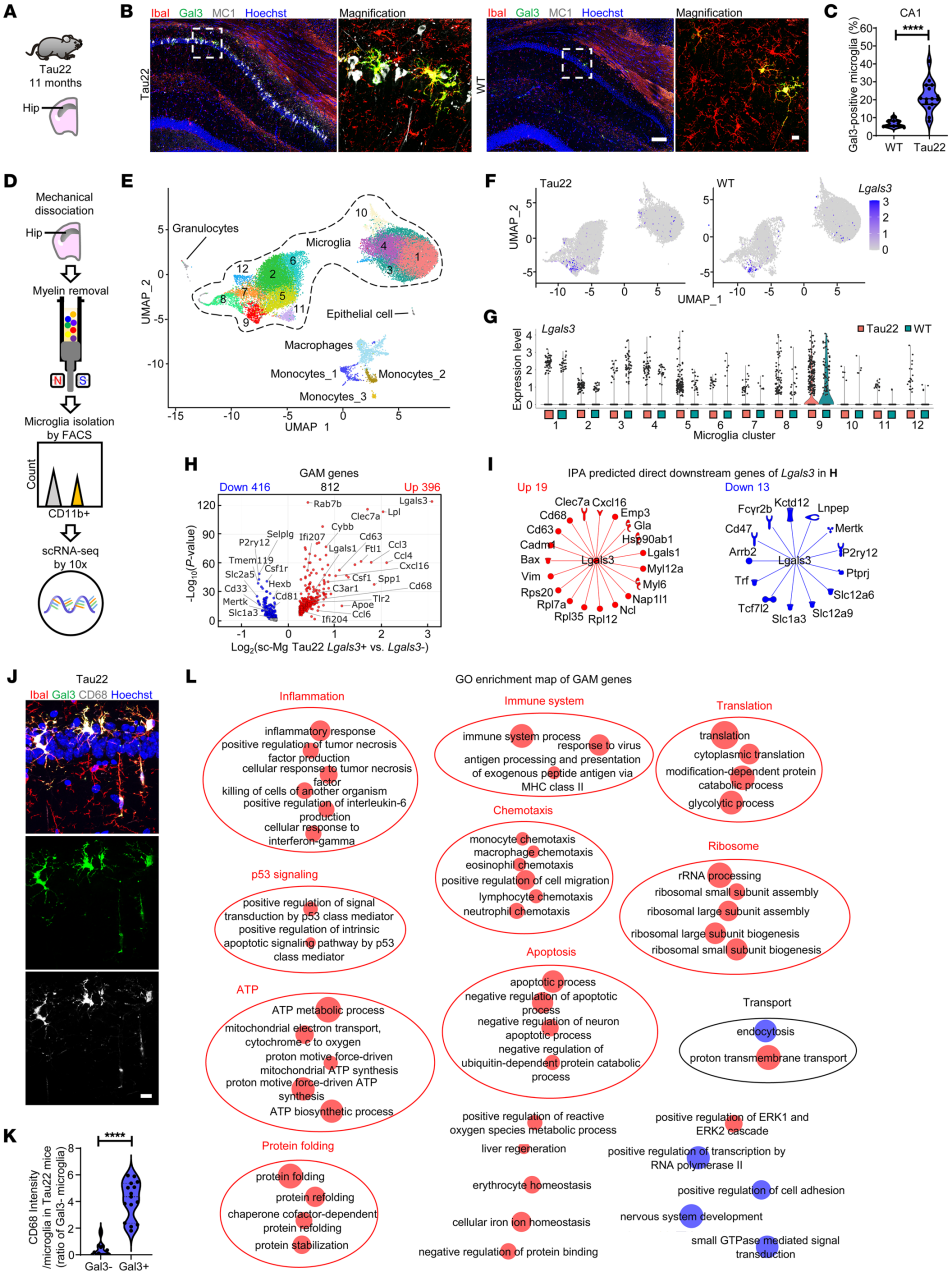
Gal3-associated microglial (GAM) genes. (**A**–**C**) IHC staining and quantification of microglia and Gal3 in the CA1 region of the hippocampus in Tau22 mice, *n* = 3 for WT and *n* = 5 for Tau22, 3 fields per animal. (**D**) Schematic diagram illustrating single-cell microglial isolation. (**E**) UMAP plot showing twelve microglial clusters and nonmicroglial clusters identified from the scRNA-Seq data. (**F**) UMAP shows the expression of *Lgals3* in all microglia. (**G**) Violin plots show the expression of *Lgals3* in microglial clusters. (**H**) Volcano plot shows the GAM genes identified in Tau22 mice. (**I**) IPA prediction of direct downstream Gal3-regulated genes. (**J**–**K**) IHC staining and quantification of microglial activation marker (CD68) on microglia with or without Gal3 expression in Tau22 mice, *n* = 5 mice in each group, 3 fields per animal. (**L**) Enrichment map of GAM genes. Clusters were defined based on the REVIGO visualized by the WordCloud app in Cytoscape. Scale bars: 10 μm (**B** [right] and **J**); 100 μm (**B** [left]),. Data were analyzed with the 2-tailed unpaired *t* test. Violin plots show the median with the 25th and 75th percentiles, ****P < 0.0001.

In line with our observations in human AD and FTLD brains, we detected an upregulation of Gal3 in microglia located adjacent to neurons containing misfolded tau (MC1-positive) and aggregated tau (AT100-positive) in the CA1 region of THY-Tau22 mice (Tau22; Figure 4, A–C and Supplemental Figure 10A). Tau22 is an animal model of tauopathy. It is based on the overexpression of a human tau transgene carrying mutations associated with FTLD, and it progressively develops hippocampal tau pathology (33). Additionally, we found that exogenously added recombinant Gal3 labeled with Atto565 (designated ^Atto565^rGal3) preferentially bound to the MC1-positive neurons of hippocampal slices of Tau22 mice over those of WT mice (Supplemental Figure 10B). These findings suggest a potential involvement of extracellular Gal3 in mediating abnormal interactions between microglia and neurons in the context of tauopathy.

To investigate the transcriptomic profile of Gal3-positive microglia by single-cell RNA-Seq (scRNA-Seq), hippocampal tissues of WT and Tau22 mice (12.5 months old, *n* = 8 in each group) were harvested and subjected to mechanical dissociation. After myelin removal, the cell suspensions were stained with an anti-CD11b antibody — a myeloid cell marker — and isolated by FACS. The CD11b-sorted cells were subjected to scRNA-Seq by using the 10× Genomics platform (Figure 4D). We conducted quality control (Supplemental Figure 11, A–D), principal component analysis, and dimensionality reduction using uniform manifold approximation and projection (UMAP) (Supplemental Figure 11, E and F). The isolated cells, mostly microglia, were assigned to each cluster based on the expression of established markers from the PanglaoDB database (34). In total, we identified 12 distinctive clusters of microglia, 3 clusters of monocytes, and 1 cluster of macrophages, granulocytes and epithelial cells (Figure 4E). Compared with WT mice, Tau22 mice had more microglia in 7 Clusters (1, 2, 5, 6, 9, 11, 12) and fewer microglia in 2 Clusters (3, 10). 3 Clusters (3, 7, 8) had no major change in their numbers (i.e., the change in microglia population was less than 1%; Supplemental Figure 11G). The DEGs (log_2_-fold–change threshold of at least 0.25) identified by Seurat 4.0 were subjected to GO analysis by using DAVID Bioinformatics Resources (35, 36) to highlight the major biological processes and marker genes associated with each microglial cluster (Supplemental Figures 12–14). Interestingly, 6 of the Clusters (2, 5, 6, 9, 11, 12) with increased cell numbers are enriched with translational processes. For pseudotime analysis to identify the longest trajectory of cell clusters based on the difference on gene profile from their origin, Cluster 1 and Cluster 2 with the highest cell number were appointed as the origins. The longest pseudotime distance for Cluster 1 and Cluster 2 are Clusters 10 and 9, respectively (Supplemental Figure 11H). Gal3 (*Lgals3* in mice)-associated microglia (GAM) were enriched in Cluster 9 and could also be observed in several other clusters to a lesser extent (Figure 4, F and G).

We next investigated the involvement of these microglial clusters in the pathway associated with EVs, such as exosomes, multivesicular bodies, and endosomal pathways. Interestingly, we found that Clusters 4 and 9 were enriched with cellular components related to exosomes (Supplemental Figure 15A). Among the exosome components, CD63 and CD9 were previously identified as DAM genes (23) and notably exhibited high expression levels in Cluster 9 (Supplemental Figure 15B). The finding prompted us to compare microglial clusters with DAM genes using hierarchical clustering analysis. This revealed that Cluster 9 exhibited the closest similarity to DAM, followed by Clusters 7 and 8 (Supplemental Figure 15C). Additionally, when comparing microglial clusters with microglia of neurodegenerative diseases (MGnD) genes (18), we found that Cluster 9 also displayed highest similarity to MGnD, followed by Clusters 8 and 12 (Supplemental Figure 15D). These findings suggest that the *Lgals3-*enriched Cluster 9 may play a pivotal role in pathways associated with EVs as evidenced by the iMGL study, and may potentially contribute to the intricate interplay between microglial activity and tau transmission.

Because not all microglia express *Lgals3*, we aimed to specifically characterize the *Lgals3*-positive and *Lgals3*-negative microglia. We define *Lgals3*-positive microglia as cells with *Lgals3* expression levels greater than or equal to 1 average log Unique Molecular Identifier (UMI) count, which serves as a reference point. Compared with *Lgals3*-negative microglia in Tau22 mice, which had a zero average log UMI count, *Lgals3*-positive cells exhibited 812 DEGs, designated as GAM genes (Figure 4H, Supplemental Figure 16A, and Supplemental Table 6). Consistent with the findings in human iMGLs (Figure 3I), we found that Cd63 and Cd81 were both GAM genes in the microglia isolated from Tau22 mice, and were upregulated and downregulated, respectively (Figure 4H). Moreover, GO analysis revealed enrichment of GAM genes in cellular components such as exosome, multivesicular body, and late endosome (Supplemental Figure 16, B and C), further supporting the role of Gal3 in the regulation of EV-related pathways.

Further examination via Ingenuity Pathway Analysis (IPA) showed that 19 of the 396 upregulated GAM genes and 13 of the 416 downregulated GAM genes were direct downstream target genes of *Lgals3* (Figure 4, H and I). Consistent with this prediction, immunofluorescence staining demonstrated that CD68, a gene directly downstream of Gal3 (Figure 4I) was expressed at a higher level in Gal3-positive microglia compared with that of the Gal3-negative microglia, in Tau22 mice (Figure 4, J and K). Further analyses of GAM genes using the Reduce Visualize Gene Ontology (REVIGO) tool (37) highlighted the activation of translation and ribosome activities, inflammation, and the immune system, followed by the processes of ATP production, apoptosis, chemotaxis, protein folding, and p53 signaling (Figure 4L). Interestingly, the 812 GAM genes shared 172 genes with the AD DAM genes (23) (Supplemental Figure 17A), including upregulated microglial activation genes (such as *Clec7a*, *Cd68*, *Csf1*, *Apoe*, and *Cybb*) and downregulated microglial homeostatic genes (such as *P2ry12*, *TMEM119*, *Csf1r*, *Hexb,* and *Slc2a5*). Collectively, GAM is a subset of microglia with several features similar to those of DAM (e.g., the enhanced inflammatory responses and protein translation processes) and some unique only to GAM (including the protein folding process, energy metabolism, transcription, and specific translation processes) (Figure 4L and Supplemental Figure 17, B–F). Additionally, we conducted a comparative analysis between GAM in Tau22 mice and the DEGs in pTau-induced iMGLs, as well as the effects of Gal3 inhibition with TD139, to explore their potential relevance for future cross-species studies. Among the identified conservation of canonical pathways, pathways such as hepatic fibrosis signaling, Rho family GTPases, neuroinflammation, integrin, and IL8 signaling, were suppressed in the presence of TD139 (Supplemental Figure 18, A and B).

### Loss of Gal3 protects against tauopathy.

**Figure 5:**
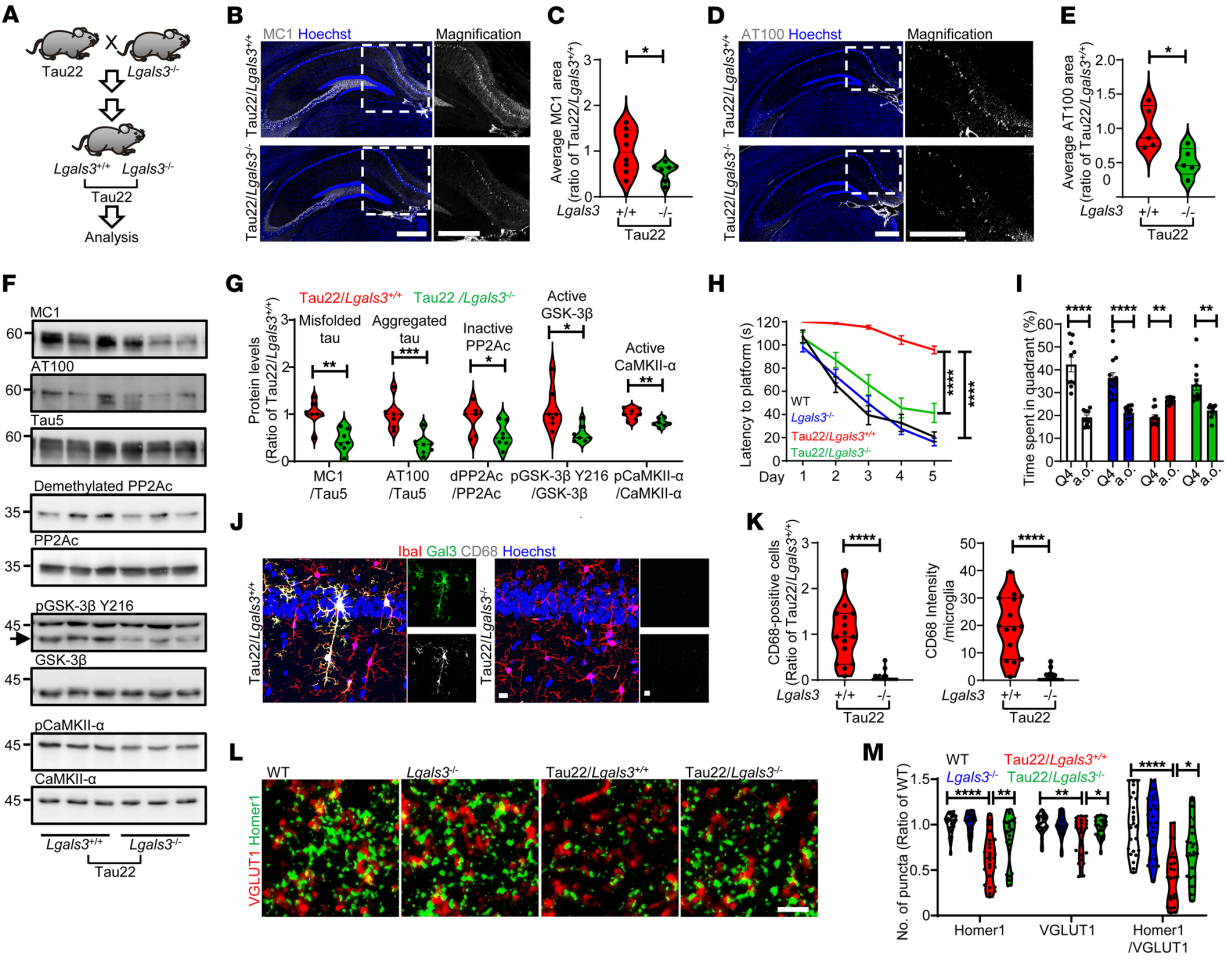
Loss of Gal3 rescues tauopathy in THY-Tau22 mice. (**A**) Schematic diagram illustrating the study design to identify the roles of Gal3 in Tau22 mice. IHC staining and quantification for (**B** and **C**) MC1 and (**D** and **E**) AT100 in mouse hippocampi, MC1: *n* = 8 for Tau22/*Lgals3*^+/+^, *n* = 7 for Tau22/*Lgals3*^–/–^; AT100: *n* = 5 mice. Each dot represents the average value of each animal. (**F**) Immunoblot analysis of sarkosyl soluble mouse hippocampi (11 months) stained for MC1, AT100, Tau5, demethylated PP2A subunit C (inactive PP2Ac), PP2Ac, pGSK-3β Y216, GSK-3β, pCaMKII-α, and CaMKII-α. (**G**) Quantification of the data in **F**, *n* = 7 mice. (**H**) Quantification of the time to platform in the 5-day training section of the Morris water maze, *n* = 13 for WT, *n* = 17 for *Lgals3*^–/–^, *n* = 12 for Tau22/*Lgals3*^+/+^, *n* = 16 for Tau22/*Lgals3*^–/–^. (**I**), Quantification of the time spent in quadrant 4 (Q4, the quadrant with the hidden platform during the training section) versus all other quadrants (a.o., average of 3 other quadrants) on probe trial Day 8. Data are shown as the mean ± SEM. (**J** and **K**) IHC staining and quantification of CD68 in Tau22/*Lgals3*^–/–^ and Tau22/*Lgals3*^+/+^ mice, *n* = 5 mice, 3 fields per animal. (**L**) IHC staining of Homer 1 and VGLUT1 in the CA1 region of Tau22/*Lgals3*^–/–^ and control mice. (**M**) Quantification of the staining in L, *n* = 8 mice, 3 fields per animal. Scale bars: 500 μm (**B** and **D**), 10 μm (**J**), 2 μm (**L**). Data in **H** and **M** were analyzed by 2-way ANOVA with Tukey’s test. Other data were analyzed with a 2-tailed unpaired *t* test, and all violin plots show the median with the 25th and 75th percentiles. *P < 0.05, **P < 0.01, ***P < 0.001, ****P < 0.0001.

To assess the importance of Gal3 in tauopathies in vivo, we crossed Tau22 mice with Gal3 knockout mice (Tau22/*Lgals3*^–/–^, Figure 5A). The knockout of Gal3 reduced the levels of misfolded tau (MC1-positive), aggregated tau (AT100-positive), and phosphorylated tau (AT8-positive) in the hippocampal CA1 region of Tau22, as assessed by immunofluorescence (Figure 5, B–E and Supplemental Figure 19, A and B) and Western blot analyses (Figure 5, F and G). We next investigated the major phosphatase, PP2A, and kinases that regulate the abnormal hyperphosphorylation of tau (38). Compared with Tau22/*Lgals3^+/+^* mice, Tau22/*Lgals3*^–/–^ mice exhibited reduced levels of the inactive/demethylated form of PP2A and decreased kinase activities of GSK-3β and CaMKII-α (Figure 5, F and G). Such alterations in PP2A and kinase activities may result in the reduction of tau phosphorylation. Importantly, the loss of Gal3 also prevented the learning and memory deficits present in Tau22/*Lgals3^+/+^* mice (33) to a great extent, as assessed by the Morris water maze test (Figure 5, H and I). Consistent with the GAM gene analysis (Figure 4, H and I), the number and level of CD68-positive microglia in Tau22/*Lgals3*^–/–^ mice were indeed lower than those in Tau22/*Lgals3^+/+^* mice (Figure 5, J and K), suggesting a key role of Gal3 in microglial activation. Given that synaptic loss is a feature of tauopathy that is also presented in Tau22 mice (33), we performed immunofluorescence staining of synapses at the CA1 region using VGLUT1 and Homer1 as presynaptic and postsynaptic markers, respectively. Our data showed that Gal3 knockout rescued the number of synapses assessed by the colocalization of VGLUT1 and Homer1 (Figure 5, L and M and Supplemental Figure 20, A and B). This finding suggests that GAM mediates the loss of synapses in Tau22 mice.

**Figure 6:**
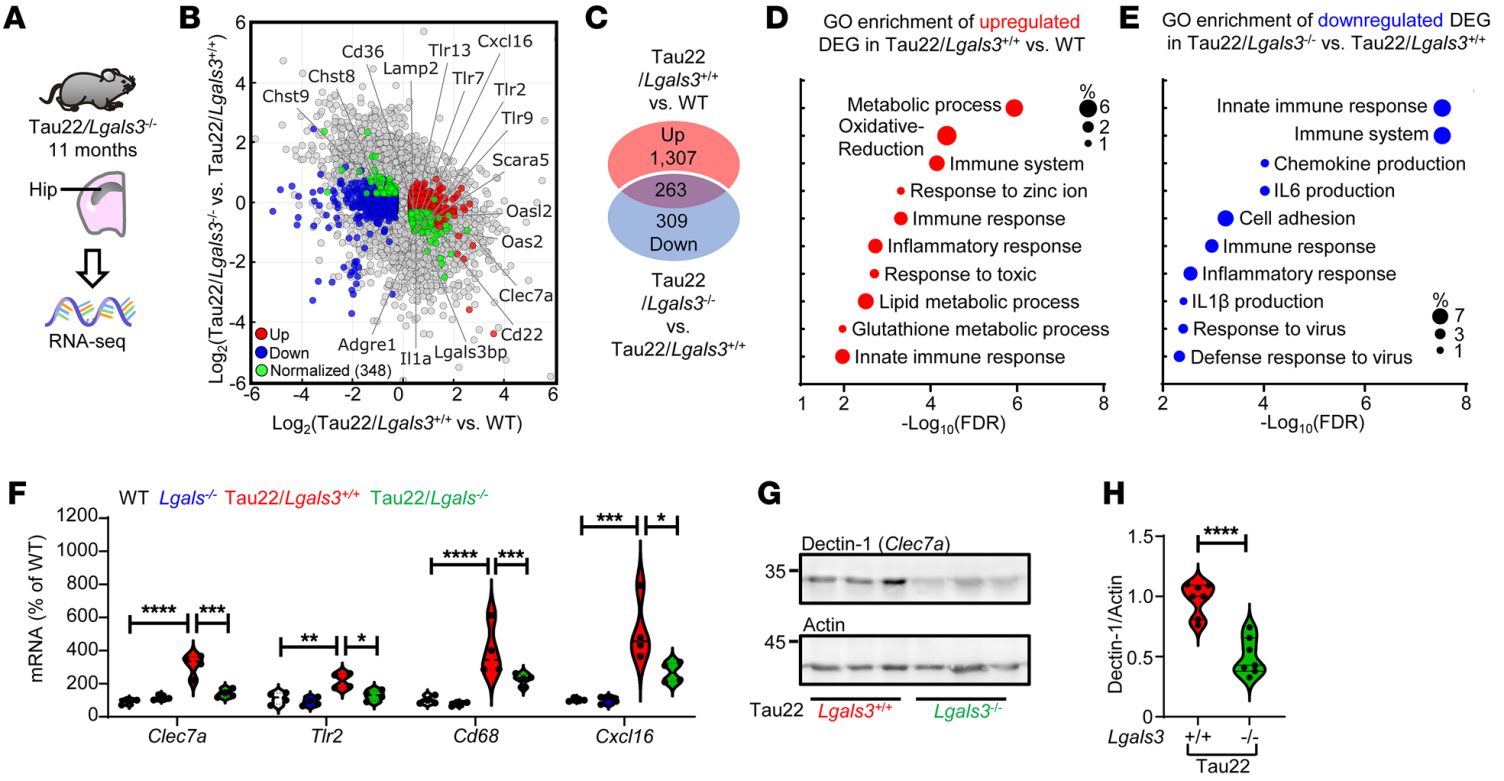
Deletion of Gal3 ameliorates disease-associated pathways in tauopathy. (**A**) RNA isolated from the hippocampi of Tau22/*Lgals3*^–/–^ and control mice was subjected to RNA-Seq. (**B**) Scatterplot showing DEGs identified between Tau22/*Lgals3*^+/+^ and WT mice and between Tau22/*Lgals3*^–/–^ and Tau22/*Lgals3*^+/+^ mice. Red and blue dots indicate upregulated and downregulated DEGs, respectively, and green dots indicate genes that are normalized in Tau22/*Lgals3*^–/–^ mice compared with Tau22/*Lgals3*^+/+^ mice. (**C**) Venn diagrams show the numbers of normalized genes in each group. (**D** and **E**) GO enrichment analysis of upregulated DEGs in Tau22/*Lgals3*^+/+^ versus WT mice, and downregulated DEGs in Tau22/*Lgals3*^–/–^ versus Tau22/*Lgals3*^+/+^ mice. (**F**) qPCR validation of selected normalized DEGs, *n* = 4 mice. Data were analyzed by 2-way ANOVA with Tukey’s test. (**G** and **H**) Immunoblot detection and quantification of dectin-1 (a.k.a *Clec7a*) in the hippocampi of Tau22/*Lgals3*^–/–^ versus Tau22/*Lgals3*^+/+^ mice, *n* = 7 mice. Data were analyzed with the 2-tailed unpaired *t* test, and violin plots show medians with 25th and 75th percentiles, *P < 0.05, **P < 0.01, ***P < 0.001, ****P < 0.0001.

To further delineate the protective role of Gal3 depletion in tauopathy, we analyzed the gene expression profiles of the hippocampi of Tau22/*Lgals3*^–/–^ mice and corresponding controls using bulk RNA-Seq. In total, 3,770 DEGs were identified between Tau22/*Lgals3^+/+^* and WT mice, and 868 DEGs were identified between Tau22/*Lgals3*^–/–^ and Tau22/*Lgals3^+/+^* mice (Supplemental Figure 21, A–D). In particular, the knockout of Gal3 normalized 348 DEG genes between Tau22/*Lgals3^+/+^* and WT mice (Figure 6, A and B and Supplemental Table 7). Further GO enrichment analysis revealed that the upregulated DEGs of Tau22/*Lgals3^+/+^* versus WT mice were enriched in multiple processes, including metabolic processes, oxidative reduction processes, and immune system processes (Figure 6, C and D). Importantly, the downregulated DEGs by *Lgals3* deletion within the context of Tau22 were primarily enriched in immune responses and the production of cytokines and chemokines (Figure 6E). Conversely, the downregulated DEGs in Tau22/*Lgals3*^–/–^ versus Tau22/*Lgals3^+/+^* mice were enriched in processes including nervous system development, protein phosphorylation, synapse assembly, and learning (Supplemental Figure 21, E and F). No specific enriched processes were identified for the upregulated DEGs in Tau22/*Lgals3*^–/–^ versus Tau22/*Lgals3^+/+^* mice. These findings are consistent with what were observed in human iMGLs, confirming that Gal3 plays a principal role in governing the microglia-mediated immune response in tauopathy.

We next categorized these DEGs by their enriched cell type based on the Tabula Muris Consortium database (39) (Supplemental Figure 21D), and, as predicted, we found that the largest population of DEGs identified between Tau22/*Lgals3*^–/–^ and Tau22/*Lgals3^+/+^* mice was enriched in microglia (21.3%; Supplemental Figure 21D). The expression of 4 microglia-enriched DEGs (i.e., *Clec7a*, *Tlr2*, *Cd68,* and *Cxcl16*), which were also identified as GAM genes by scRNA-Seq analyses (Figure 4H), was validated by RT–PCR (Figure 6F). Accordingly, the expression of the Dectin-1 protein, encoded by *Clec7a*, a direct downstream target of Gal3 (Figure 4I), was significantly downregulated by the deletion of Gal3 in Tau22 mice (Figure 6, G and H). Nonetheless, although the levels of *Apoe* and *Trem2* were upregulated in Tau22/*Lgals3^+/+^* mice, the deletion of Gal3 did not reverse their expression (Supplemental Figure 21, G and H). Importantly, when compared with the Gal3-enriched Cluster 9 microglia from the scRNA-Seq analysis (Figure 4G), bulk RNA-Seq analysis showed that 19 upregulated genes and 1 downregulated gene exhibited reversed expression levels in Tau22/*Lgals3*^–/–^ mice (Supplemental Figure 21, I and J).

Interestingly, the removal of Gal3 also ameliorated the increase in the number of GFAP-positive astrocytes in Tau22 mice (Supplemental Figure 22, A and B). This observation supports our hypothesis that Gal3 plays an important role in microglia activation because previous studies had demonstrated that activated microglia may secrete immune mediators (such as IL1α, TNFα, and C1q) that contribute to the transformation of neurotoxic reactive astrocytes (A1 astrocytes) (40). Additionally, astrogliosis has been reported in tauopathy (41). Transcriptomic analysis further revealed that the levels of several genes associated with A1 astrocytes (including *Fbln5*, *Ugt1a1*, *Gbp2*, *C3,* and *Srgn*) were reduced in Tau22/Lgals3^–/–^ mice (Supplemental Figure 22C).

### Gal3 mediates Aβ-induced tau pathology.

**Figure 7:**
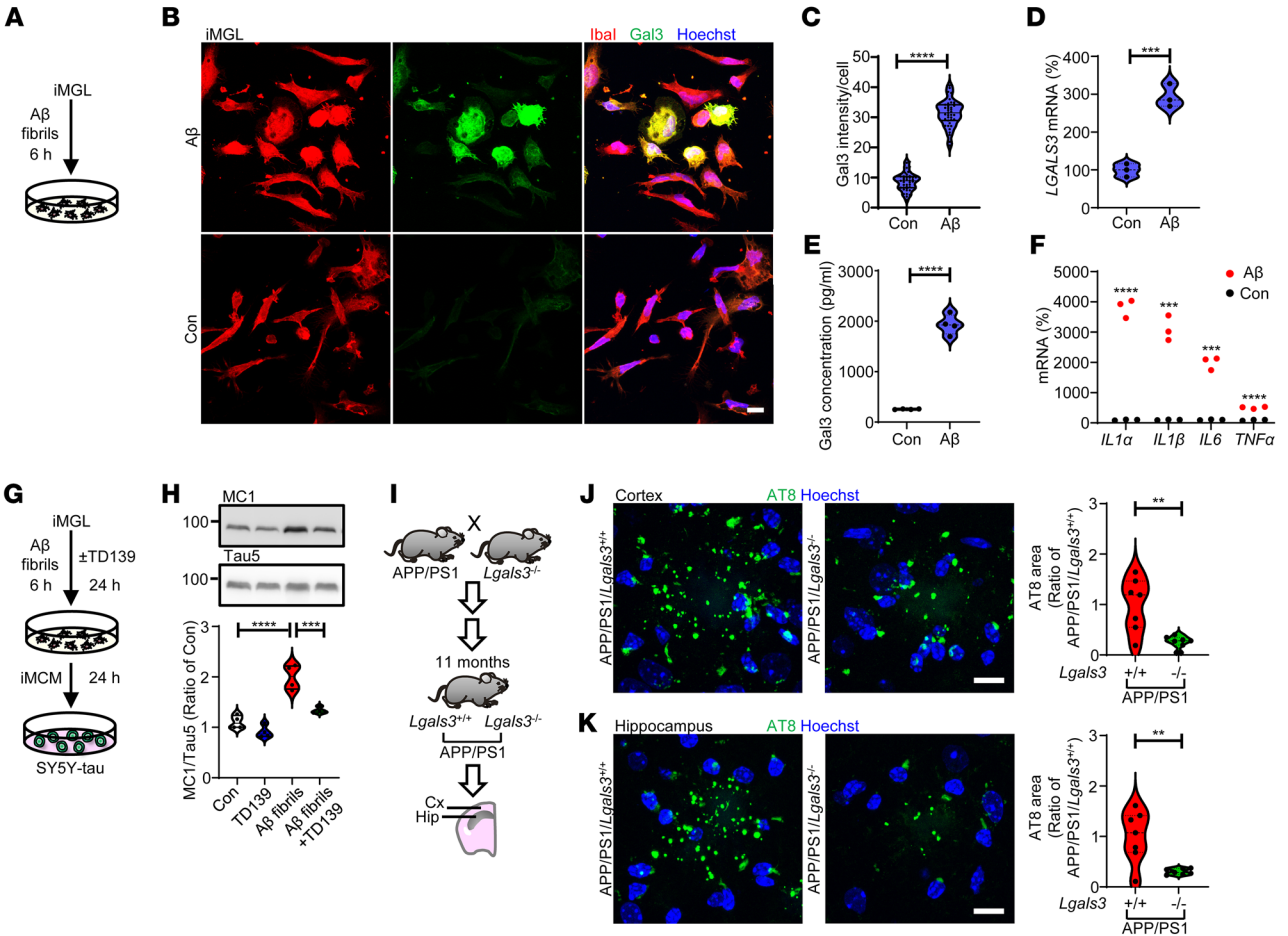
Gal3 exacerbates amyloid-β–induced tauopathy. (**A**–**C**) Immunofluorescence staining of Gal3 and IbaI in iMGL treated with fibrillar amyloid-β (1 μM) for 6 hours, *n* = 3 iMGL lines, 3 coverslips per iMGL line, 3–4 fields per coverslip. qPCR analysis of (**D**) *LGALS3* and (**F**) proinflammatory genes in iMGL, *n* = 3 iMGL lines. (**E**) Gal3 ELISA of conditioned medium collected from iMGL, *n* = 3 iMGL lines, 1 line with 2 independent differentiations. (**G** and **H**) Immunoblot analysis and quantification of MC1 in cells treated with fibrillar amyloid-β iMCM and TD139 (10 μM, Gal3 inhibition), *n* = 3 iMGL lines, each line with 2 independent differentiations. (**I**–**K**), IHC staining and quantification of pTau (AT8) in 11-month-old APP/PS1/*Lgals3*^–/–^ versus APP/PS1/*Lgals3*^+/+^ mice, *n* = 7 mice. Scale bar: 10 μm. The data in **H** were analyzed by 2-way ANOVA with Tukey’s test, and those in **C**–**F**, **J**, and **K** were analyzed with the 2-tailed unpaired *t* test, and violin plots show medians with 25th and 75th percentiles, **P < 0.01, ***P < 0.001, ****P < 0.0001.

Ample evidence suggests that amyloid plaque formation precedes tau pathology in AD. Dystrophic neurites with phosphorylated tau (AT8-positive) have been detected in mouse models of amyloid pathology, such as APP/PS1 and APP-knockin mice (42, 43), but the involvement of microglia remains largely unclear. Here, we showed that the treatment of iMGL with fibrillar Aβ for 6 hours significantly upregulated Gal3 (Figure 7, A–D). Additionally, we conducted a morphological analysis of iMGL and assessed the expression levels of Gal3. While a portion of Gal3-expressing iMGL appeared to display a round morphology, we found no significant correlation between Gal3 expression and round-shaped morphology (Supplemental Figure 23, A–C). Conversely, we observed a positive correlation between Gal3 expression and the size of iMGL in the presence of fibrillar Aβ (Supplemental Figure 23D). Furthermore, fibrillar Aβ also triggered the secretion of Gal3 (Figure 7E). Additionally, we observed the upregulation of proinflammatory genes as an early response to fibrillar Aβ by iMGL (Figure 7F). To investigate the effects of Gal3 on Aβ-induced tau pathology, we collected iMCM from iMGL exposed to fibrillar Aβ with or without TD139. iMCM from fibrillar Aβ-exposed cells induced a higher level of misfolded tau (MC1-positive) in SY5Y-tau cells, which was prevented by inhibiting Gal3 in iMGL using TD139 (Figure 7, G and H). We next crossed APP/PS1 mice with Gal3 knockout mice and found that the levels of phosphorylated tau present in the dystrophic neurites of the cortex and hippocampus of APP/PS1/*Lgals3*^–/–^ mice were lower than those of APP/PS1/*Lgals3^+/+^* mice (11 months old, symptomatic stage; Figure 7, I–K). Moreover, the level of Aβ plaques were also lower in APP/PS1/*Lgals3*^–/–^ mice (Supplemental Figure 24), supporting the conclusion that Gal3-positive microglia are essential for the Aβ-tau axis in AD.

## Discussion

**Figure 8:**
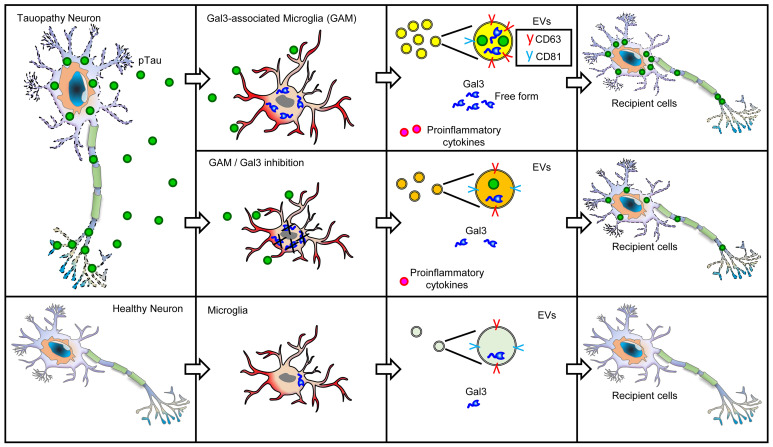
Schematic diagram illustrating the roles of Gal3 in microglia-mediated tau transmission and fibrillation.

Increasing evidence shows that pathological tau is transmitted and propagated between neurons (44–46). The secreted tau in its physiological state is dephosphorylated, while the pathological tau is phosphorylated and misfolded (47, 48). Nonetheless, both pTau and nonphosphorylated tau have been found in the CSF of patients with AD, indicating that both forms of tau can be secreted (49, 50). As the primary phagocytic cells in the brain, microglia play a major role in the clearance of extracellular tau. An early study demonstrated that microglia clear pTau slightly faster than nonphosphorylated tau (51). However, whether microglia exhibit a preference for taking up pTau remains unclear. Here, we present evidence demonstrating distinctive effect of pTau in recapitulating the dysregulation of microglial genes that have been observed in patients with AD (Figure 1, H and I). Interestingly, such dysregulation was not observed under experimental conditions involving nonphosphorylated tau and LPS. Our findings collectively suggest that Gal3-associated microglia facilitate the transmission and aggregation of misfolded tau to recipient cells (Figure 8). When encountering pathological tau, microglia become active and release Gal3 into the extracellular space either directly or via EVs (Figure 3O). Under the tested conditions, we found that Gal3 directly facilitated the aggregation of pTau into β-pleated–sheet structures (Figure 3L). This interaction between pTau and Gal3 may occur in EVs and/or the extracellular space between microglia and neurons. It is plausible that Gal3 may interact with pTau and serve as an opsonin to enhance the uptake of pTau by other brain cells (52, 53). Most importantly, we showed that Gal3 released by activated microglia in either free form or EV-associated form facilitated the accumulation of misfolded tau in SY5Y-tau cells (Figure 3, D and N). Moreover, the deletion of Gal3 markedly reduced the extent of tauopathy in THY-Tau22 mice (Figure 5, A–E). Taken together, the results indicate that the upregulation of Gal3 in microglia plays a critical role in the propagation of tauopathy.

Another function of Gal3 reported in the present study is that Gal3 may contribute to the altered release and properties of EVs derived from pTau-treated microglia (Figure 3, G–J). This is of great interest because EVs are a route of tau transmission (45). A previous study demonstrated that the deletion of Trem2 enhances the transmission of tau via exosomes (27). Our findings indicate that pTau treatment significantly reduces the level of *TREM2* in iMGL, and these iMGL did release more EVs. Intriguingly, Gal3 inhibition suppressed the abnormal release of EVs triggered by pTau, without affecting the levels of *TREM2* (Supplemental Figure 4K). This suggests that Gal3 might act either as a key mediator downstream of TREM2 or within an independent pathway parallel to TREM2 in regulating the EV pathway. Consistent with the results of our IPA of GAM genes indicating that CD63 (a marker of EVs resulting from endosomes (30)) may be a direct downstream target of Gal3 (Figure 4, H and I), the level of CD63 in EVs derived from pTau-treated iMGL was markedly elevated (Figure 3, I and J). Furthermore, scRNA-Seq analysis showed enriched levels of EV-associated components, including exosomes, late endosomes, and multivesicular bodies, in GAM (Supplemental Figure 16, B and C).

Because we employed a low concentration of pTau (50 nM) to induce Gal3 upregulation in iMGLs and microglial activation in the present study, immunofluorescence staining could not reliably detect pTau signals in iMGLs after a 6-hour treatment. It is possible that at this low concentration, any pTau taken up by iMGLs has been either secreted out or digested within the 6-hour treatment period. Interestingly, although we could not observe pTau in iMGL cells using the immunofluorescence staining method, we successfully detected pTau in the EVs isolated from pTau-treated iMGL, as shown in Figure 3I. This suggests that iMGL may secrete pTau through EVs. These observations together suggested that microglia enhanced the release and production of EVs in a Gal3-dependent manner when encountering pTau. Because Gal3 regulates the intracellular trafficking of cellular protein(s) by interacting with Alix (an accessory protein associated with the endosomal sorting complex required for transport [ESCRT] machinery) (54) and Alix plays a key role in apical exosome release (55), it is plausible that cytosolic Gal3 may alter the transport of different cellular proteins to their destinations, including exosomes and EVs, by binding to Alix in pTau-treated microglia. Further investigation is warranted to delineate the underlying mechanism.

The function of Gal3 in EV biogenesis has not been clearly defined. A recent study showed that HIV infection significantly increases the level of Gal3 in exosomes derived from DCs and that Gal3 mediates HIV transmission to T cells by DC exosomes (56). Our data showed that the presence of Gal3 in the same EV compartment as pTau facilitated the formation of pathogenic tau oligomers and increased the accumulation of misfolded tau in the recipient neuronal-like cells (Figure 3, I–N). Given that TD139 is membrane-permeable, it may inhibit not only intracellular and extracellular Gal3, but also EV Gal3. The inhibition of Gal3 by TD139 in tauopathy not only prevented the EV-mediated spread of misfolded tau by microglia but also reduced the amount of EV Gal3, which might target neighboring neurons to promote the accumulation of pathogenic tau.

It is a long-held view that activated microglia and innate immune factors contribute significantly to the pathogenesis of AD and tauopathy. Although microglial depletion has been shown to reduce AD pathology (57), microglial activation is complex and can produce both beneficial and detrimental effects on neuronal functions. In the present study, we identified a group of Gal3-associated microglia (GAM) in an animal model of tauopathy (THY-Tau22) that facilitate the progression of tauopathy (Figure 4). Notably, these GAM were located in close proximity to neurons containing misfolded tau in the hippocampi of Tau22 mice (Figure 4B). It appears that microglia respond to signals (e.g., released pathogenic tau) from pTau-containing neurons by upregulating Gal3 and activating microglia. Accordingly, the addition of pTau to iMGL evoked Gal3 upregulation and the production of proinflammatory cytokines (Figure 2, G and H). In addition, GAM may also increase the phosphorylation of tau via the secretion of proinflammatory factors that regulate the activities of kinases and phosphatases in neurons, as reported elsewhere (38). Collectively, the results indicated that the genetic deletion of Gal3 in Tau22 mice ameliorated major disease-related symptoms (Figure 5), supporting the importance of GAM in tauopathy.

The results of the present study and many other reports suggest that Gal3 plays a critical role in a wide variety of neurodegenerative diseases, including AD, Huntington’s disease, and Parkinson’s disease (1, 15, 16, 58). It is likely that Gal3 mediates a conserved activation program and a stimulus-specific program in microglia while encountering various stimuli. A comparison of GAM genes and AD DAM genes revealed 172 common genes between these groups (Supplemental Figure 17, A and B). Both the GAM and DAM genes highlighted translation, ribosome assembly and biogenesis, TNF production, and the inflammatory response, mitochondrial electron transport, and antigen processing and presentation (Supplemental Figure 17D) (23). Interestingly, GAM genes were also enriched in pathways related to apoptosis, protein folding, ATP metabolic processes and IFN-γ signaling (Supplemental Figure 17E), indicating that the GAM genes reported here may regulate a subset of microglial activities specific to pTau. Notably, abnormal activation of the ribosomal pathway has also been reported in amyloid plaque–associated microglia (59), which are activated with Gal3 signals (1, 19). These findings support our hypothesis that microglial Gal3 is an important regulator of the promotion of tauopathy by Aβ (Figure 7).

In summary, our findings demonstrate that Gal3 plays a critical upstream role in tauopathy by controlling microglial activation and the propagation of tauopathy. Herein, we demonstrated that the exposure of microglia to pathological tau from neurons led to the immediate upregulation of Gal3 in a subset of microglia, GAM, and a network of genes initiating the activation of microglia, which subsequently caused synaptic loss and neurodegeneration in tauopathy. Most intriguingly, microglia were shown to release Gal3 in both its free and EV-associated forms, facilitating the accumulation of misfolded tau in recipient neuronal cells. Direct interaction between Gal3 and misfolded tau may occur in EVs and/or the extracellular space and could greatly enhance the formation of pathogenic pTau. The removal of Gal3 markedly reduced tau pathology in mouse models of tauopathy and AD. The lack of a Gal3-dependent TREM2/APOE response indicates that Gal3 affects EV dynamics and tauopathy without directly intersecting with the TREM2/APOE signaling pathways. By focusing on Gal3 inhibition, therapeutic strategies could potentially modulate EV dynamics and microglial activation without altering the TREM2/APOE pathway, which could be advantageous in cases where modulation of TREM2/APOE is neither desirable nor effective.

## Methods

Specific details on the research materials and portions of the experimental protocols can be found in the Supplemental Methods.

### Animals.

Tau22 mice (B6. Cg-Tg(Thy1-MAPT)22Schd) express mutated human tau (G272V and P301S) associated with FTLD (1N4R tau) driven by a neuron-specific promoter (Thy1.2), and were maintained in the C57BL/6J background (33). APP/PS1 mice (B6C3-Tg(APPswe,PSEN1dE9)85Dbo/Mmjax) were originally obtained from The Jackson Laboratory. Gal3 knockout mice (*Lgals3^–/–^*) in a C57BL/6J background (60) were kindly provided by Fu-Tong Liu from the Institute of Biomedical Sciences, Academia Sinica. Tau22/*Lgals3^+/+^* and APP/PS1/*Lgals3^+/+^* mice were initially crossed with Gal3 knockout mice (WT/*Lgals3^–/–^*). The resultant offspring were then crossed to obtain Tau22/*Lgals3^–/–^* and APP/PS1/*Lgals3^–/–^* mice. Mice were housed at a density of no more than 5 per cage in individually ventilated cages, with water and a chow diet (LabDiet), under a 12:12 hour light/dark cycle at the Institute of Biomedical Sciences Animal Care Facility (Taipei, Taiwan).

### Cells.

The neuronal-like cell line SH-SY5Y-eGFP-tau-P301L (SY5Y-tau) (26) was a gift from Yung-Feng Liao at the Institute of Cellular and Organismic Biology, Academia Sinica. SY5Y-tau cells were grown in DMEM (HyClone) supplemented with 10% heat-inactivated FBS, 5 μg/mL blasticidin (Gibco) and 200 μg/mL hygromycin (Thermo Fisher Scientific) at 37 °C in a humidified 5% CO_2_-containing atmosphere. All SY5Y-tau cells were induced to express tau for subsequent experiments with 1 μg/mL doxycycline hyclate (Santa Cruz).

### Human tissue samples.

Postmortem human cortical and hippocampal specimens from 4 normal individuals, 6 patients with FTLD-Tau (CBD, PSP, and Pick’s) and 4 patients with AD were obtained from the UCD Alzheimer’s Disease Center (Supplemental Table 1).

### Human iMGL.

Human iPSCs were routinely maintained in Hung-Chih Kuo’s laboratory at the Institute of Cellular and Organismic Biology, Academia Sinica, Taiwan, as previously described (61). The sex and APOE genotype of human iPSCs are listed in Supplemental Table 2. Microglial differentiation was conducted according to an established protocol elsewhere with slight modification (20) as detailed in Supplemental Methods.

### pTau preparation.

Recombinant pTau (1N4R) was routinely prepared as previously described except that the size exclusion FPLC chromatography step was omitted (21). Briefly, pTau and tau were expressed using the constructs pMK1013-GSK-tau(1N4R) and pMK1013-tau(1N4R), respectively. The constructs were transformed into the *E*. *coli* BL21 codon plus strain and cultured overnight. Cells were cultured to an OD_600_ between 0.3 and 0.5 before the addition of IPTG. After 2 hours, cells were pelleted and resuspended in 10 mL of cold purification buffer (20 mM Tris-HCL pH 5.8, 100 mM NaCl, 1 mM PMSF, and 0.2 mM orthovanadate). Subsequently, 1 mg/mL of lysozyme was added and the mixture was incubated at 30°C for 30 minutes. The mixture was then sonicated (Branson Digital Sonifier 450; 30% amplitude; Pulse-ON time 5 seconds and Pulse-OFF time 5 seconds for a total of 3 minutes) and centrifuged at 17,000*g* for 40 minutes at 4°C. The supernatant was boiled and cooled down on ice for 30 minutes, respectively. The mixture was then subjected to centrifugation at 7,000*g* for 50 minutes at 4°C. The supernatant was transferred to a new tube and added with 0.5 mM DTT and 1 mM EDTA. For digestion, TEV protease was added (1 OD280 TEV: 100 OD280 sample) and incubated at 4°C overnight. The mixture was centrifuged at 7,000*g* for 30 minutes at 4°C, and the resulting supernatant was transferred to a new tube. Gel filtration was performed using a spin column (Amicon Centrifugal Filter Unit, Ultra-15, 10 kD) at 5,000*g* for 20 minutes at 4°C to reduce the volume to 1–2 mL. A total of 4 mL of gel filtration buffer (20 mM Tris-HCL pH 7.4, 100 mM NaCl) was added to the spin column, and the buffer exchange step was repeated twice. Finally, the supernatant was transferred to a new tube and aliquoted for storage at –80°C. Size of recombinant proteins were analyzed with SDS-PAGE and TOOL Start Blue Staining Reagent according to the manufacturer’s protocol (TTD-BS1L, TOOLS). Endotoxin in the recombinant proteins were detected by using an endotoxin ELISA kit (abx514093, Abbexa) and Pro-Q Emerald 300 LPS gel stain kit (P20495, Thermo Fisher Scientific). All recombinant proteins had negligible endotoxin levels below detection ranges (0.015—1.0 EU/mL) at the working concentration (50 nM). All batches of pTau were produced at Michigan State University in the laboratory of Min-Hao Kuo and were validated to meet quality control standards. Specifically, the purified pTau was shown to form thioflavin S-reactive amyloid under inducer-free conditions and caused at least 50% cell death in SH-SY5Y cells within 24 hours at a concentration of 1 μM. Cell death was quantified by propidium iodide and fluorescein diacetate differential staining.

### EV isolation and preparation.

The conditioned medium (15 mL) harvested from iMGL was centrifuged at 2,000*g* for 30 minutes to exclude cell debris. For concentration, the supernatants were transferred into Amicon Ultra15 Centrifugal Filter Units (3K) devices and centrifuged at 4,000*g* for 45–55 minutes. The concentrated culture media (1 mL) were transferred into a sterile 1.5 mL microcentrifuge tube, and 0.5 volumes of Total Exosome Isolation reagent (Invitrogen) were added. The concentrated culture media and the reagent were mixed by pipetting to achieve a homogenous solution, followed by incubation at 4°C overnight. After incubation, the samples were centrifuged at 10,000*g* for 1 hour at 4°C. Supernatants were carefully removed without disturbing the EV pellets at the bottom of the tubes. EV pellets were either resuspended in PBS for NTA and cell treatments or in RIPA lysis buffer for immunoblotting.

To compare the levels of Gal3 in EV and non-EV secreted forms, EVs were prepared following a protocol for EV isolation detailed elsewhere (62), with the addition of a concentration step using Amicon Ultra15 Centrifugal Filter Units (3K) devices as described above. EVs were pelleted from 150 μL of concentrated medium, and the supernatant was carefully collected. The pelleted EVs were then resuspended in 150 μL of RIPA lysis buffer. We compared Gal3 levels by using equal 50-μL volumes of both the supernatant and EV lysates for Western blot analysis. The Proteinase K protection assay was conducted as detailed elsewhere (29). EVs were treated with 1 mg/mL proteinase K for 30 minutes at 37°C. A control aliquot, without proteinase K, was incubated under the same condition.

### Treatment of microglia and neurons.

iMGL were pretreated with 10 μM TD139 (B2266-5, BioVision) or 0.1% DMSO as a vehicle control for 24 hours before treatment with pTau (50 nM) or fibrillar Aβ (1 μM) for 6 hours, followed by the collection of the iMCM and cell samples for RNA preparation.

SY5Y-tau cells were treated with iMCM for 24 hours before the assay. For neutralizing antibody treatments, 3 μg/mL B2C10 (an anti-Gal3 neutralizing antibody that binds Gal3 at the first 18 amino acids of N-terminus (14, 63), 556904, BD Pharmingen) was coincubated with iMCM and SY5Y-tau cells for 24 hours. SY5Y-tau cells were treated with recombinant Gal3 (1 μM) or EVs (10 μg/mL) for 24 hours before assays. Recombinant Gal3 was conjugated with Atto-565 fluorescence dye (^Atto565^rGal3) by using a Lightning-Link Rapid Atto 565 Conjugate system (351-0030, Innova BioSciences) and used to treat SY5Y-tau cells for 4 hours to evaluate the ability of Gal3 to enter cells.

### RNA extraction, cDNA synthesis, and quantitative PCR.

Total RNA was extracted from hippocampal tissues and iMGL by using the GENEzol TriRNA Pure Kit (Geneaid Biotech) and subsequently reverse transcribed into cDNA by using Superscript III (Invitrogen) according to the manufacturer’s protocol. RT–qPCR was performed by using SYBR Green PCR Master Mix (Life Technologies) with amplification on a LightCycler 480 Real-Time PCR System (Roche Life Science) or ABI QuantStudio 5 Real-Time PCR System (Applied Biosystems). Relative expression of genes of interest were determined by using the ΔΔCT method. For normalization, *Gapdh* and *18S* served as the housekeeping genes for mice and humans, respectively. The expression levels of genes of interest were normalized to those of the respective reference gene, and relative expression levels were calculated. Primers used for the quantification of genes of interest are listed in Supplemental Table 8.

### scRNA-Seq of microglia.

Adult microglial isolation was performed according to the protocol detailed elsewhere (39). For single-cell gene expression library construction, FACS-sorted cells were immediately captured in droplets that were emulsified with gel beads by using the 10× Genomics Chromium Next GEM Single Cell 3’ GEM, Library & Gel Bead Kit v3.2 reagent (10× Genomics) according to the manufacturer’s protocols. These libraries were sequenced using a NovaSeq 6000 (Illumina). The details of the bioinformatics analysis of microglia are provided in the Supplemental Methods.

### Statistics.

Data in histograms and dot plots are expressed as the mean ± SEM, while violin plots show the medians with the 25th and 75th percentiles. Statistical comparisons were performed with GraphPad Prism 9 software using the 2-tailed unpaired Student’s *t* test (for 2 comparison groups with normal distribution criteria) or 1- or 2-way ANOVA with Tukey’s multiple comparison test (for groups across variables, comparing all groups to each other). For all tests, a *P* value < 0.05 was considered significant. All experiments were conducted in at least triplicate unless otherwise indicated. No statistical methods were applied to predetermine the sample size.

### Study approval.

Human brain samples were obtained from the UCD Alzheimer’s Disease Center. All animal studies were conducted following protocols approved by the Institutional Animal Care and Utilization Committee (IACUC, Academia Sinica, Taiwan).

### Data availability.

All data presented in this study are either included in this article and its Supplemental Information or are available upon reasonable request to the corresponding author. Uncropped Western blots can be found in the Supplemental Material. Supplemental data and Supporting Data Values are provided with this paper. RNA data SRA files have been deposited in the NCBI’s Sequence Read Archive (https://www.ncbi.nlm.nih.gov/sra; PRJNA1017614 and PRJNA1018595).

## Author contributions

JJS designed, performed experiments, analyzed data and wrote the manuscript with the assistance of CWL, HLL, and TNAN. HMC bred and maintained animals used in the experiments. YMC, HTL, TMK, and SYC designed, performed, and analyzed scRNA-Seq. FLC and HCK maintained and provided iPSCs used in the experiments. ML, HTH and MHK synthesized and provided pTau used in the study. FTL provided *Lgals3^–/–^* mice, conceptual advice, and materials. DB and LB provided THY-Tau22 mice used in the study. LWJ provided human brain samples. HLC, YCS, and JRH provided recombinant Gal3 proteins in the study. YC designed and supervised the study and edited the manuscript.

## Supplementary Material

Supplemental data

Supplemental table 1

Supplemental table 2

Supplemental table 3

Supplemental table 4

Supplemental table 5

Supplemental table 6

Supplemental table 7

Supplemental table 8

Supporting data values

## Version 1 11/21/2023

In-Press Preview

## Version 2 01/16/2024

Electronic publication
