# Supplementary material for: Galectin-3 aggravates microglial activation and tau transmission in tauopathy
Source: J Clin Invest. 2024 Jan 16;134(2):e165523. doi: 10.1172/JCI165523 (PMC10786694; doi:10.1172/JCI165523)
Supplement: Supplemental data [file jci-134-165523-s077.pdf]

Supplemental Figure 1. Upregulation of Gal3 in patients of tauopathy, related to Figure 1.

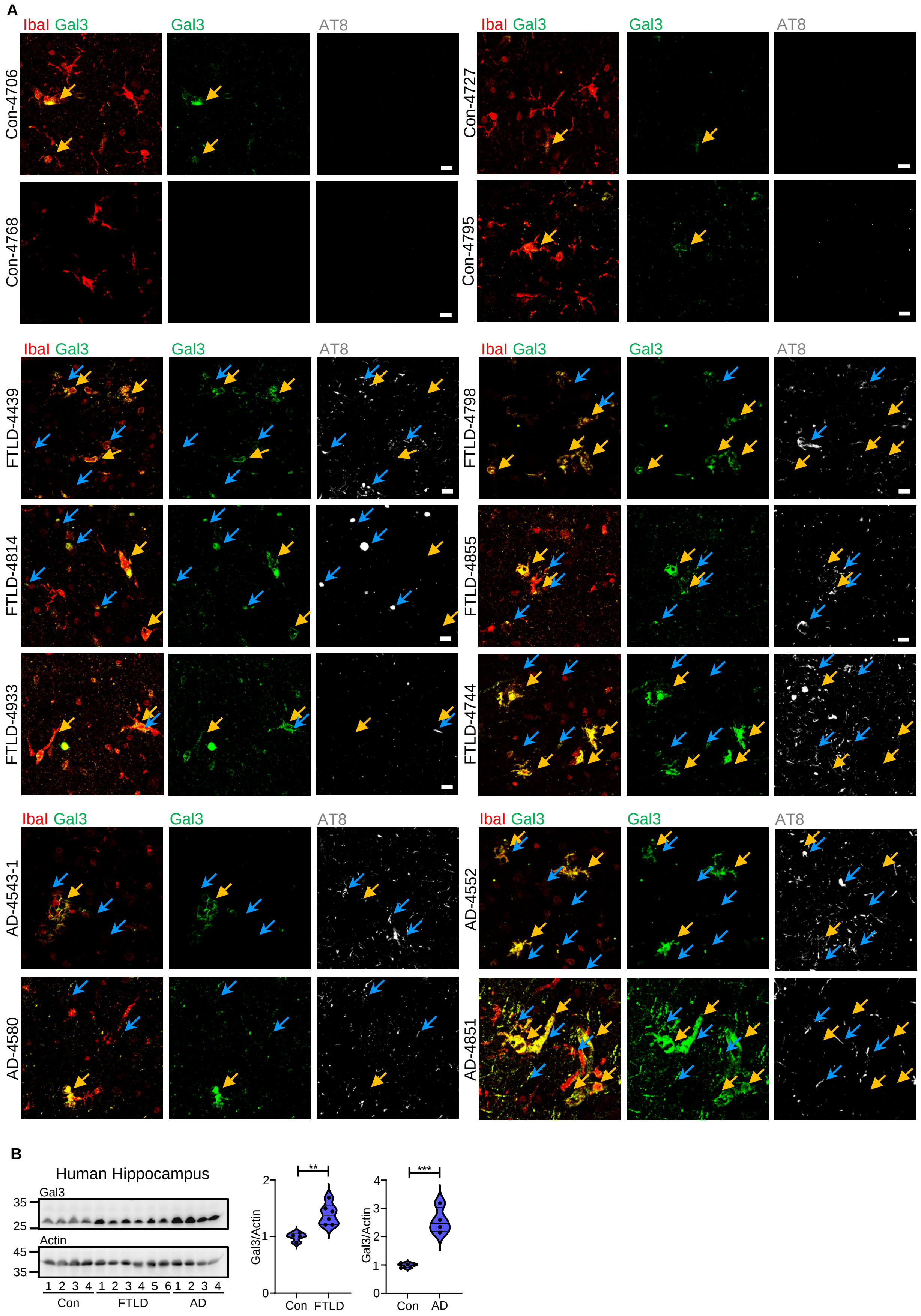

Supplemental Figure 1. Upregulation of Gal3 in patients of tauopathy, related to Figure 1. (A) Immunofluorescence staining of Gal3 (green), AT8 (gray) and microglia (IbaI, red) in the cortexes of frontotemporal lobar dementia (FTLD) patients, Alzheimer's disease (AD) patients and controls (Con), n = 4 for Con and AD, n = 6 for FTLD. For the sake of simplicity, only a portion of microglia with Gal3 expression are marked with orange arrows, and a portion of AT8 signals are marked with blue arrows. **(B)** Immunoblot detection of Gal3 in the hippocampus of FTLD patients, AD patients and controls, n = 4 for Con and AD, n = 6 for FTLD. Data were analyzed with two-tailed unpaired *t* test, and violin plots show medians with 25th and 75th percentiles, $^{**}P < 0.01$, $^{***}P < 0.001$. Scale bar, 10 μm.

Supplemental Figure 2. Expression of microglial markers on human iMGL, related to Figure 1.

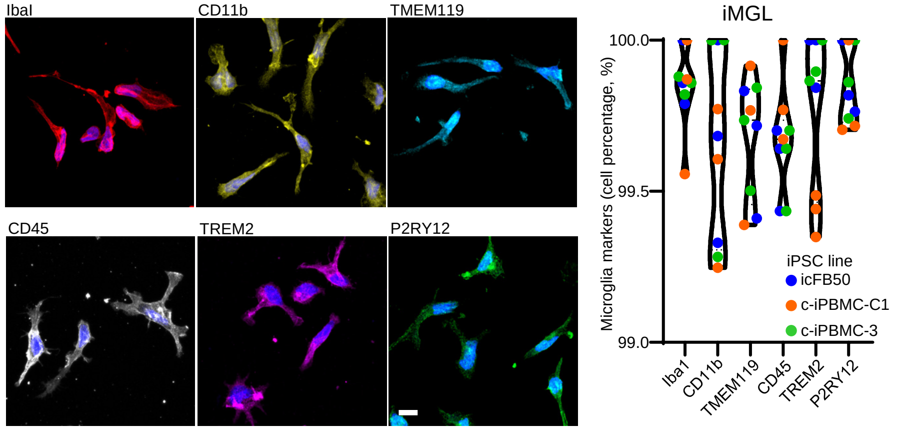

Supplemental Figure 2. Expression of microglial markers on human iMGL, related to Figure 1. Immunofluorescence staining and quantification of 3 independent lines of iMGL (c-iPBMC-3, icFB50 and c-iPBMC-C1) that express microglial markers after 44 days of differentiation (IbaI, CD11b, TMEM119, CD45, TREM2 and P2RY12). Scale bar, 10 µm.

Supplemental Figure 3. Characterization of tau and hyperphosphorylated tau, related to Figure 1.

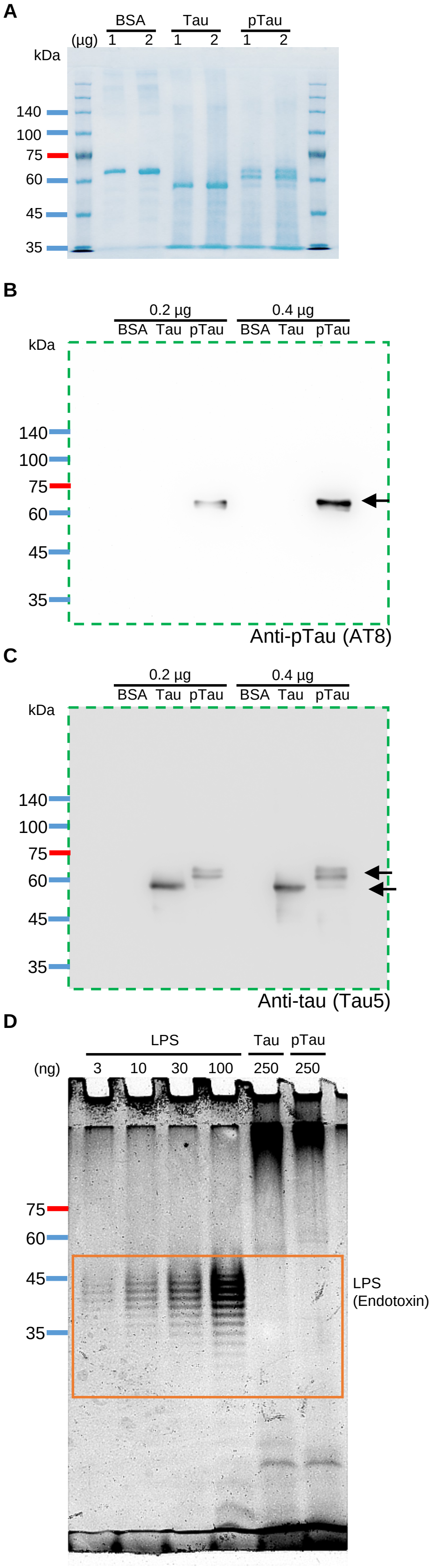

Supplemental Figure 3. Characterization of recombinant tau and recombinant hyperphosphorylated tau produced through expression of tau (pMK1013-tau(1N4R)) and co-expression of tau with GSK3β (pMK1013-GSK-tau(1N4R)) in *Escherichia coli*, related to Figure 1. (A) Detection of the size of recombinant tau and pTau by using TOOL Start Blue Staining Reagent. **(B-C)** Immunoblot detection of recombinant tau (Tau5), which recognizes total tau protein, and of phosphorylated tau using AT8, which recognizes phosphorylated tau (pTau) at Ser202 and Thr205. **(D)** Detection of potential endotoxins that might be present in purified recombinant Tau and pTau by using the Pro-Q ® Emerald 300 Lipopolysaccharide Gel Stain Kit. LPS (3-100 ng) was included as positive control for endotoxin detection. The orange box indicates the area of interest in the gel that contains endotoxin staining. No endotoxins were detected in the recombinant Tau and pTau proteins tested.

Supplemental Figure 4. RNA-seq analysis of iMGL with pTau treatment and Gal3 inhibition, related to Figure 2.

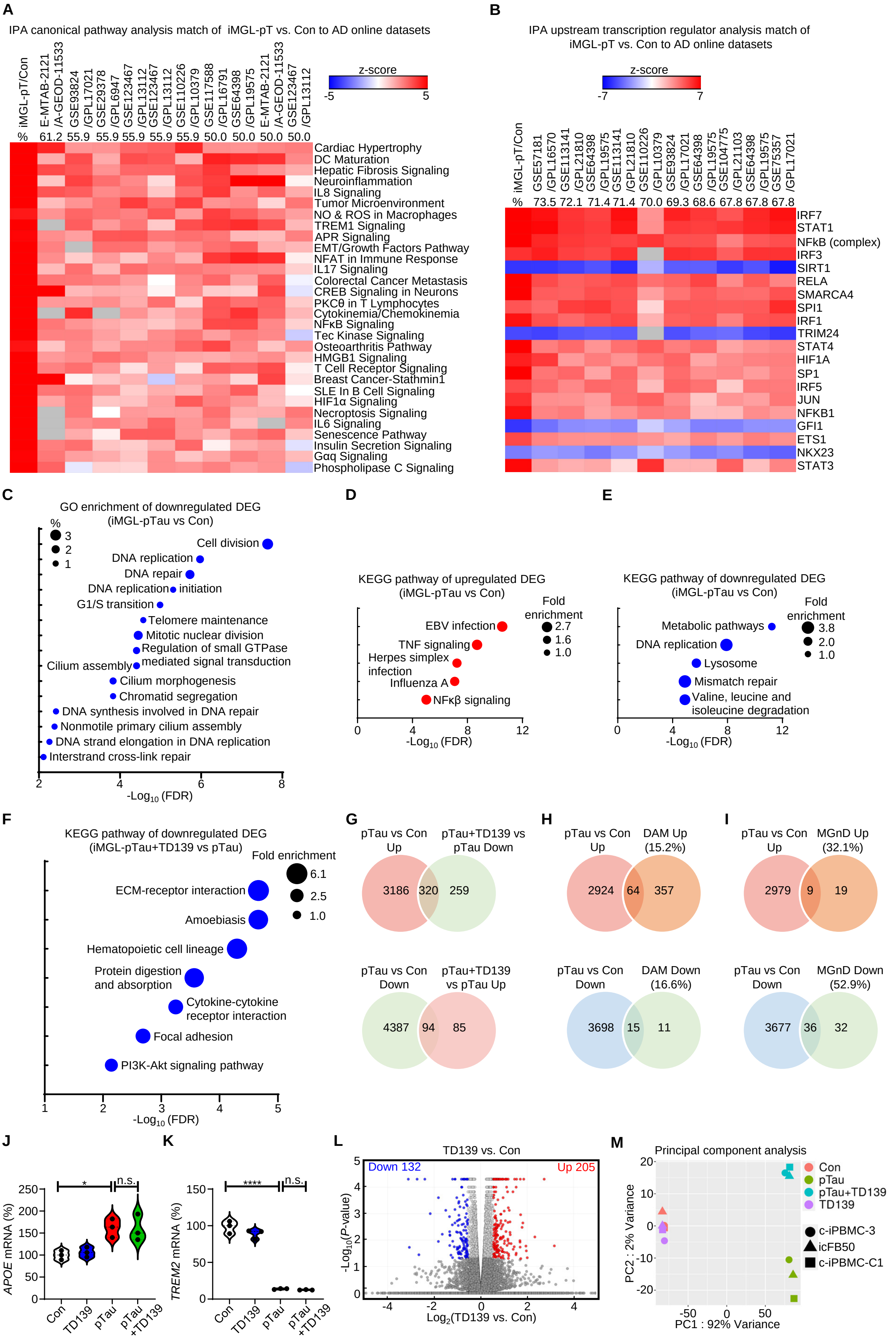

Supplemental Figure 4. RNA-seq analysis of iMGL with pTau treatment and Gal3 inhibition, related to Figure 2. (A) Comparison of the canonical pathways of upregulated DEGs in iMGL treated with pTau for 6 hours that matched online AD datasets. **(B)** IPA prediction of upstream transcriptional regulators of upregulated DEGs in iMGL treated with pTau that matched online AD datasets. **(C)** GO analysis of downregulated DEGs in iMGL cells treated with pTau. **(D-E)** Kyoto Encyclopedia of Genes and Genomes (KEGG) analysis of the pathway enrichment of upregulated and downregulated DEGs in pTau vs. Con iMGL. **(F)** KEGG analysis of the pathway enrichment of downregulated DEGs in pTau plus TD139 vs. pTau iMGL. **(G)** Venn diagrams show the numbers of normalized genes in each group. **(H)** Venn diagrams show the comparison of DEGs in pTau vs Con and in DAM genes. **(I)** Venn diagrams show the comparison of DEGs in pTau vs Con and in MGnD genes. **(J-K)** qPCR analysis of *APOE* and *TREM2* in each iMGL group, n = 3 iMGL lines. Data were analyzed with two-way ANOVA, and violin plots show medians with 25th and 75th percentiles, $*P < 0.05$, $****P < 0.0001$. **(L)** Volcano plots show the DEGs identified in the TD139 vs. Con. Red and blue dots indicate upregulated and downregulated DEGs, respectively. Cutoff of significance, $|\text{Log2 FC}| > 0.55$ and $P < 0.05$. **(M)** Principal component analysis of iMGL RNA-seq.

Supplemental Figure 5. Gal3-associated microglial-early responsive (Gal3-ER) genes, related to Figure 2.

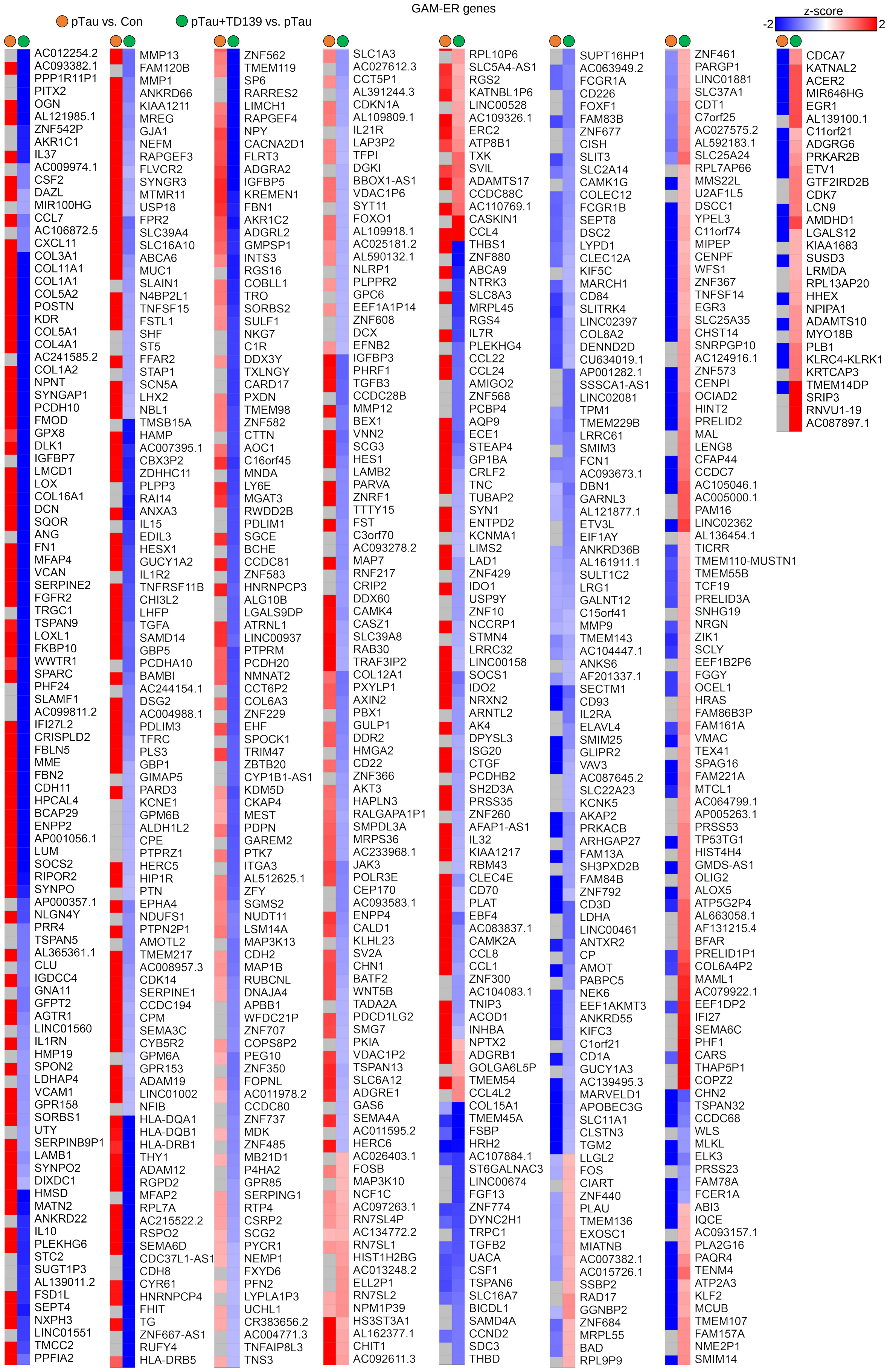

Supplemental Figure 5. Gal3-associated microglial-early responsive (Gal3-ER) genes, related to Figure 2. Heatmap shows the comparison of 758 Gal3-ER genes (i.e., DEGs identified between pTau+TD139 vs. pTau iMGL) to those identified between pTau vs. Con iMGL.

Supplemental Figure 6. Effects of Gal3 inhibition by TD139 at different timepoints, related to Figure 2.

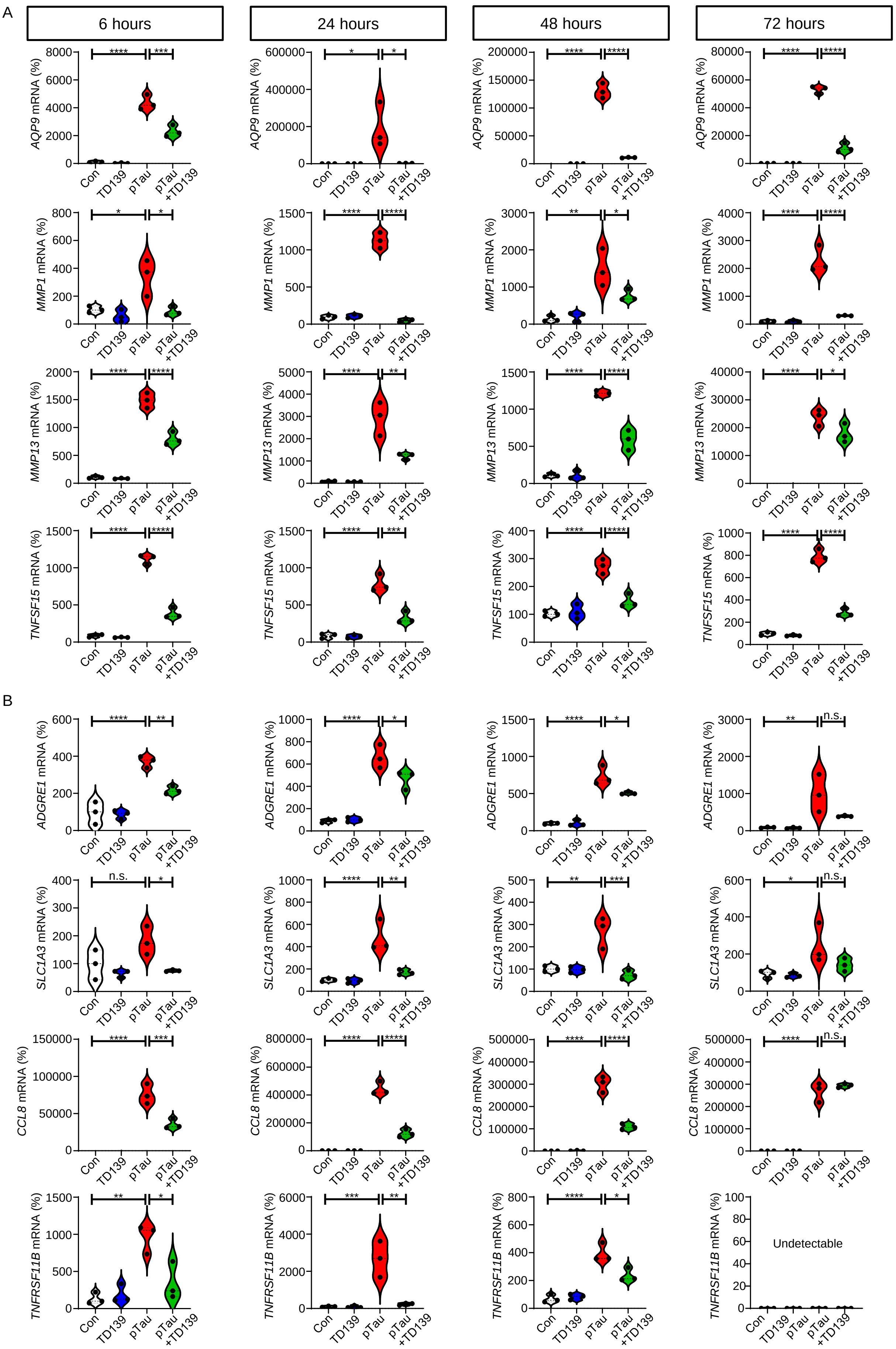

Supplemental Figure 6. Effects of Gal3 inhibition by TD139 at different timepoints, related to Figure 2. (A-B) qPCR analysis of *AQP9*, *MMP1*, *MMP13*, *TNFSF15*, *ADGRE1*, *SLC1A3*, *CCL8* and *TNFSF11B* in the iMGL from the indicated treatment groups at 6, 24, 48 and 72 hours. The expression level of *TNFSF11B* is below detection sensitivity at 72 hours. Data were analyzed with two-way ANOVA, and violin plots show medians with 25th and 75th percentiles, $*P < 0.05$, $**P < 0.01$, $***P < 0.001$ $****P < 0.0001$.

Supplemental Figure 7. Microglia and Gal3 increase the amount misfolded tau in SY5Y-tau cells, related to Figure 3.

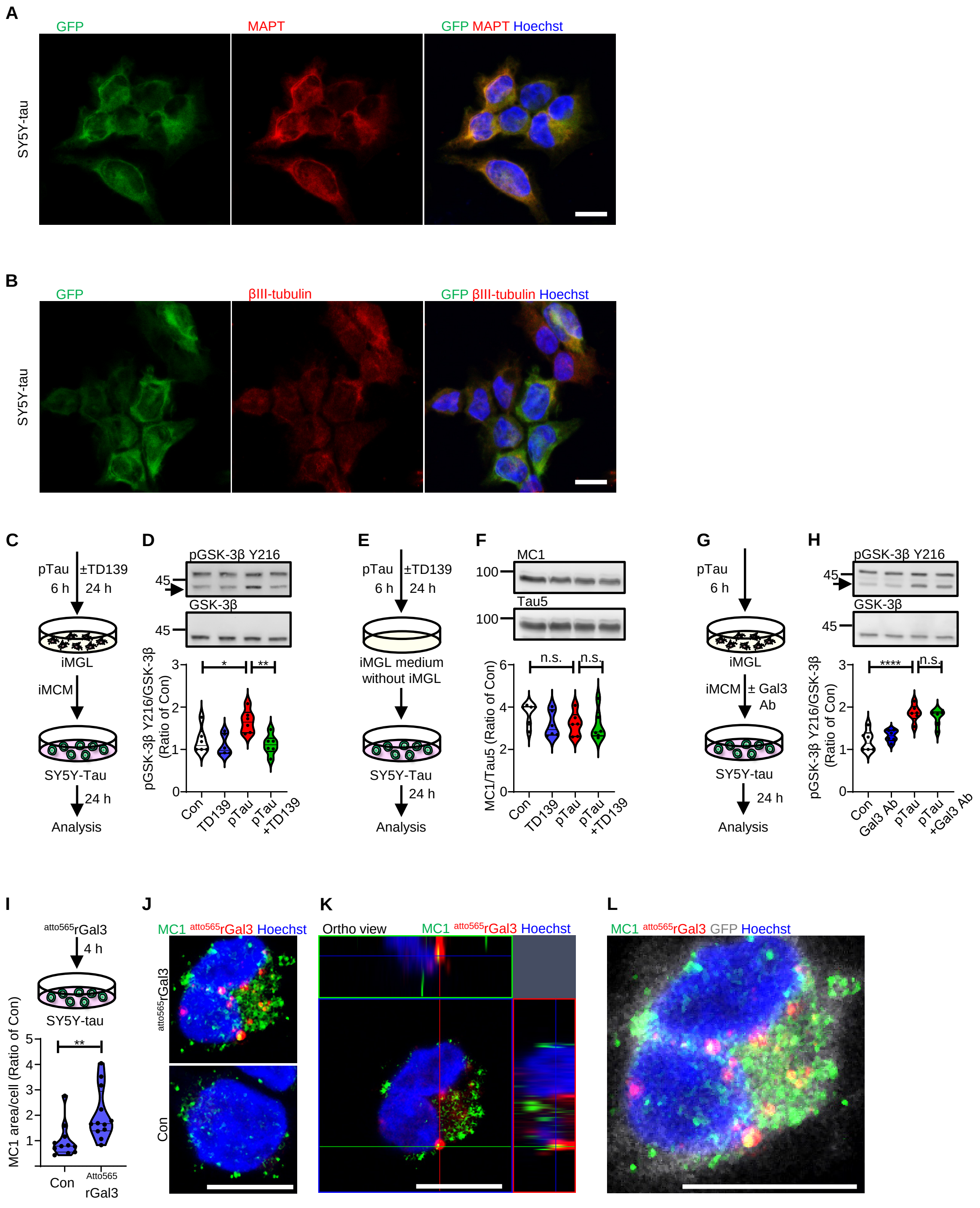

Supplemental Figure 7. Microglia and Gal3 increase the amount misfolded tau in SY5Y-tau cells, related to Figure 2. Immunofluorescence staining of MAPT **(A)** and βIII-tubulin **(B)** in SY5Y-tau cells, GFP representing the endogenous signal without staining. **(C-D)**, Immunoblot analysis and quantification of pGSK-3β-Y216 in SY5Y-tau cells treated with iMCM collected from each group, n = 6. **(E-F)** Immunoblot analysis and quantification of MC1 in SY5Y-tau cells treated with fresh iMGL medium with or without pTau and TD139 but without iMGL, n = 6. **(G-H)** Immunoblot analysis and quantification of pGSK-3β-Y216 following coincubation with pTau iMCM and anti-Gal3 neutralizing antibody, n = 6. **(I-J)** Immunofluorescence staining of MC1 (gray, recolored to green) and labeling of ^{Atto565}rGal3 (red) in SY5Y-tau cells, n = 3, 3-4 fields per coverslip. **(K)** Orthogonal view of the data in **J** to illustrate the colocalization of MC1 with ^{Atto565}rGal3. **(L)** Inclusion of GFP signals (green, recolor to gray) to highlight the cells. Scale bar, 10 μm. Data in **D**, **F**, **H** were analyzed by two-way ANOVA with Tukey's test, **I** was analyzed with the two-tailed unpaired *t* test and violin plots show medians with 25th and 75th percentiles, $^{*}P < 0.05$, $^{**}P < 0.01$, $^{****}P < 0.0001$.

Supplemental Figure 8. Analysis of EVs produced by microglia in the presence of pTau and Gal3 inhibition, related to Figure 3.

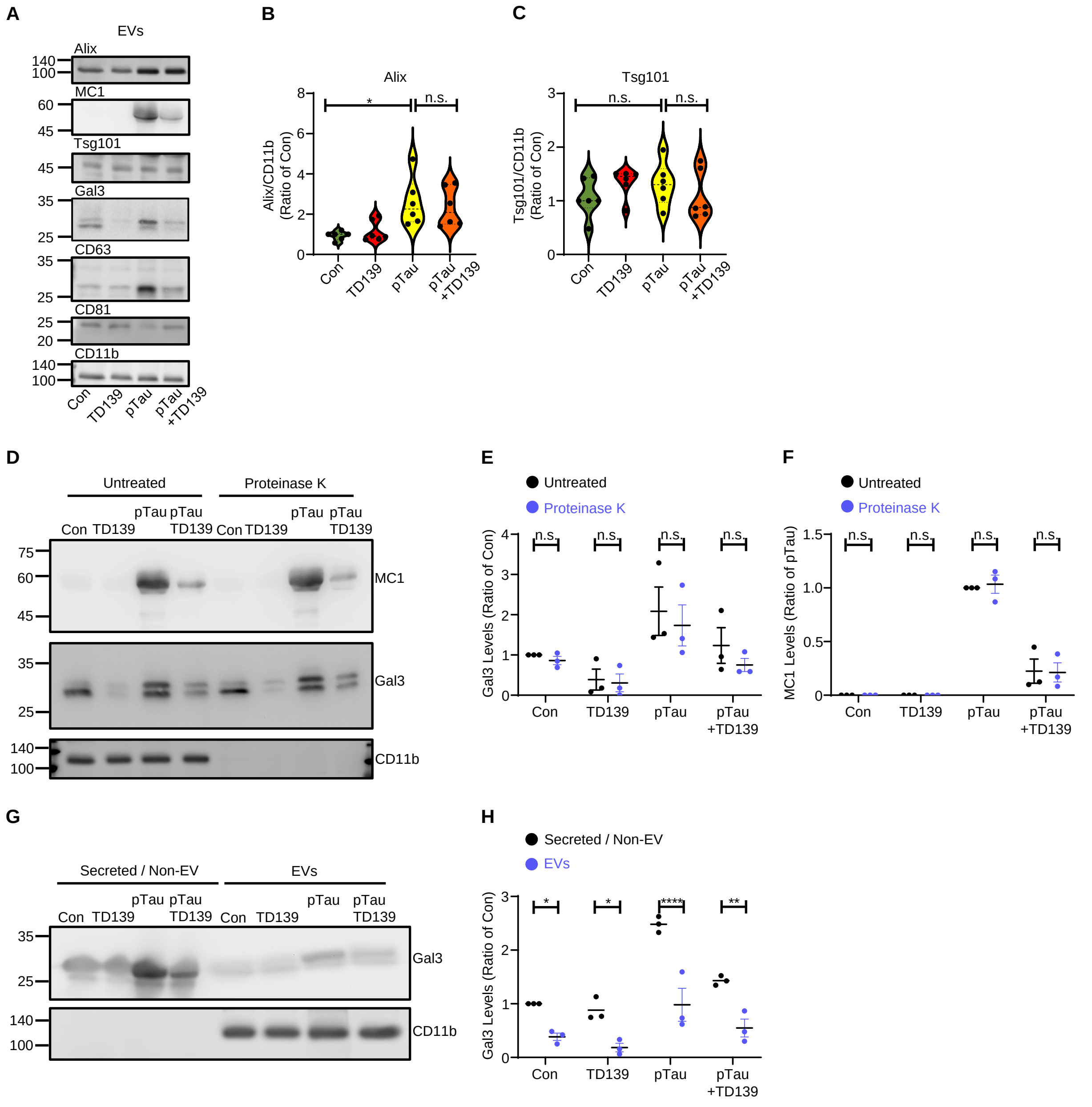

Supplemental Figure 8. Analysis of EVs produced by microglia in the presence of pTau and Gal3 inhibition, related to Figure 3. (A-C) Immunoblot analysis and quantification of Alix and Tsg101 in EVs, n = 6, 3 iMGL lines from 2 independent differentiations. **(D-F)** Immunoblot analysis and quantification of MC1 and Gal3 in EVs with or without Proteinase K treatments, n = 3 iMGL lines. **(G-H)** Immunoblot analysis and quantification of the levels of Gal3 in the secreted/Non-EV form and in the EV form, n= 3 iMGL lines. Data in **B-F** were analyzed by two-way ANOVA with Tukey's test, **H** were analyzed by two-way ANOVA with Newman-Keuls's test, and violin plots show medians with 25th and 75th percentiles, and dot plots show mean with standard error of mean (SEM), *$P < 0.05$, **$P < 0.01$, ****$P < 0.0001$.

Supplemental Figure 9. Aggregation assays of pTau with ThS in the presence of various compounds, related to Figure 3.

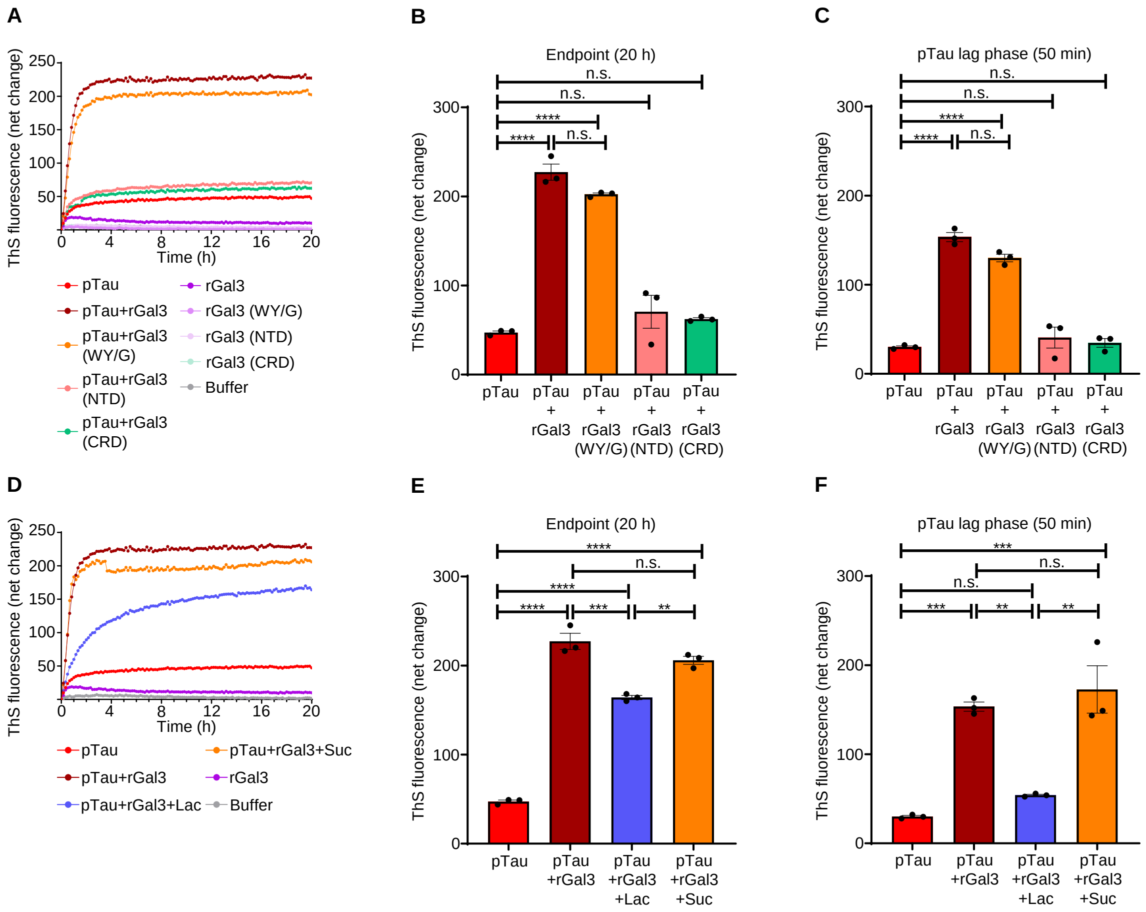

Supplemental Figure 9. Aggregation assays of pTau with ThS in the presence of various compounds, related to Figure 3. (A) pTau aggregation assay conducted with different forms of Gal3, including the C-terminus (CRD), N-terminus (NTD) and a mutant form with the replacement of aromatic residues (WY/G), from tryptophans and tyrosines to glycines, resulting in the loss of LLPS activity. Quantification of **A** in **(B)** endpoint and **(C)** lag phase of pTau. **(D-F)** pTau aggregation assay conducted using lactose (Lac) that binds to Gal3, and sucrose (Suc) as a non-binding control. Quantification of **D** in **(E)** endpoint and **(F)** lag phase of pTau. Data were analyzed by one-way ANOVA with Tukey's test and presented as mean with SEM, $^{**}P < 0.01$, $^{***}P < 0.001$, $^{****}P < 0.0001$.

Supplemental Figure 10. Detection of endogenous Gal3 in the microglia near to AT100-positive neurons and labeling of rGal3 in the hippocampus of Tau22 mice and, related to Figure 4.

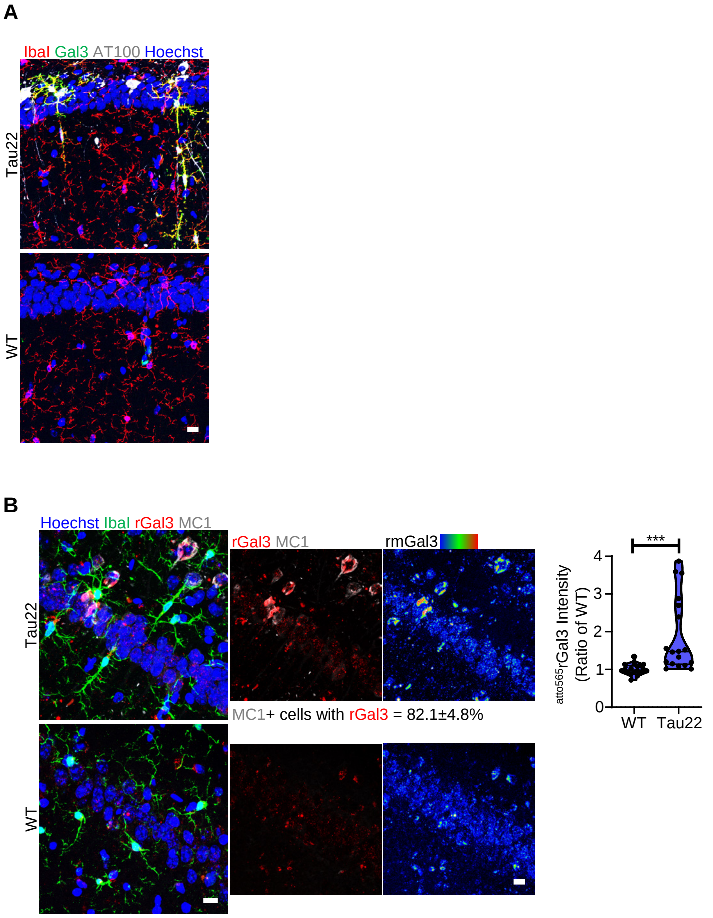

Supplemental Figure 10. Detection of endogenous Gal3 in the microglia near to AT100-positive neurons and labeling of rGal3 in the hippocampus of Tau22 mice, related to Figure 4. (A) Immunohistochemical staining of microglia and Gal3 close to the CA1 region with aggregated tau (AT100) in Tau22 mice and the corresponding brain region in WT mice. **(B)** Immunohistochemical staining of microglia and MC1 and labeling of rGal3 in Tau22 and WT mice, n = 6 mice, 3 fields per animal. Scale bar, 10 µm. Data were analyzed with the two-tailed unpaired *t* test. Violin plots show medians with 25th and 75th percentiles, $***P < 0.001$.

Supplemental Figure 11. Quality control metrics of scRNA-seq data, cell proportion and pseudotime of microglia, related to Figure 4.

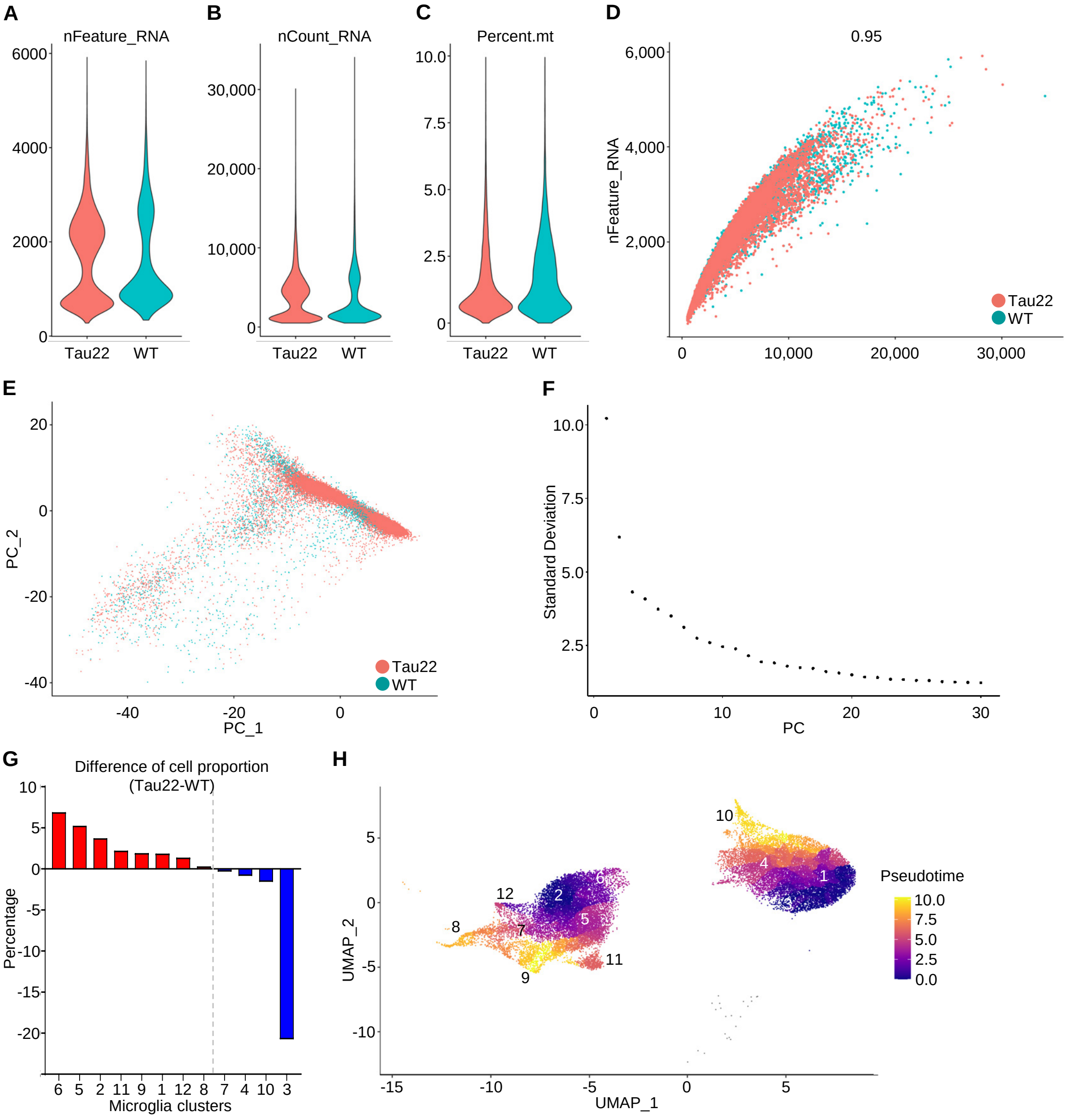

Supplemental Figure 11. Quality control metrics of scRNA-seq data, cell proportions and pseudotime of microglia, related to Figure 4. (A) Violin plots show the UMI counts for Tau22 and WT samples. **(B)** Violin plots show the total number of genes for each sample. **(C)** Violin plots show the percentage of mitochondrial genes per cell for each sample. **(D)** Scatter plot showing the correlation between the total number of genes and the total number of UMI counts, with a Pearson correlation of 0.95. **(E)** Scatterplot showing principal component analysis (PCA) PC1 and PC2. **(F)** Elbow plot showing the standard deviation of the first 30 principal components obtained from PCA. Principle components 1 to 30 were used as the input for subsequent UMAP analysis. **(G)** Percentage of microglial cell proportion difference in Tau22 and WT mice. **(H)** Pseudotime trajectory analysis of microglia.

Supplemental Figure 12. Heatmap shows marker genes identified from each microglial cluster, related to Figure 4.

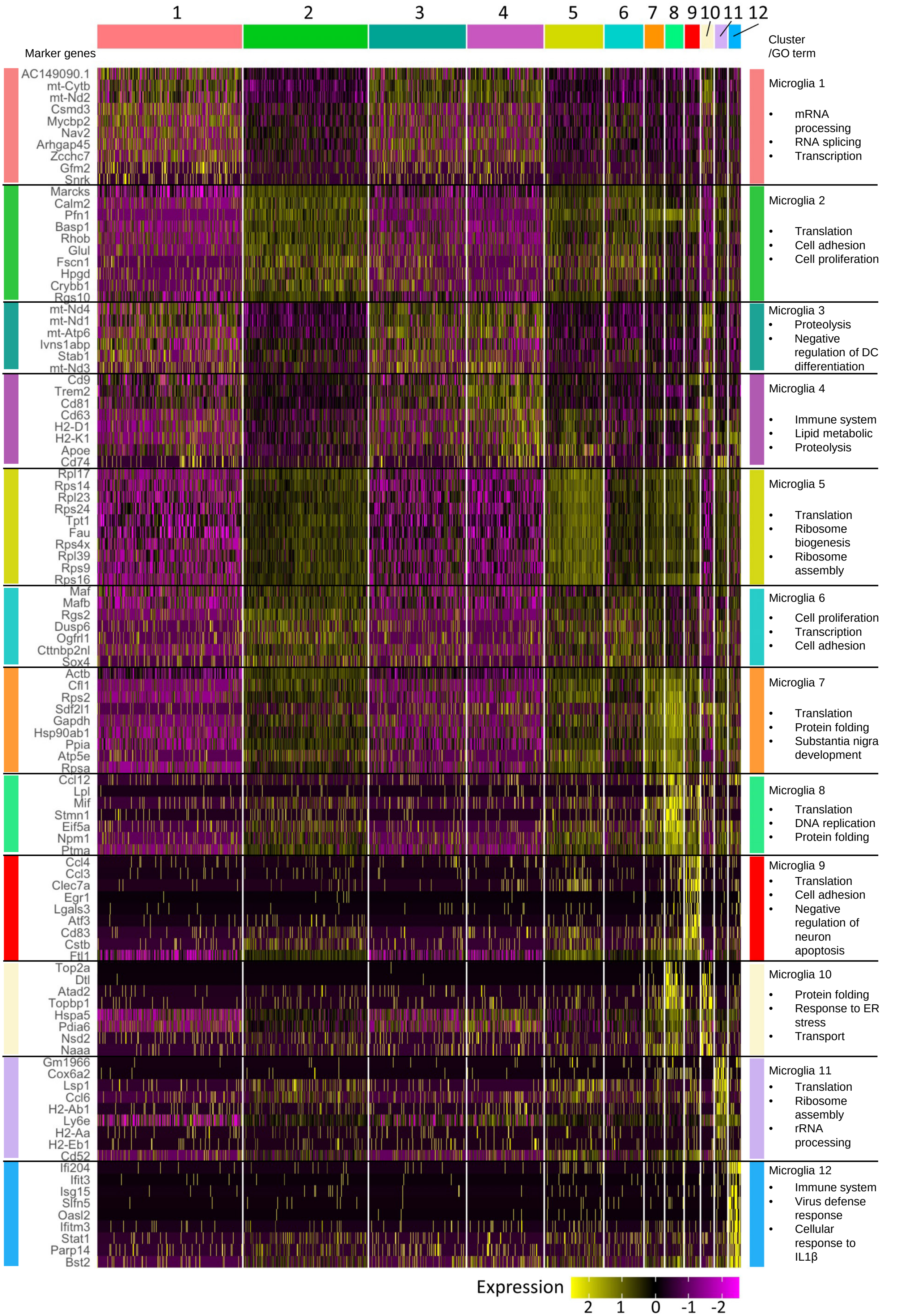

Supplemental Figure 12. Heatmap shows marker genes identified from each microglial cluster, related to Figure 4. Top 10 marker genes identified by Seurat from each microglial cluster. The property of the cluster was determined by the top biological processes identified by DAVID.

Supplemental Figure 13. Expression levels of microglial marker genes from each cluster, related to Figure 4.

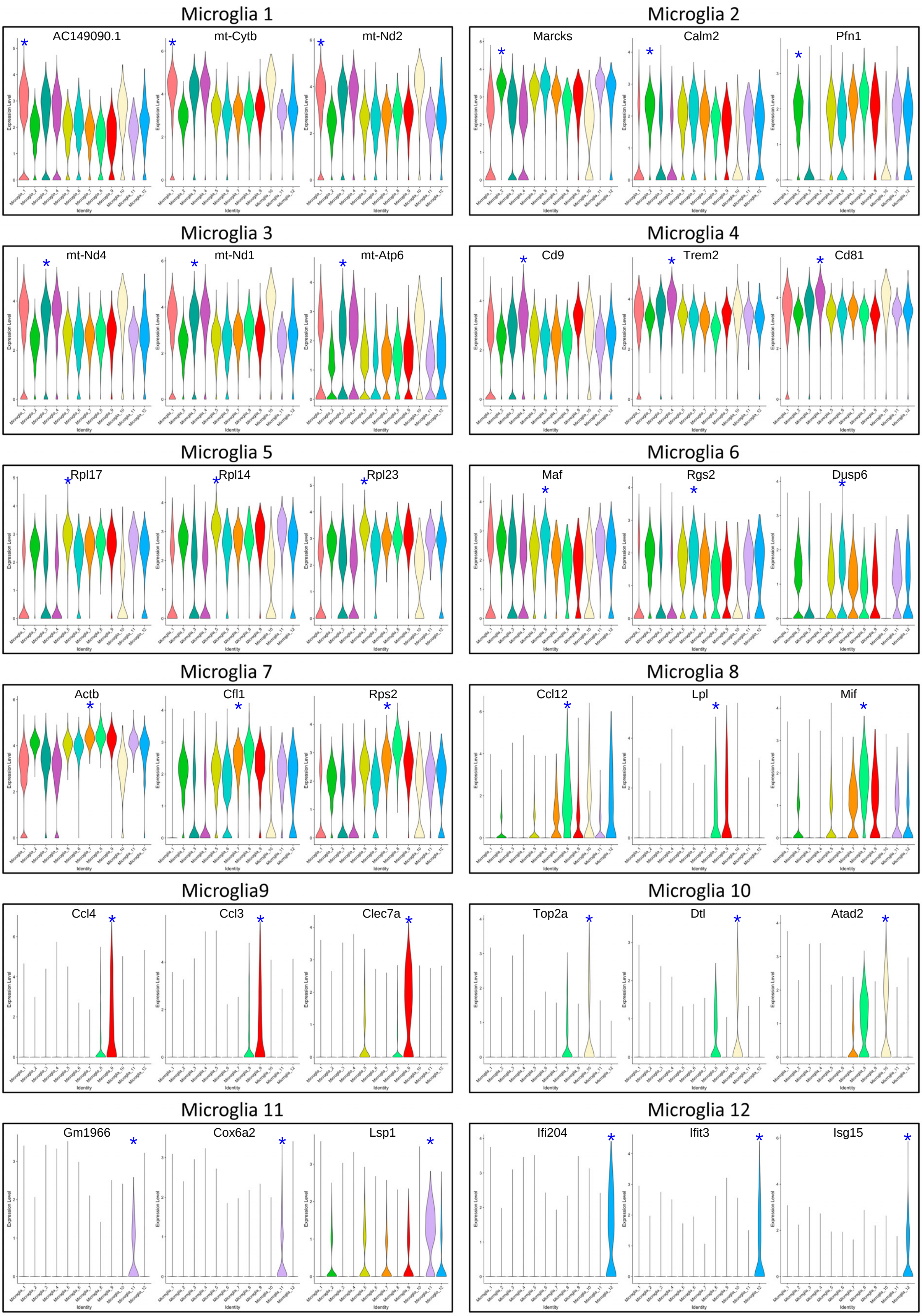

Supplemental Figure 13. Expression levels of microglial marker genes from each cluster, related to Figure 4. Violin plots show the top 3 marker genes in each microglial cluster. Blue asterisks (*) indicate the marker genes in respective clusters.

Supplemental Figure 14. Alluvial plot of the top five GO terms per cluster, related to Figure 4.

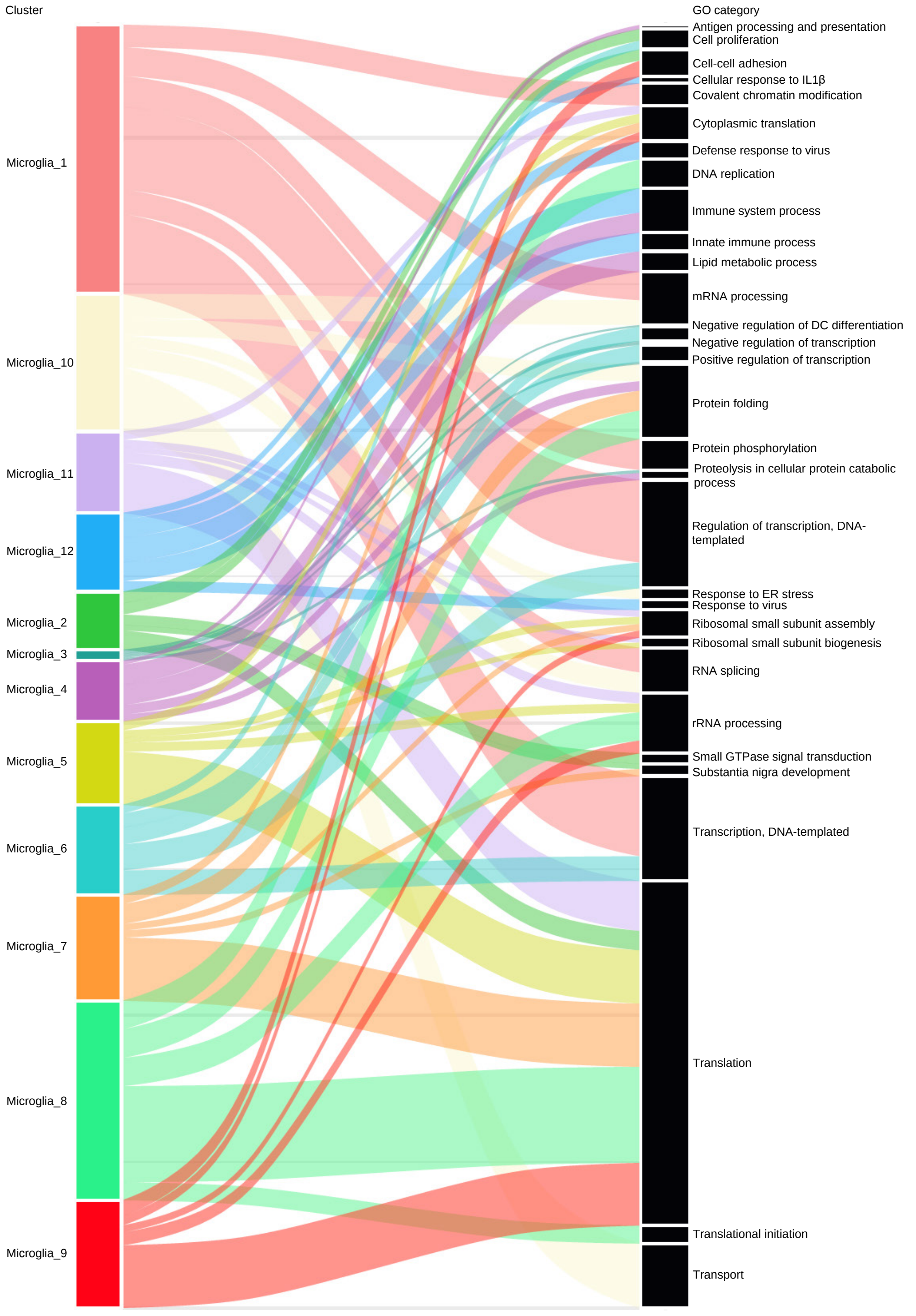

Supplemental Figure 14. Alluvial plot of the top five GO terms per cluster, related to Figure 4. Alluvial plot of twelve microglial clusters depicting the top five GO terms per cluster. The thickness of the ribbon reflects the number of genes associated with each GO term.

Supplemental Figure 15. Enrichments of microglial clusters with the cellular component related to exosomes, multivesicular bodies and endosomes, and the hierarchical clustering of DAM and MGnD genes, related to Figure 4.

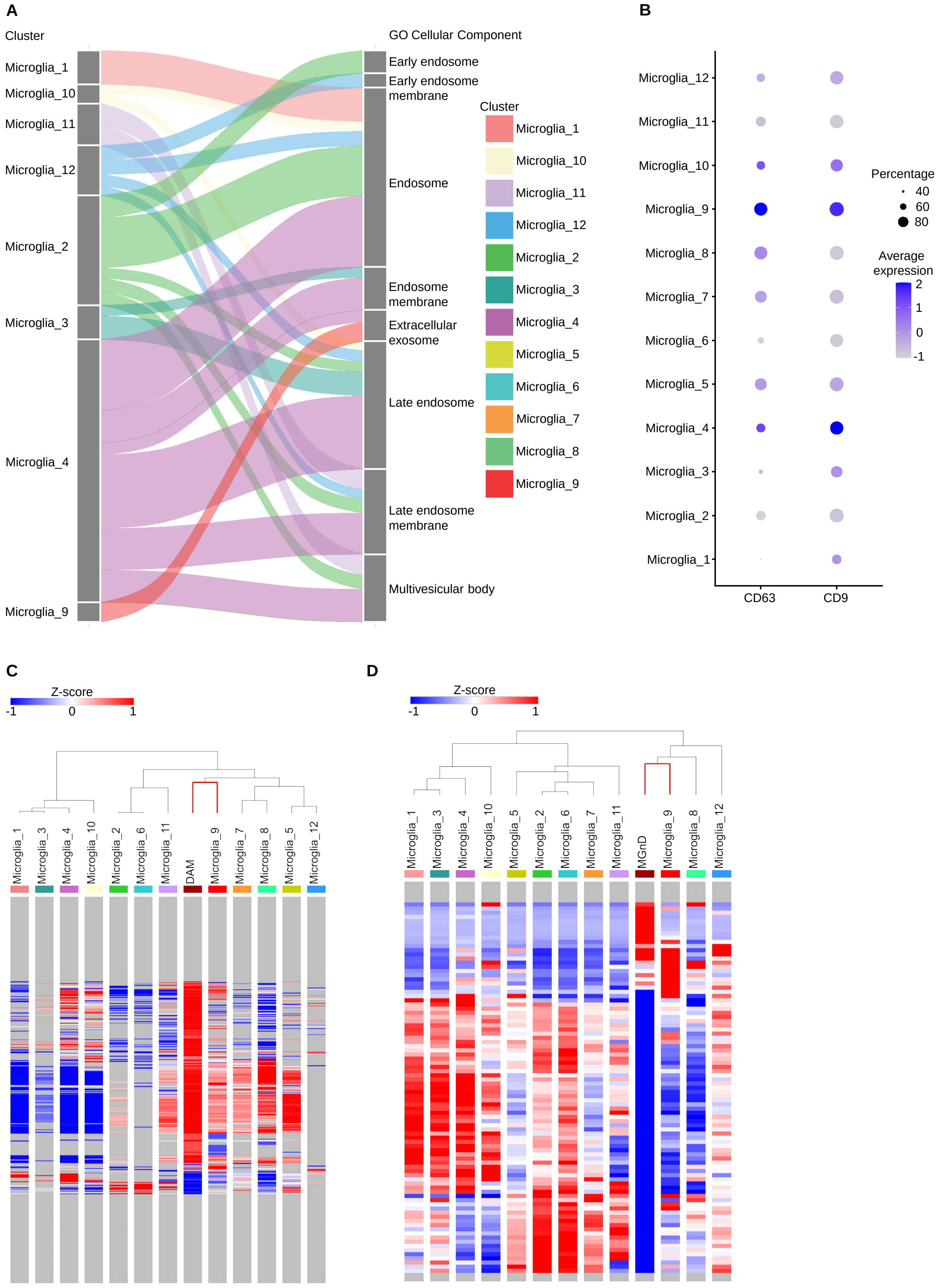

Supplemental Figure 15. Enrichment of microglial clusters with the cellular component related to exosomes, multivesicular bodies and endosomes, and the hierarchical clustering of DAM and MGnD genes, related to Figure 4. (A) Alluvial plot depicting the enrichment of microglial clusters in the cellular components of exosomes, multivesicular bodies and endosomes. **(B)** Dot plot presenting the expression levels of CD63 and CD9 in each microglial cluster. Hierarchical clustering to demonstrate the similarity between microglial cluster and **(C)** DAM genes and **(D)** MGnD genes.

Supplemental Figure 16. GAM vs non-GAM and cellular component related to exosomes, MVBs and endosomes , related to Figure 4.

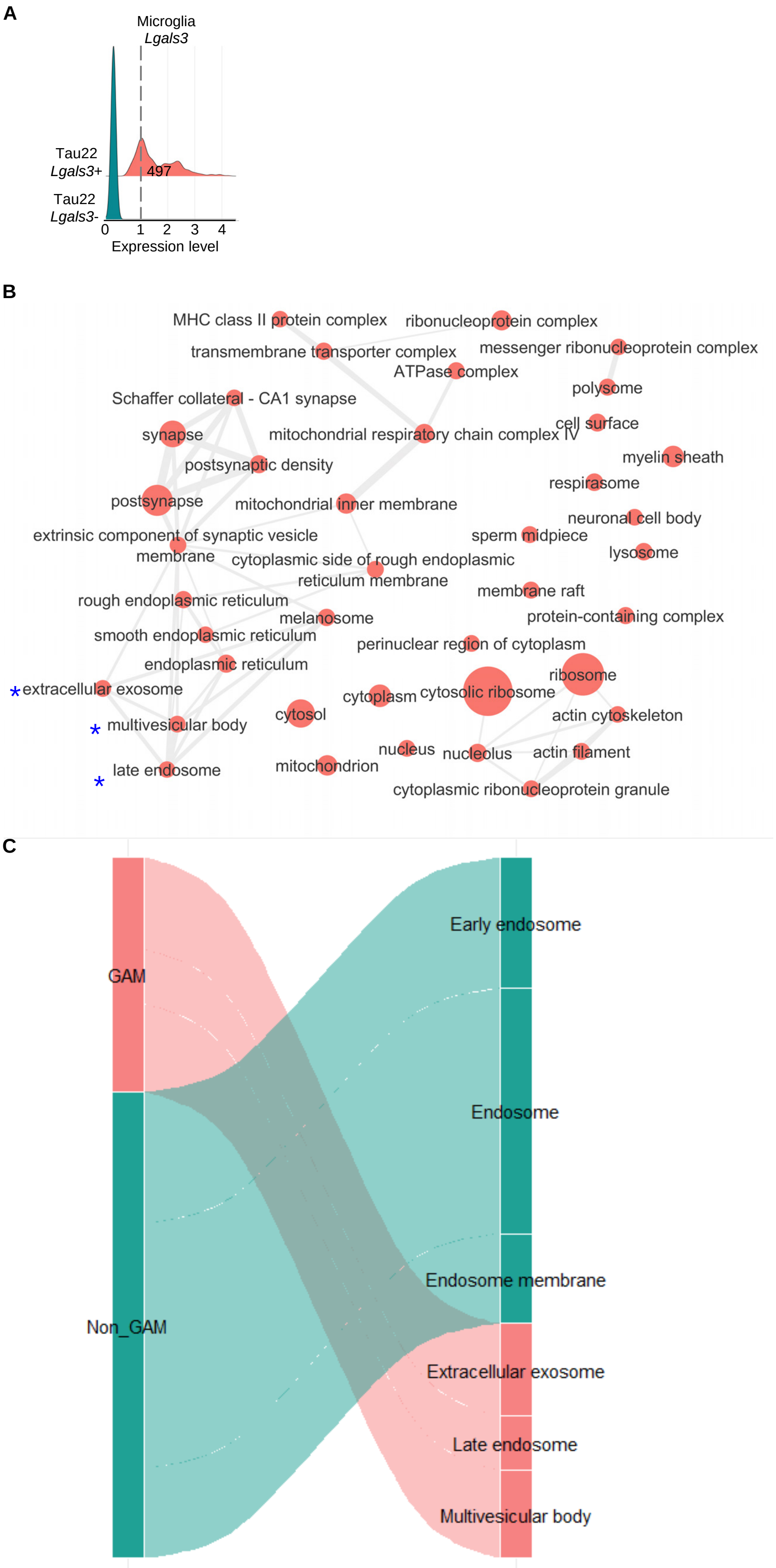

Supplemental Figure 16. GAM vs non-GAM and cellular component related to exosomes, MVBs and endosomes, related to Figure 4. (A) Ridge plot shows the isolation of microglia with average *Lgals3* expression higher than or equal to one from all clusters in Tau22 mice. **(B)** Enrichment map of cellular components by GAM. Blue asterisks (*) indicate the components of exosomes, MVBs and endosomes. **(C)** Alluvial plot depicting the enrichment of GAM and non-GAM in the cellular components of exosomes, multivesicular bodies and endosomes.

Supplemental Figure 17. GAM and DAM genes, related to Figure 4.

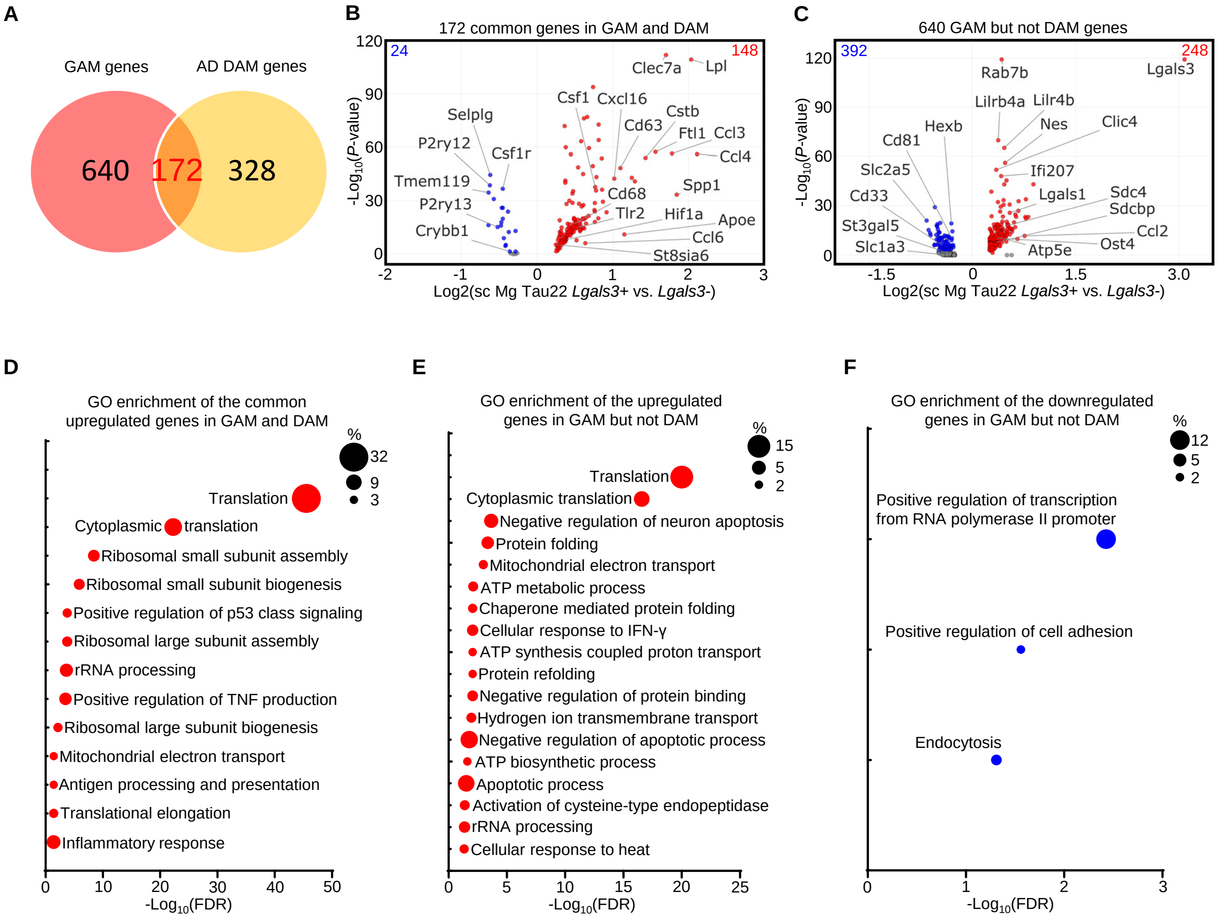

Supplemental Figure 17. GAM and DAM genes, related to Figure 4. (A) Venn diagram shows the comparison of GAM genes and AD DAM genes. Volcano plots showing **(B)** common genes in GAM and DAM and **(C)** genes in GAM but not in DAM. **(D)** GO analysis of common upregulated genes in GAM and DAM. GO analysis of **(E)** upregulated genes and **(F)** downregulated genes in GAM but not in DAM.

Supplemental Figure 18. Comparison of the in vitro pTau induced iMGL response with the microglia in Tau22 mice and Gal3-dependent signatures, related to Figure 2 and 4.

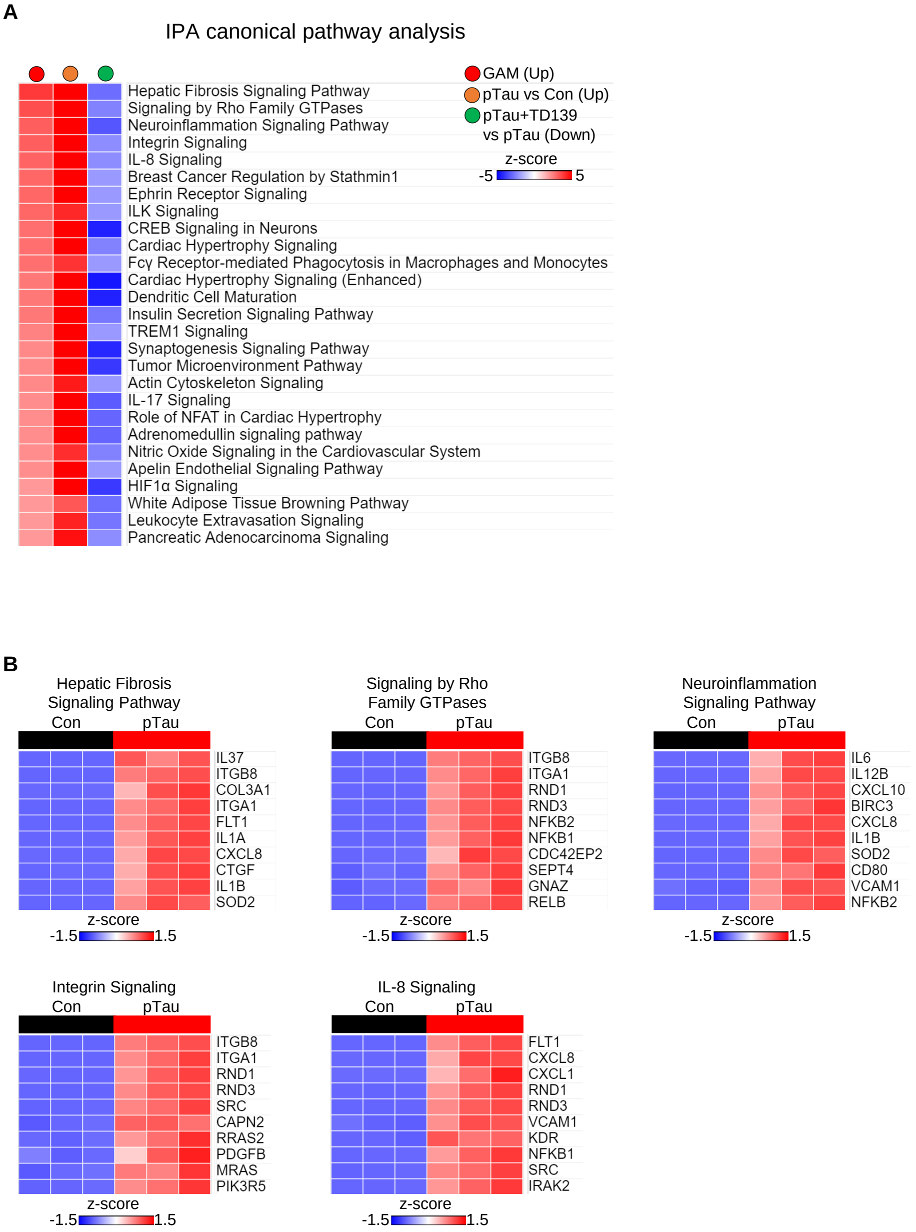

Supplemental Figure 18. Comparison of the in vitro pTau induced iMGL response with the microglia in Tau22 mice and Gal3-dependent signatures, related to Figure 2 and 4. (A) IPA analysis revealing the conservation of canonical pathways among GAM, pTau-activated pathways, and those suppressed by Gal3 inhibition (TD139) in iMGL. **(B)** Top 5 conserved canonical pathways along with their corresponding gene expression levels.

Supplemental Figure 19. Staining of AT8 in Tau22 and Tau22/*Lgals3*$^{-/-}$ mice, related to Figure 5.

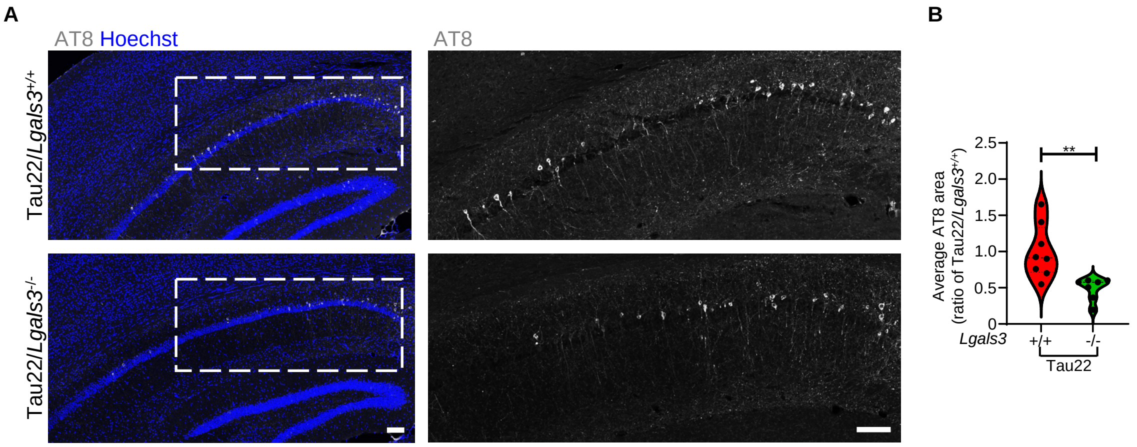

Supplemental Figure 19. Staining of AT8 in Tau22/*Lgals3*$^{-/-}$ and Tau22/*Lgals3*$^{+/+}$ mice, related to Figure 5. (A-B) Immunohistochemical staining and quantification of AT8 in the CA1 region of Tau22/*Lgals3*$^{-/-}$ and Tau22/*Lgals3*$^{+/+}$ mice, n=8 mice. Scale bar, 100 µm. Data were analyzed with the two-tailed unpaired *t* test and violin plots show medians with 25th and 75th percentiles, $^{**}P < 0.01$.

Supplemental Figure 20. Synaptic puncta analysis in the hippocampus, related to Figure 5.

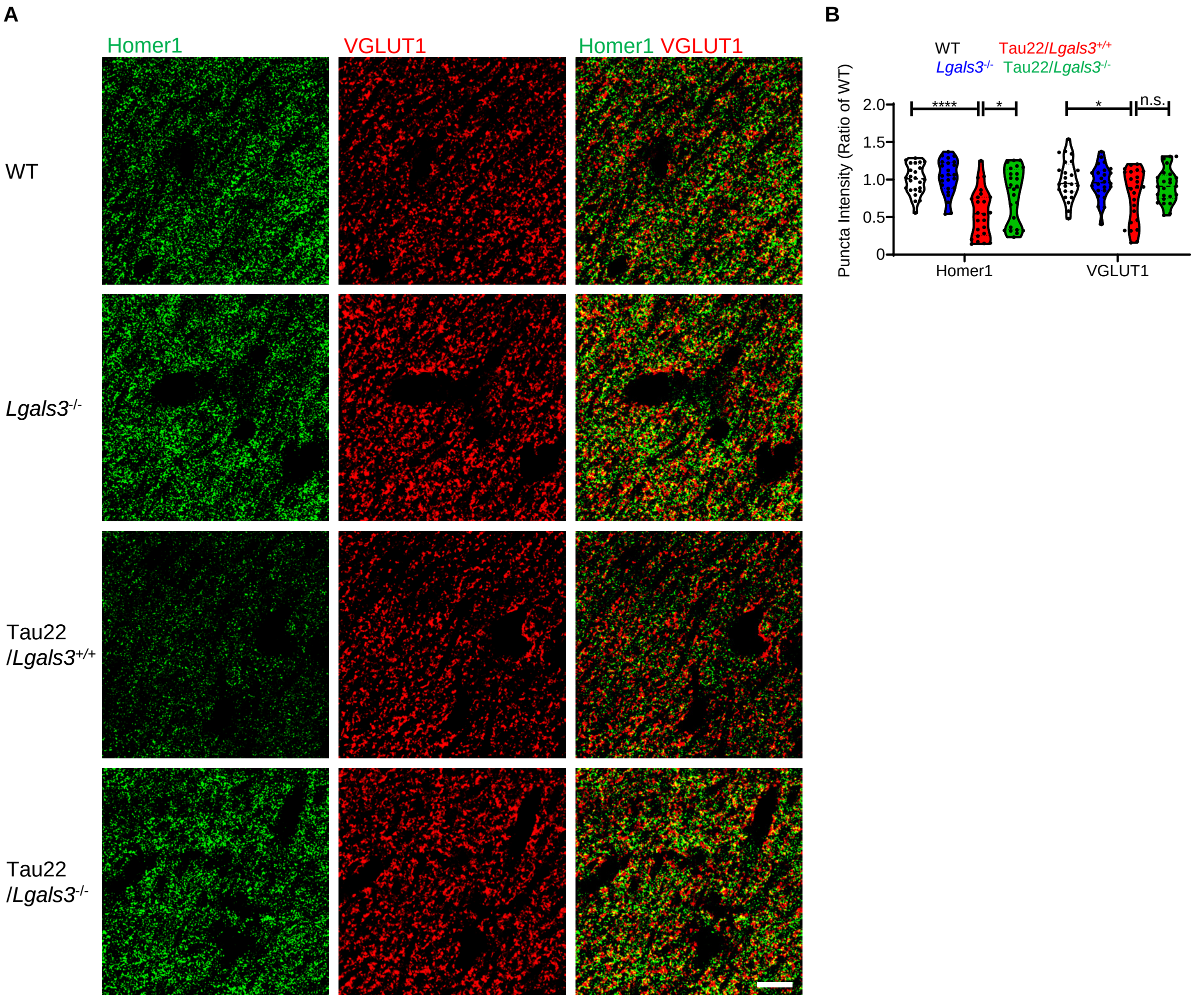

Supplemental Figure 20. Synaptic puncta analysis in Tau22/*Lgals3*$^{+/+}$ and Tau22/*Lgals3*$^{-/-}$ mice and control mice, related to Figure 5. (A) Immunohistochemical staining of the synapse assembly (i.e., colocalization of Homer 1 and VGLUT1 puncta) in the CA1 region of Tau22/*Lgals3*$^{-/-}$ and control mice. **(B)**, Quantification of the staining in **A**, n = 8 mice, 3 fields per animal. Scale bars, 2 µm. Data were analyzed by two-way ANOVA with Tukey's test and all violin plots show the median with the 25th and 75th percentiles. **P* < 0.05, *****P* < 0.0001.

Supplemental Figure 21. RNA-seq analysis of Tau22/Lgals3-/- and control mice, related to Figure 6.

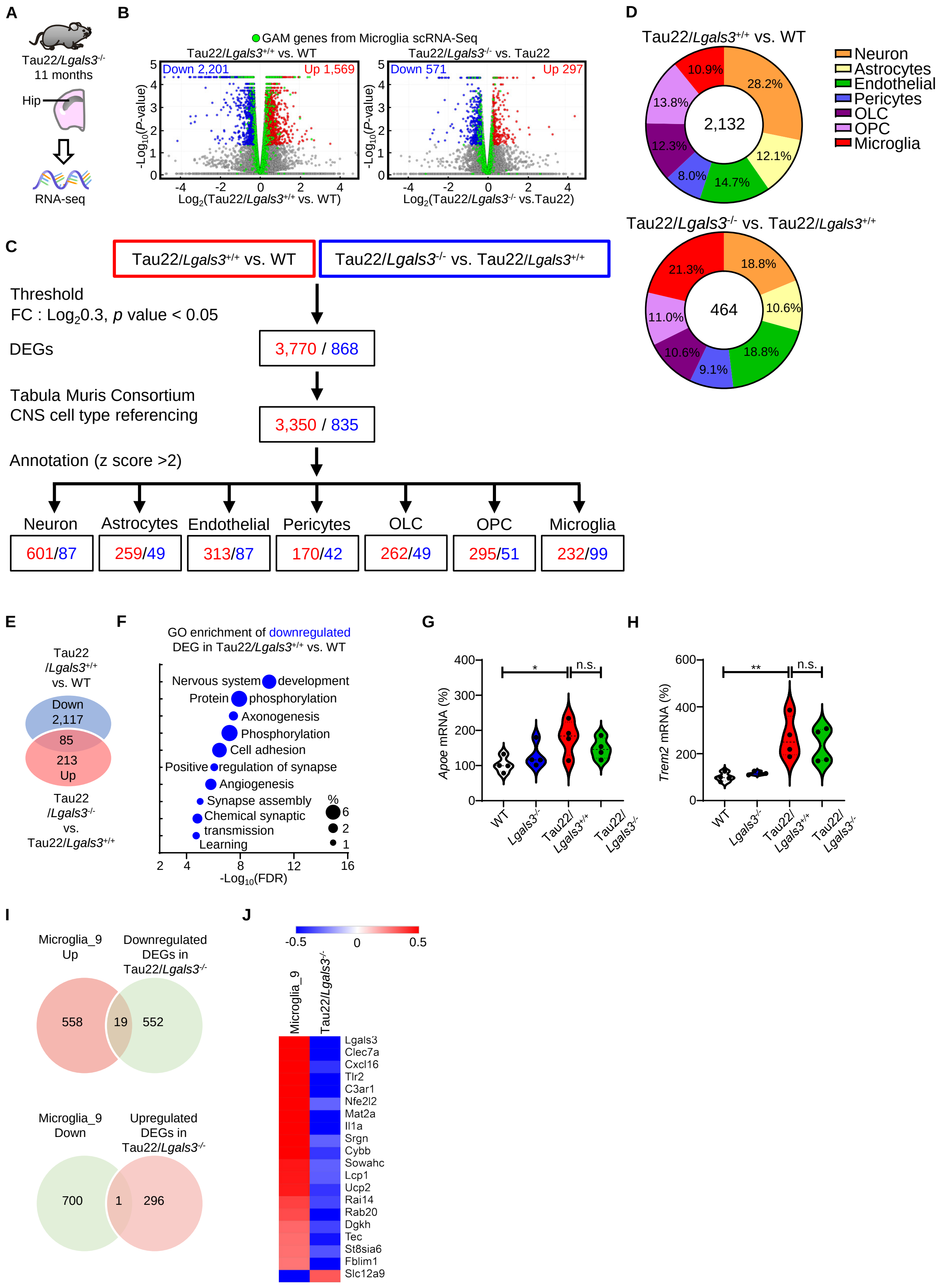

Supplemental Figure 21. RNA-seq analysis of Tau22/*Lgals3*$^{-/-}$ and control mice, related to Figure 6. (A-B) Volcano plots show DEGs identified in Tau22/*Lgals3*$^{+/+}$ vs. WT and Tau22/*Lgals3*$^{-/-}$ vs. Tau22/*Lgals3*$^{+/+}$ mice. Red and blue dots indicate upregulated and downregulated DEGs, respectively, and green dots indicate the overlay of GAM genes identified from scRNA-seq. **(C)** Workflow for the RNA-seq analysis of bulk tissue samples with central nervous system cell type-specific annotation. **(D)** Percentage of DEGs in each brain cell type in Tau22/*Lgals3*$^{+/+}$ vs. WT and Tau22/*Lgals3*$^{-/-}$ vs. Tau22/*Lgals3*$^{+/+}$ mice. **(E)** Venn diagram shows the overlapping of DEGs in the indicated group. **(F)** GO enrichment analysis of downregulated DEGs in Tau22/*Lgals3*$^{+/+}$ vs. WT mice. qPCR analysis of the levels of **(G)** *Apoe* and **(H)** *Trem2* in the indicated genotypes. **(I)** Venn diagrams show the comparison of DEGs in Microglia_9 and DEGs Tau22/*Lgals3*$^{-/-}$ mice. **(J)** Heatmap shows the levels of microglial specific DEGs in Tau22/*Lgals3*$^{-/-}$ mice and in Microglia_9. Data in **G** and **H** were analyzed by two-way ANOVA with Tukey's test and all violin plots show the median with the 25th and 75th percentiles. *$P < 0.05$, **$P < 0.01$.

Supplemental Figure 22. Knockout of Gal3 reduces astrogliosis in Tau22 mice, related to Figure 5/6.

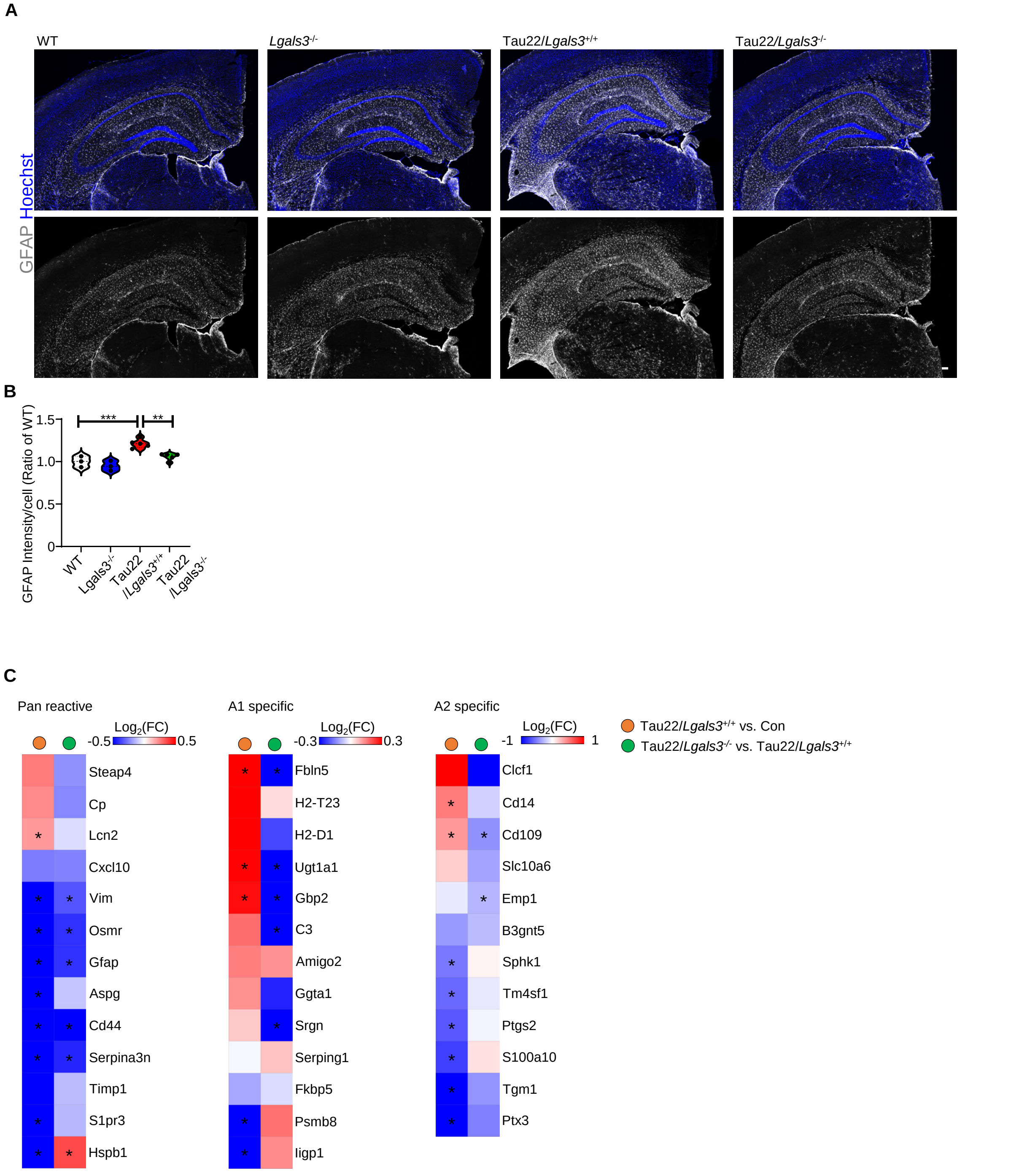

Supplemental Figure 22. Knockout of Gal3 reduces astrogliosis in Tau22 mice, related to Figure 5/6. (A-B) Immunohistochemical staining and quantification of astrocytes (GFAP, gray), n = 3 for WT and *Lgals3*$^{-/-}$ mice, n = 5 for Tau22/*Lgals3*$^{+/+}$ and Tau22/*Lgals3*$^{-/-}$ mice. Scale bar, 100 µm. Data were analyzed by two-way ANOVA with Tukey's test. Violin plots show medians with 25th and 75th percentiles, $^{**}P < 0.01$, $^{***}P < 0.001$. **(C)** Heatmap shows pan-reactive, A1-specific and A2-specific genes in Tau22/*Lgals3*$^{+/+}$ vs. WT and Tau22/*Lgals3*$^{-/-}$ vs. Tau22/*Lgals3*$^{+/+}$ mice, asterisk (*) marks significantly altered DEGs.

Supplemental Figure 23. Morphometric analysis of iMGL treated with amyloid-beta, related to Figure 7.

A

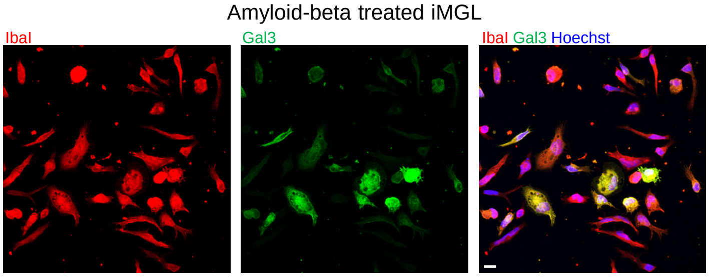

B

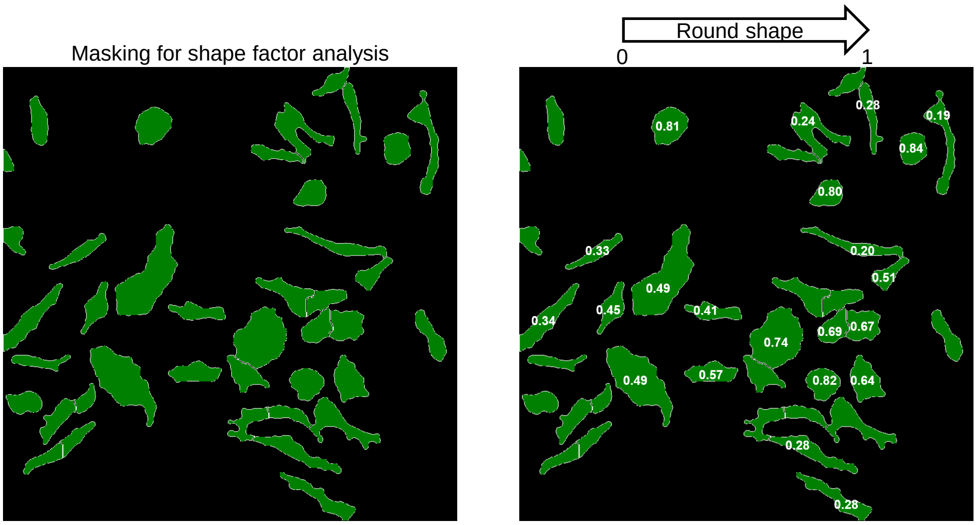

C

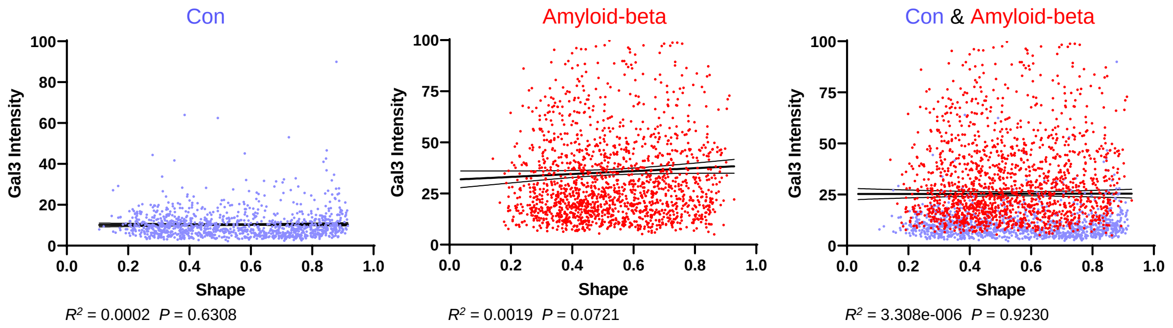

D

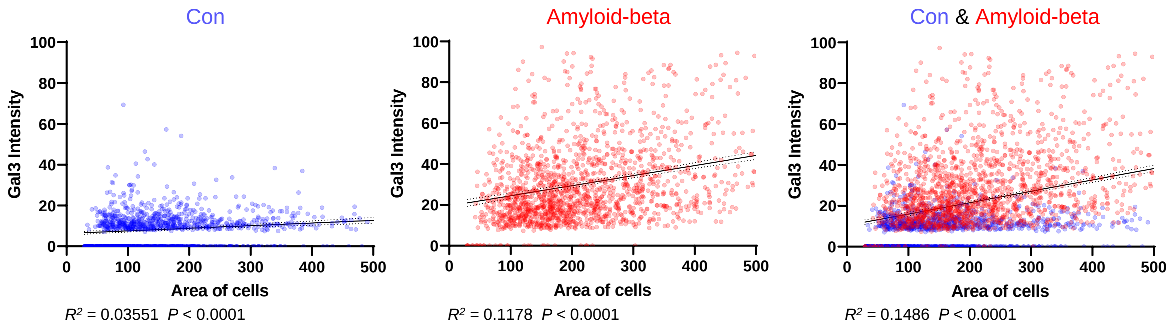

Supplemental Figure 23. Morphometric analysis of iMGL treated with amyloid-beta, related to Figure 7. (A-B) Example demonstrates the masking of iMGL for morphometric analysis. Values ranging from 0 to 1 indicate cells with morphology transitioning from non-rounded to rounded shapes. For the sake of simplicity, only a portion of cells is labeled with the values of round shape scores. Correlation analysis between the **(C)** shape and **(D)** size of iMGL treated with amyloid-beta and the expression levels of Gal3. The scores were evaluated using Pearson correlation coefficients.

Supplemental Figure 24. Staining of amyloid-beta plaques in APP/PS1/*Lgals3*$^{-/-}$ and APP/PS1/*Lgals3*$^{+/+}$ mice, related to Figure 7.

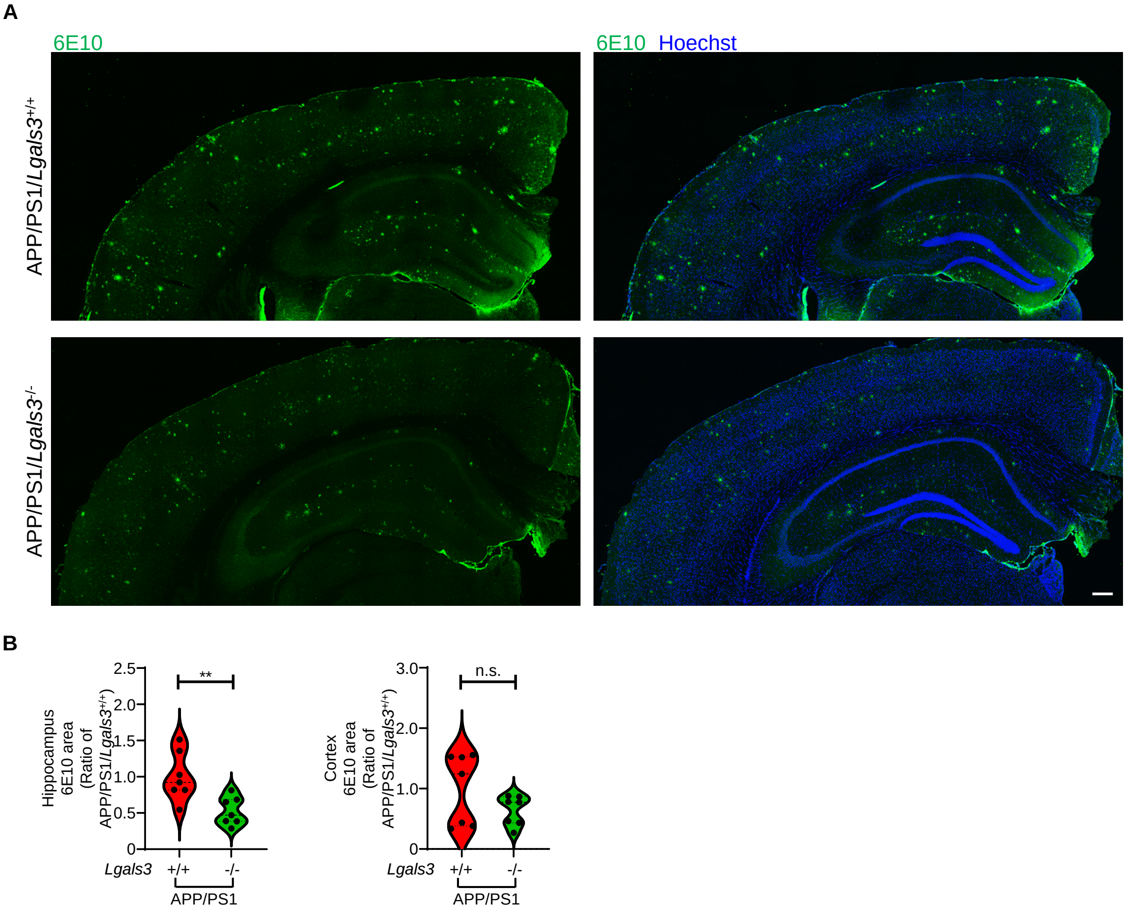

Supplemental Figure 24. Staining of amyloid-beta plaques in APP/PS1/*Lgals3*$^{-/-}$ and APP/PS1/*Lgals3*$^{+/+}$ mice, related to Figure 7. (A-B) Immunohistochemical staining and quantification of 6E10 in the hippocampi and cortexes of Tau22/*Lgals3*$^{-/-}$ and control mice, n=7 mice. Scale bar, 200 µm. Data were analyzed with the two-tailed unpaired *t* test and violin plots show medians with 25th and 75th percentiles, $^{**}P < 0.01$.

Galectin-3 aggravates microglial activation and tau transmission in tauopathy

Jian Jing Siew[1], Hui-Mei Chen[1], Feng-Lan Chiu[2], Chia-Wei Lee[1], Yao-Ming Chang[1], Hung-Lin Chen[1], Thi Ngoc Anh Nguyen[1], Hung-Ting Liao[1], Mengyu Liu[3], Hsiao-Tien Hagar[3], Yung-Chen Sun[4], Hsing-Lin Lai[1], Min-Hao Kuo[3], David Blum[5,6], Luc Buée[5,6], Lee-Way Jin[7], Shih-Yu Chen[1], Tai-Ming Ko[1,8], Jie-rong Huang[4], Hung-Chih Kuo[2], Fu-Tong Liu[1] and Yijuang Chern[1*]

[1]Institute of Biomedical Sciences, Academia Sinica, Taipei, Taiwan

[2]Institute of Cellular and Organismic Biology, Academia Sinica, 11529 Taipei, Taiwan

[3]Department of Biochemistry and Molecular Biology, Michigan State University, 603 Wilson Road, Room 401, Biochemistry Building, East Lansing, MI, 48824, USA

[4]Institute of Biochemistry and Molecular Biology, National Yang Ming Chiao Tung University, No. 155 Section 2 Li-nong Street, Taipei, 11221, Taiwan

[5]Univ. Lille, Inserm, CHU Lille, U1172 - LilNCog - Lille Neuroscience & Cognition, 59000, Lille, France

[6]Alzheimer & Tauopathies, LabEx DISTALZ, LiCEND, 59000, Lille, France

[7]Department of Pathology and Laboratory Medicine, University of California Davis, Sacramento, CA, USA

[8]Department of Biological Science and Technology, National Yang Ming Chiao Tung University, No. 75, Boai Street, Hsinchu 300, Taiwan.

Corresponding author / address reprint requests to the following:
Yijuang Chern
Institute of Biomedical Sciences, Academia Sinica, Nankang, Taipei 115, Taiwan,
Tel.: 886-2-26523913; Fax: 886-2-27829143; E-mail: bmychern@ibms.sinica.edu.tw

Running title: **Microglial galectin-3 aggravates tauopathy**

Key words:
Alzheimer's disease, tauopathy, microglia, galectin, exosomes

Conflicts of interest: The authors have declared that no conflict of interest exists.

Supplemental Methods

Antibodies and reagents. For immunofluorescence staining, the following antibodies were used: anti-IbaI (1:1,000, 019-19741, Wako), anti-galectin-3 (1:300, AF1197, R&D Systems), anti-MC1 (1:500, gift from P. Davies), anti-AT100 (1:100, MN1060, Invitrogen), anti-AT8 (1:500, MN1020, Invitrogen), anti-CD68 (1:100, ab31630, Abcam), anti-Homer1 (1:1,000, 160003, SYSY), anti-VGLUT1 (1:300, 135304, SYSY), anti-CD11b (1:1,000, ab133357, Abcam), anti-TMEM119 (1:50, AMAB91528, Sigma/Atlas), anti-CD45 (1:100, 304002, BioLegend), anti-TREM2 (1:100, 237920, R&D Systems), anti-P2RY12 (1:200, HPA014518, Sigma/Atlas), anti Tau5 (1:1,000, AHB0042, Invitrogen), anti-βIII-tubulin (1:50, MAB1637, Sigma). For immunoblot analysis, anti-galectin-3 (1:1,000, AF1197, R&D Systems), anti-actin (1:5,000, A2066, Sigma),), anti-MC1 (1:1,000, gift from P. Davies), anti-AT100 (1:1,000, MN1060, Invitrogen), anti-Tau5 (1:1,000, AHB0042, Invitrogen), anti-demethylated PP2Ac (1:1,000, 05-577, Millipore), anti-PP2Ac (1:1,000, 05-421, Millipore), anti-phospho-GSK-3α/β (1:1,000, 05-413, Upstate Biotechnology), anti-GSK-3β (1:1,000, GTX83315, GeneTex), anti-phosphor-CaMKII-α (1:1,000, NB300-184, Novus Biologicals), anti- CaMKII-α (1:1,000, LS-C50001, LifeSpan BioSciences), anti-Dectin-1 (1:1,000, ab140039, Abcam), anti-CD63 (1:1,000, 25682-1-AP, Proteintech), anti-CD81 (1:1,000, 66866-1-lg, Proteintech), anti-ALIX (1:1,000, ab117600, Abcam), anti-TSG101 (1:1,000, ab83, Abcam), anti-galectin-3 (1:1,000, MA1-40229, Thermo Fisher Scientific), anti-CD11b (1:1,000, ab133357, Abcam) and anti-Oligomer A11 (1:1,000, AHB0052, Invitrogen) were used. Recombinant Gal3 (1, 2) and pTau (3) were generated as previously described and as detailed in the main text. Other reagents, such as an anti-Gal3 neutralizing antibody (556904, BD

Pharmingen), TD139 (B2266-5, BioVision), and thioflavin S (T1892, Sigma), were purchased from the indicated companies.

Human tissue samples. Postmortem human cortical and hippocampal specimens from four normal subjects, six FTLD-Tau (CBD, PSP, and Pick's) patients and four Alzheimer's disease patients were obtained from the UC Davis Alzheimer's Disease Center, USA (Supplemental Table 1). For immunoblotting, proteins were extracted from formalin-fixed, paraffin-embedded brain specimens according to procedures detailed elsewhere (4). After deparaffinization, the sample pellets were incubated in 20 mM Tris HCl buffer (pH 9) with 2% SDS. After 5 min of incubation on ice, the samples were mixed by vortexing and boiled at 100 °C for 20 min, followed by incubation at 80 °C for 2 hours with intermittent vortexing every 10 min. The samples were pelleted at 16,000 x g for 20 min, and the supernatants were collected for analysis.

Behavior phenotyping. The Morris water maze test was conducted as detailed elsewhere with slight modifications (5). Male mice were used throughout the study. Mice with ages of 10–11 months were trained to locate the hidden platform in a tank filled with opaque water. Mice were trained four times per day in training sessions of two min each for five continuous days. Each quadrant was marked with distinct visual cues on the wall. Probe trial data collected on Day 8 are represented as the time spent in Q4, the quadrant in which the platform had been located in the training sessions. Mouse movements were recorded and analyzed with a TrackMot video tracking system (Diagnostic & Research Instruments Co., Ltd., Taoyuan, Taiwan). The time spent in Q4 in a two-min period of time was compared with the average time spent in other quadrants (a.o.). The researchers were blinded to the mouse genotype throughout the experiments.

Human iPSC-derived microglia (iMGL). Human iPSCs were routinely maintained in Dr. Hung-Chih Kuo's laboratory at the Institute of Cellular and Organismic Biology, Academia Sinica, Taiwan, as previously described (6). The gender and APOE genotype of human iPSCs are listed in Supplemental Table 2. Microglial differentiation was conducted according to an established protocol detailed elsewhere with slight modification (7). Briefly, three independent iPSC lines were adapted to a feeder-free system in mTeSR™1 medium (#85850, STEMCELL Technologies) according to the manufacturer's protocol. For differentiation, 20–40 cell aggregates with a size of approximately 100 cells/aggregate were seeded on Matrigel (356231, Corning)-coated 6-well plates in mTeSR™1 medium containing 0.5 µM thiazovivin (#72254, STEMCELL Technologies). After one day, the medium was replaced with Medium A (#05310, STEMCELL Technologies), and half of the medium was changed after 48 hours. On Day 3, Medium A was replaced with Medium B (#05310, STEMCELL Technologies). Without removing the medium, 1 ml of Medium B was supplemented every other day until Days 10–12. On Day 12, hematopoietic progenitor cells (HPCs, nonadherent cells) were collected for microglial differentiation.

The HPCs were centrifuged at 300 x g for 5 min and seeded on 1 mg/ml Matrigel-coated 6-well plates at a density of 100,000 cells per well in iPS-microglia medium (DMEM/F12 (11039021, Thermo Fisher Scientific) supplemented with 2x ITS (41400045, Thermo Fisher Scientific), 2x B27 (17504044, Thermo Fisher Scientific), 0.5x N2 (17502048, Thermo Fisher Scientific), 1x GlutaMax (35050061, Thermo Fisher Scientific), 1x NEAA (11140050, Thermo Fisher Scientific), 400 µM monothioglycerol (M6145, Sigma), and 5 µg/ml insulin (Sigma, I2643)). At this stage, the iPS-microglia medium was supplemented with three cytokines immediately before use: 100 ng/ml IL34 (200-34, PeproTech), 50 ng/ml TGFβ1 (100-21,

PeproTech) and 25 ng/ml M-CSF (300-25, PeproTech). Without removing the medium, 1 ml of fresh iPS-microglia medium supplemented with a three-cytokine cocktail was added every other day for another 25 days. Throughout differentiation, the cells were predominantly nonadherent; when the medium reached the maximum levels in the well, it was aspirated, and the cells were resuspended in 1 ml of fresh medium with 1 ml of conditioned medium. After 25 days, the cells were resuspended in iPS-medium supplemented with five cytokines, 100 ng/ml IL34, 50 ng/ml TGFβ1 and 25 ng/ml M-CSF, 100 ng/ml CX3CL1 (300-31, PeproTech) and 100 ng/ml CD200 (C311, Novoprotein), to promote microglial maturation. The cells were supplemented with iPS medium with a five-cytokine cocktail every other day for 1 week and harvested for analysis.

Biochemical extraction of mouse tissue. Protein extraction from mouse samples was carried out as detailed previously (8). Hippocampal tissues were homogenized in 10 volumes of H buffer (10 mM Tris-HCl, 1 mM EGTA, 800 mM NaCl, 10% sucrose, 0.1 mM PMSF, 1 mM sodium orthovanadate, 1x protease inhibitor cocktail, 1x phosphatase inhibitor cocktail) by using a micropestle. The homogenates were centrifuged at 20,000 x g for 30 min at 4 °C, and the supernatants were collected. The pellets were extracted again with 5 volumes of H buffer and centrifuged, and each supernatant was combined with the respective previously harvested supernatant. The combined supernatants were supplemented with 1% sarkosyl, incubated for 2 hours at 37 °C, and centrifuged at 300,000 x g. The sarkosyl-soluble supernatants were collected and stored at -80 °C. For the analysis of membrane-bound proteins, the pellets obtained after H buffer treatment were homogenized in RIPA buffer (50 mM Tris-HCl, 0.25% sodium deoxycholate, 1% Triton X-100, 150 mM NaCl, 1x protease inhibitor cocktail and 1x phosphatase inhibitor cocktail) by using 25-gauge needles. The homogenates were then rotated for 1 hour at 4

°C and centrifuged at 16,000 x g for 20 min, and the supernatants were collected and stored at -80 °C.

Immunoblot analysis and filter assay. For sarkosyl-soluble samples, a sample was mixed with an equal volume of 4X sample buffer (8% SDS, 20% 2-mercaptoethanol, 40% glycerol, 0.004% bromophenol blue, and 0.25 M Tris HCl, pH 6.8). For RIPA buffer-soluble samples, protein concentrations were determined by using the Bio-Rad protein assay Dye Reagent Concentrate (#5000006, Bio-Rad). Samples were subjected to SDS–PAGE and transferred electrophoretically to a PVDF membrane. The membrane was blocked with 5% BSA or nonfat milk in TBS containing 0.1% Tween-20 (v/v) (TBST), followed by incubation with the primary antibodies overnight at 4 °C. The detection of oligomeric proteins in the EVs was analyzed in a filter assay using a slot blot apparatus (Bio-Rad). After protein adsorption, the cellulose acetate membrane was blocked with 5% BSA and incubated overnight with an anti-A11 antibody (AHB0052, Invitrogen) at 4 °C. After 5 washes with TBST, the membrane was incubated with the corresponding secondary antibodies for 1 hour at room temperature (RT). After 5 washes with TBST, the immunoblot was visualized by using the enhanced chemiluminescence detection method. Data were analyzed with ImageJ software version 1.52o (NIH, USA). All loading controls were run on the same gel.

Immunofluorescence staining. Immunofluorescence staining was performed as previously described(9). In brief, the experimental animals were anesthetized via the i.p. injection of 80 mg/kg sodium pentobarbital, followed by transcardial perfusion with saline. Collected brain samples were postfixed in 4% (w/v) paraformaldehyde for 3 days at 4 °C and equilibrated in 30% (w/v) sucrose for 2 days. Brain samples were transected coronally at a 20 μm width with a sliding microtome

and stored in 0.1 M phosphate buffer (Na-PB, pH 7.4) with 0.1% sodium azide. Cell samples were fixed in 4% (w/v) paraformaldehyde and 4% (w/v) sucrose in PBS. Unless otherwise stated, brain sections were stained after mounting on slides. For Gal3 staining, antigen retrieval was performed by using citrate buffer, pH 6.0 (C9999, Sigma), at 97.5 °C for 20 min prior to staining. Brain sections and cells were permeabilized with 0.2% (v/v) Triton X-100 and 0.05% (v/v) NP-40, respectively, and blocked in 4% (w/v) BSA for 1 to 2 hours. To study synapses, brain samples were stained under free-floating conditions and preincubated in 1% Triton X-100 (v/v) in 4% (w/v) BSA for 2 hours at RT to make the postsynaptic densities accessible. After extensive washing, the samples were incubated with the primary antibodies at 4 °C in a humidified chamber for 1–2 days, followed by 2 hours of incubation with the corresponding secondary antibodies at RT.

To detect the Gal3 binding sites in brain tissues, recombinant Gal3 was conjugated with Atto-565 fluorescence dye by using a Lightning-Link® Rapid Atto 565 Conjugate system (351-0030, Innova BioSciences) following the manufacturer's protocol, and used to label brain section (^{Atto565}rGal3, 100µg/ml) at 4 °C in a humidified chamber for 1 day. Nuclei were stained with Hoechst 33258. In human brain samples, sections were treated with 0.1% (w/v) Sudan Black B (Sigma) in 70% ethanol for 15 min at RT to block autofluorescence signals after washing following secondary antibody incubation and before nuclear staining. The slides were then analyzed by confocal microscopy (LSM780, LSM880 microscope and Zen 2012 software; Carl Zeiss, Germany). Given that the CA1 has the highest level of hyperphosphorylated tau in THY-Tau22 mice(8), we chose to analyze mainly the CA1 region. The acquired images were processed by using Metamorph Microscopy Automation & Image Analysis software (Molecular Devices, USA).

ELISA. The levels of the inflammatory cytokines IL1α, IL1β, IL6, TNFα and Gal3 in iMCM were determined by using the corresponding mouse DuoSet® ELISA Development System (DY200-05, DY201-05, DY206-05, DY210-05, DY1154, R&D Systems) following the manufacturer's instructions. The optical density at 450 nm was measured with a SpectraMax 190 (Molecular Devices, Sunnyvale, CA, USA) microplate reader, and the value obtained at 570 nm was subtracted as the wavelength correction. The levels of each cytokine and Gal3 were calculated via reference to a standard curve prepared with the appropriate protein standard.

Thioflavin-S fluorescence assay. To monitor pTau aggregation, a thioflavin-S binding assay was performed as detailed previously with slight modification (3). In brief, pTau was diluted to a final concentration of 6 μM with or without rGal3 (2 μg), while ThS was prepared at a 0.25% (w/v) concentration in 50% EtOH. Real-time ThS fluorescence measurements were carried out by using a Gemini EM Fluorescence Microplate Reader System (Molecular Devices, Sunnyvale, CA, USA). Samples were incubated at 37 °C for 20 hours, and measurements were performed every 10 mins. The excitation and emission wavelengths were 440 nm and 490 nm, respectively. All conditions were conducted in triplicate.

Aβ fibril formation. Aβ fibrils were prepared according to procedures detailed elsewhere (10). Recombinant Aβ protein fragment 1-42 was purchased from Sigma (A9810, Sigma). To prepare Aβ fibrils, the Aβ peptide was dissolved in 1 mM hexafluoroisopropanol (HFIF, 105228, Sigma) and aliquoted into sterile microcentrifuge tubes. HFIF was removed by SpeedVac under vacuum conditions. For fibrillization, the peptide was resuspended in DMSO to a concentration of 5 mM,

followed by the addition of 10 mM HCl to a final concentration of 100 μM. The mixture was incubated for 24 hours at 37 °C and subjected to treatment.

NTA. Freshly isolated EVs were resuspended in 300 μl of PBS. For NTA, the samples were further diluted with PBS (1:50), and 300 μl of the diluted samples were loaded into the sample chamber of a NanoSight instrument (NanoSight NS300, Malvern Panalytical) using a disposable syringe. EV sizes and concentrations were analyzed with NTA 3.44 software (NanoSight) according to the manufacturer's protocol.

RNA sequencing and bioinformatic analysis. Total RNA was extracted using the GENEzol TriRNA Pure Kit according to the manufacturer's instructions. The purity and integrity of RNA samples were quantified by using an ND-1000 spectrophotometer (Nanodrop Technology, USA) according to the OD 260/280 ratio and 260/230 ratio and a Bioanalyzer 2100 (Agilent Technology, USA) with an RNA 6000 LabChip kit (Agilent Technology, USA). All RNA sample preparation procedures were carried out according to Illumina's official protocol. For library construction, Agilent's SureSelect Strand-Specific RNA Library Preparation Kit was used, followed by AMPure XP bead (Beckman Coulter, USA) size selection. Sequences were determined by using Illumina's sequencing-by-synthesis (SBS) technology (Illumina, USA). Sequencing data (FASTQ reads) were generated using Welgene Biotech's pipeline based on Illumina's base calling program bcl2fastq v2.20. A Phred quality score of Q20 with a 99% base calling accuracy was applied during sequencing quality control. For BaseCall conversion, Illumina's official base calling software bcl2fastq Conversion Software v2.20 was used to convert BCL files from all Illumina sequencing platforms into FASTQ reads. All adaptor sequences were removed (adaptor clipping). Quality

trimming was performed to remove low-quality reads/bases. Lower quality bases from the 3' end were removed by using a sliding-window approach as the per-base quality gradually decreased toward the 3' ends of reads. Both adaptor clipping and sequence quality trimming were performed using Trimmomatic v0.36 with a sliding-window approach. All sequence alignment was conducted by using HISAT2. The databases employed for mouse and human sample mapping were GRCm39 and GRCh38.p13, respectively. Differential expression analysis was performed by using Cuffdiff (cufflinks v2.2.1) with genome bias detection/correction and Welgene Biotech's in-house pipeline.

The thresholds used to define differentially expressed genes (DEGs) based on the RNA-seq analysis of mouse samples were a $\log_2$ ratio fold-change greater than or equal to 0.3 or lower than or equal to -0.3 and a *P* value of less than 0.05, and those for iMGL samples were a $\log_2$ ratio fold-change greater than or equal to 0.55 or lower than or equal to -0.55 and a *P* value of less than 0.05. The smallest *P* value was limited to $P = 0.00005$. Genes that met these significance thresholds were separated into upregulated or downregulated genes and applied as the input for functional enrichment analysis. Several database sources were referenced for pathway enrichment analysis, including the Gene Ontology (GO) and Kyoto Encyclopedia of Genes and Genomes (KEGG) databases, using DAVID v6.8 and canonical pathway analysis in Ingenuity Pathway Analysis software (IPA; QIAGEN Inc., https://www.qiagenbioinformatics.com/products/ingenuity-pathway-analysis) (11). Unless otherwise stated, pathway with false discovery rate (FDR) less than 0.05 was identified as enrichment. Data visualization of the RNA-seq analysis results was conducted with open-source software suites (Morpheus and Instant Clue). Heatmaps were generated with Morpheus (https://software.broadinstitute.org/morpheus). Scatterplots and volcano plots were generated with Instant Clue (12).

scRNA-seq of microglia. Adult microglial isolation was performed according to the protocol detailed elsewhere (13). In brief, WT and Tau22 mice (n= 8 in each group) were transcardially perfused with saline. The dissected hippocampal tissues were homogenized with a glass douncer in cold medium A (15 mM HEPES, 0.5% glucose in HBSS without phenol red) supplemented with DNase (1:25) and an RNase inhibitor (1:400; Clontech 2313B). The homogenates were filtered through a 70 μm strainer (Falcon 352350) and pelleted at 400 x g for 5 min (4 °C). Pellets were resuspended in 500 μl MACS buffer (0.5% BSA and 2 mM EDTA in HBSS) with an RNase inhibitor (1:500) and myelin removal beads (1:10; Miltenyi Biotec 130-096-433). After 10 min of incubation on ice, the volume of the samples was adjusted to 2 ml before loading into LS columns (Miltenyi Biotec 130-042-401). The resultant myelin-depleted cell suspensions were filtered into 35 μm FACS tubes (Falcon 352235) and pelleted at 400 x g for 5 min (4 °C). Cell pellets were resuspended in FACS buffer (10% FBS in PBS) and incubated with Fc block (1:60; BD Pharmingen 553142) for 5 min on ice.

To label microglia, samples were incubated with anti-CD11b-BV421 (1:300; BioLegend 101236) for 10 min, pelleted and resuspended in FACS buffer in the presence of an RNase inhibitor (1:500) and propidium iodide (1:1,000; Invitrogen P3566) immediately before sorting. Cells were sorted on a flow cytometer (FACSAria IIIu – 15 color cell sorter). The live CD11b-positive fraction was sorted into cold PBS with 10% FBS. The FACS procedures were performed by the Flow Cytometry Core Facility (AS-CFII108-113) at IBMS, Academia Sinica, Taiwan.

For single-cell gene expression library construction, FACS-sorted cells were immediately captured in droplets that were emulsified with gel beads by using the 10x Genomics Chromium Next GEM Single Cell 3' GEM, Library & Gel Bead Kit v3.2 reagent (10x Genomics, USA)

according to the manufacturer's protocols (10x Genomics, USA). These libraries were sequenced using a NovaSeq 6000 (Illumina, USA).

scRNA-seq bioinformatic analysis. The scRNA-seq output files were processed with Cell Ranger software (3.1.0, 10x Genomics). Sequencing reads were mapped to the mouse genome (mm10) and Ensembl gene annotation (release 102) by filtering out genes with the following biotypes: IG gene, ncRNA, TEC, and TR gene. A total of 12,430 and 16,220 cells were obtained from the hippocampus of WT and Tau22 mice, respectively. In summary, 1,142 and 1,835 median genes/cell, 1,934 and 3,734 median UMIs/cell and 39,808 and 27,396 mean reads/cell were detected in WT and Tau22, respectively. Over 494 and 444 million reads were detected in cells from WT and Tau22 mice, respectively. Downstream gene profile analyses were performed using the Seurat package in R Seurat 4.0.4 (14). The dataset was filtered according to the following criteria: (1) cells with UMI counts below 200 or above 6,000 were removed; (2) cells that showed fewer than 200 genes were removed; and (3) cells that showed more than 10% mitochondrial genes were removed. In addition, genes that showed expression in fewer than 3 cells were ignored. After data filtering and removing outliers, we obtained 12,413 and 16,183 cells from WT and Tau22 mice, respectively. In total, we obtained 11,484 and 14,773 microglia from WT and Tau22 mice, respectively. The data were normalized by log transformation followed by regression based on total UMI counts and mitochondrial gene content. Genes associated with principal components (1 to 30) were used for data dimensional reduction based on UMAP to generate distinctive cell clusters. For GAM gene analysis, a total of 497 cells with *Lgals3* greater than or equal to one average log UMI count expression levels were identified as from all clusters and binned together as the *Lgals3*-positive cluster (Supplemental Figure 16A). Data visualization in heatmaps, UMAP

plots, violin plots, and ridge plots was performed using the built-in functions of Seurat 4.0 as follows: 'DoHeatmap', 'FeaturePlot', 'VlnPlot', and 'RidgePlot', respectively. Pseudotime cell trajectories of microglia were obtained by using Monocle3. Microglia Clusters 1 and 2 were selected as the root of the trajectories. GO gene networks were created with REVIGO (15) and visualized with the WordCloud app in Cytoscape 3.8 (16). The DEGs (| log2-fold-change | $\geq$ 0.25) of each cluster were identified with "FindMarkers" and were subjected to GO analysis to identify the major biological processes. Pathway with FDR less than 0.05 was identified as enrichment.

Supplemental References

1. Hsu DK, Zuberi RI, and Liu FT. Biochemical and biophysical characterization of human recombinant IgE-binding protein, an S-type animal lectin. *J Biol Chem.* 1992;267(20):14167-74.
2. Chiu YP, Sun YC, Qiu DC, Lin YH, Chen YQ, Kuo JC, and Huang JR. Liquid-liquid phase separation and extracellular multivalent interactions in the tale of galectin-3. *Nat Commun.* 2020;11(1):1229.
3. Liu M, Sui D, Dexheimer T, Hovde S, Deng X, Wang KW, et al. Hyperphosphorylation Renders Tau Prone to Aggregate and to Cause Cell Death. *Mol Neurobiol.* 2020;57(11):4704-19.
4. Guo H, Liu W, Ju Z, Tamboli P, Jonasch E, Mills GB, et al. An efficient procedure for protein extraction from formalin-fixed, paraffin-embedded tissues for reverse phase protein arrays. *Proteome Sci.* 2012;10(1):56.
5. Chang CP, Chang YG, Chuang PY, Nguyen TNA, Wu KC, Chou FY, et al. Equilibrative nucleoside transporter 1 inhibition rescues energy dysfunction and pathology in a model of tauopathy. *Acta Neuropathol Commun.* 2021;9(1):112.
6. Chiu FL, Lin JT, Chuang CY, Chien T, Chen CM, Chen KH, et al. Elucidating the role of the A2A adenosine receptor in neurodegeneration using neurons derived from Huntington's disease iPSCs. *Hum Mol Genet.* 2015;24(21):6066-79.
7. McQuade A, Coburn M, Tu CH, Hasselmann J, Davtyan H, and Blurton-Jones M. Development and validation of a simplified method to generate human microglia from pluripotent stem cells. *Mol Neurodegener.* 2018;13(1):67.
8. Ising C, Venegas C, Zhang S, Scheiblich H, Schmidt SV, Vieira-Saecker A, et al. NLRP3 inflammasome activation drives tau pathology. *Nature.* 2019;575(7784):669-73.
9. Siew JJ, Chen HM, Chen HY, Chen HL, Chen CM, Soong BW, et al. Galectin-3 is required for the microglia-mediated brain inflammation in a model of Huntington's disease. *Nat Commun.* 2019;10(1):3473.
10. Dahlgren KN, Manelli AM, Stine WB, Jr., Baker LK, Krafft GA, and LaDu MJ. Oligomeric and fibrillar species of amyloid-beta peptides differentially affect neuronal viability. *J Biol Chem.* 2002;277(35):32046-53.
11. Kramer A, Green J, Pollard J, Jr., and Tugendreich S. Causal analysis approaches in Ingenuity Pathway Analysis. *Bioinformatics.* 2014;30(4):523-30.
12. Nolte H, MacVicar TD, Tellkamp F, and Kruger M. Instant Clue: A Software Suite for Interactive Data Visualization and Analysis. *Sci Rep.* 2018;8(1):12648.
13. Tabula Muris C, Overall c, Logistical c, Organ c, processing, Library p, et al. Single-cell transcriptomics of 20 mouse organs creates a Tabula Muris. *Nature.* 2018;562(7727):367-72.
14. Hao Y, Hao S, Andersen-Nissen E, Mauck WM, 3rd, Zheng S, Butler A, et al. Integrated analysis of multimodal single-cell data. *Cell.* 2021;184(13):3573-87 e29.
15. Supek F, Bosnjak M, Skunca N, and Smuc T. REVIGO summarizes and visualizes long lists of gene ontology terms. *PLoS One.* 2011;6(7):e21800.
16. Shannon P, Markiel A, Ozier O, Baliga NS, Wang JT, Ramage D, et al. Cytoscape: a software environment for integrated models of biomolecular interaction networks. *Genome Res.* 2003;13(11):2498-504.
